# Exploring the performance of nanostructured reagents with organic-group-defined morphology in cross-coupling reaction

**DOI:** 10.1038/s41467-018-05350-x

**Published:** 2018-07-26

**Authors:** Alexey S. Kashin, Evgeniya S. Degtyareva, Dmitry B. Eremin, Valentine P. Ananikov

**Affiliations:** Zelinsky Institute of Organic Chemistry, Russian Academy of Sciences, Leninsky Prospect, 47, Moscow, Russia 119991

## Abstract

The great impact of the nanoscale organization of reactive species on their performance in chemical transformations creates the possibility of fine-tuning of reaction parameters by modulating the nano-level properties. This methodology is extensively applied for the catalysts development whereas nanostructured reactants represent the practically unexplored area. Here we report the palladium- and copper-catalyzed cross-coupling reaction involving nano-structured nickel thiolate particles as reagents. On the basis of experimental findings we propose the cooperative effect of nano-level and molecular-level properties on their reactivity. The high degree of ordering, small particles size, and electron donating properties of the substituents favor the product formation. Reactant particles evolution in the reaction is visualized directly by dynamic liquid-phase electron microscopy including recording of video movies. Mechanism of the reaction in liquid phase is established using on-line mass spectrometry measurements. Together the findings provide new opportunities for organic chemical transformations design and for mechanistic studies.

## Abstract (web summary)

Direct visualization and characterization of the influence of nanoscale organization on the reactivity of organic functional groups in solution remain a challenge. Here, the authors report the real-time observation of the involvement of a nanostructured reagent in a cross-coupling reaction using advanced liquid-phase electron microscopy.

## Introduction

Outstanding progress in the field of nanostructured matter has significantly expanded the possibilities to design materials for solar cells, energy research, photonics, biomedical applications and other demanding areas^1–11^. Utilization of nanostructured materials in the catalyst design has revolutionized chemical synthesis and has facilitated the development of new technologies^12–18^. Size-dependent properties of metal nanoparticles and nanoscale organization of organic systems have made a substantial impact on biomass conversion, fine chemical synthesis, pharmaceutical science, and preparation of drugs and biologically active molecules, among many other areas^19–31^.

The concept of tuning the reactivity at the molecular level via nanoscale organization has recently expanded to control the reactivity of functionalized organic and organometallic compounds^32–39^. A challenging area of ongoing research in organic synthesis addresses the cross-coupling methodology for carbon–carbon and carbon–heteroatom bond formation^40–48^, where nanomaterials may provide an additional dimension to transfer unique nanoscale-driven phenomena into molecular organic architectures. We have recently shown that 1D-nanostrucutured metal thiolates and their functional derivatives provide an efficient opportunity for selective catalytic carbon-heteroatom bond formation^49,50^. Such reactions are of primary importance for the incorporation of sulfur-containing functional groups into organic electronics, polymeric materials, and pharmaceutical substances^51–57^.

**Fig. 1:**
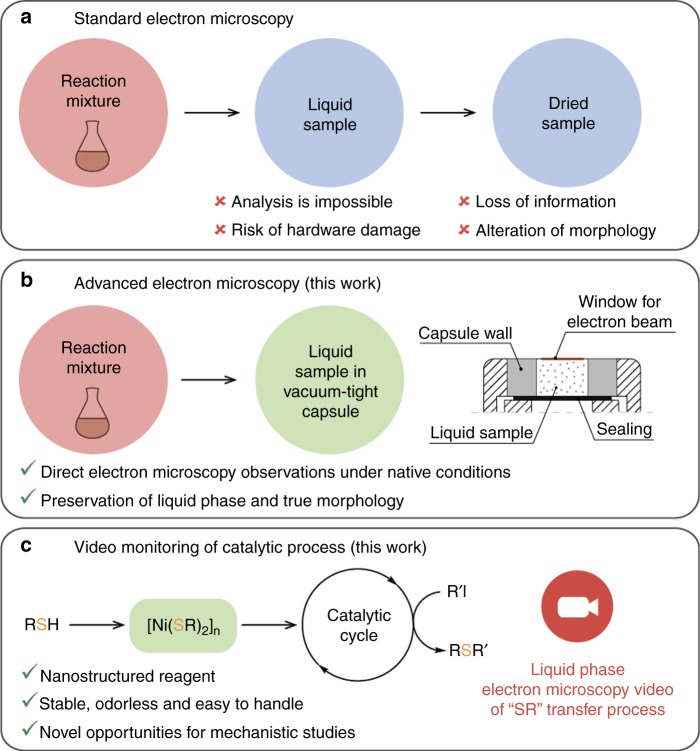
Electron microscopy studies of the catalytic reactions. Standard solid-phase electron microscopy (**a**), advanced liquid-phase electron microscopy approach (**b**); and application of liquid-phase electron microscopy for observation of nanostructured reagent behavior in catalytic cross-coupling reaction (**c**)

The impossibility of direct visualization and characterization of the influence of nanoscale organization on the reactivity of organic functional groups in solution has presented a stumbling block in this area for a long time. Electron microscopy is the analytic tool of choice for the visualization of nanoscale processes, but routinely it is applied only to solid systems. Liquid samples cannot be studied directly under high vacuum due to solvent evaporation leading to the loss of information and incorrect morphologies (Fig. 1a). Thus, catalytic organic transformations could not be studied because the synthesis of functionalized organic molecules occurred exclusively in the solution phase. Recently, an amazing progress has been achieved in microscopy studies of nanoscale processes in vacuum-tight capsules, providing a fascinating mechanistic tool (Fig. 1b)^58–64^.

In the present article, we report the real-time observation of the involvement of a nanostructured reagent in a cross-coupling reaction (Fig. 1c) performed by advanced liquid-phase electron microscopy (Fig. 1b). Nickel thiolates [Ni(SR)_2_]_*n*_ with nanoscale organization are used as coupling partners in the catalytic C–S bond formation reaction. The transfer of reactive groups from the surface of the reagent particles into the solution is captured by Field-Emission Scanning Electron Microscopy (FE-SEM) images and video. Direct liquid-phase microscopic monitoring of the C–S cross-coupling reaction, as well as Electrospray Ionization Mass Spectrometry (ESI-MS) and kinetic measurements in solution reveal the key features of the nanostructured reagents behavior in organic reaction mixtures and lead to the development of the approach to application of such materials in chemical transformations.

## Results and discussion

### Synthesis and characterization of nickel thiolates

**Fig. 2:**
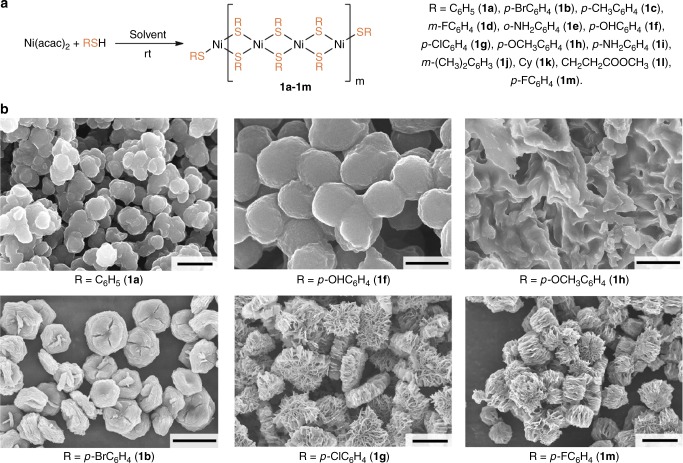
Synthesis and characterization of nickel thiolates. Scheme of reaction between Ni(acac)_2_ and thiols (**a**) and FE-SEM images for selected nickel thiolates with different types of morphology (**b**). Scale bars–500 nm for **1f**, 1 μm for **1a**, 5 μm for **1b** and **1h**, 10 μm for **1g** and **1m**

A series of nanostructured nickel thiolates [Ni(SR)_2_]_*n*_ (**1a**–**1m**) was obtained via a simple ligand substitution reaction involving readily available Ni(acac)_2_ and various thiols (Fig. 2a). The composition and structure of the synthesized nickel thiolates were fully established (see Supplementary Methods for the synthetic procedures and Supplementary Figs. 1–48 for SEM and energy dispersive X-ray spectroscopy (EDX) characterization). Electron microscopy analysis of the prepared substances revealed amazing diversity of morphology of the nickel thiolates (Fig. 2b). It was found that even small variations of substituents in thiols (RSH) resulted in dramatic changes of morphologies of the metal thiolates. Observed micro-structures can be divided into four groups: structures with irregular morphology, small aggregated particles, large meshy particles and structures with exceptional biconcave morphology. For example, for the unsubstituted nickel thiophenolate (**1a**) and thiolate with R=*p*-OHC_6_H_4_ (**1f**) the formation of small particles with clearly visible edges and sizes about 0.5–1 μm was detected (Fig. 2b). The change of the *p*-OH substituent to the *p*-OCH_3_ group (compound **1h**) led to the complete alteration of the morphology with the formation of large dense particles with fused irregular structures (Fig. 2b). Contrariwise, the introduction of a halide substituent into the aromatic ring of thiolate resulted in the formation of sheets that were associated in very incompact meshy structures with a highly developed surface (for *p*-F and *p*-Cl, **1m** and **1g**), and biconcave erythrocyte-like particles (*p*-Br, **1b**). In this case, the size of individual particles was larger and reached 5–15 μm (Fig. 2b). Thus, the substituents in the organic group do define the size and shape of the particles which obviously have a large impact on the reactivity of nano-scale materials.

**Fig. 3:**
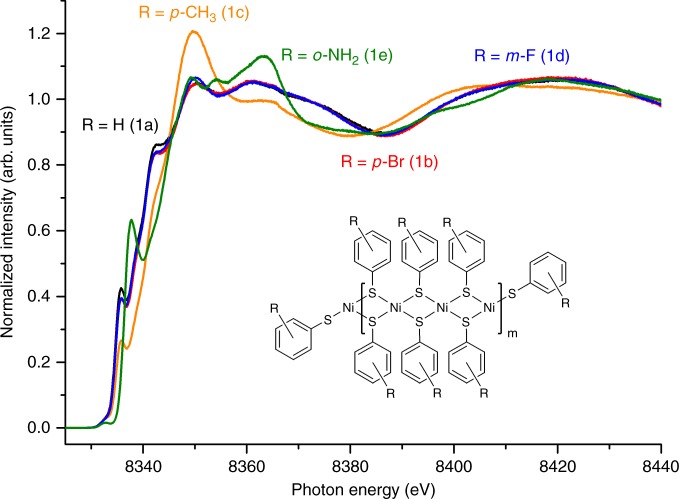
XAS analysis of selected nickel thiolates. Ni K-edge HERFD-X-ray absorption near edge structure (XANES) spectra of nickel thiophenolate (**1a**) and nickel thiolates with *p*-Br (**1b**), *p*-CH_3_ (**1c**), *m*-F (**1d**), and *o*-NH_2_ (**1e**) substituents in the aromatic rings

In order to reveal the structure of the intrinsic building units of the thiolate particles, we investigated five different types of nickel particles (**1a**–**1e**) with the use of Kβ_1,3_-detected high energy resolution fluorescence detected (HERFD) X-ray absorption spectroscopy (XAS) on a high-brilliance synchrotron radiation source. The electronic structure of the nickel thiolates was studied at the Ni K-edge (Fig. 3).

The presence of splitted main absorption lines in all the spectra, except the spectrum of compound **1c**, clearly demonstrated the square planar geometry of Ni^2+^^65–67^. For compounds **1a**, **1b**, and **1d**, the profiles of the absorption lines were almost identical, so it could be concluded that the nature of the outlying substituent had no notable impact on the geometry and electronic properties of the nickel-containing core. The shift of the absorption line for compound **1e** indicated the formation of a nickel coordination compound, where the Ni atom environment contained both sulfur and nitrogen atoms, so the formation of the desired coordination polymer did not occur. Surprisingly, the spectrum of nickel thiolate containing *p*-CH_3_ substituents (**1c**) was completely different from the spectra of the other compounds. The appearance of an intense unsplit main absorption line (white line) indicated the existence of a notable amount of non-planar (possibly octahedral) Ni^2+^ centers along with square planar Ni^2+^ centers. Probably, the non-planar Ni^2+^ centers existed at defects or edges of the nickel thiolate particles, whereas the uniform areas of the particles consisted of planar units. The ratio between these two types of metal centers possibly correlates with the degree of particle ordering, so in the case of **1c**, the formation of highly disordered particles can be supposed. The results of the XAS study showed that the main building unit of the nickel thiolates was the square planar Ni^2+^ centers surrounded by four sulfur-containing groups. Nevertheless, additional coordination of SAr groups to nickel atoms and chelation in some specific cases played a notable role in the formation of nickel thiolate particles.

### Liquid-phase SEM video of C–S cross-coupling reaction

**Fig. 4:**
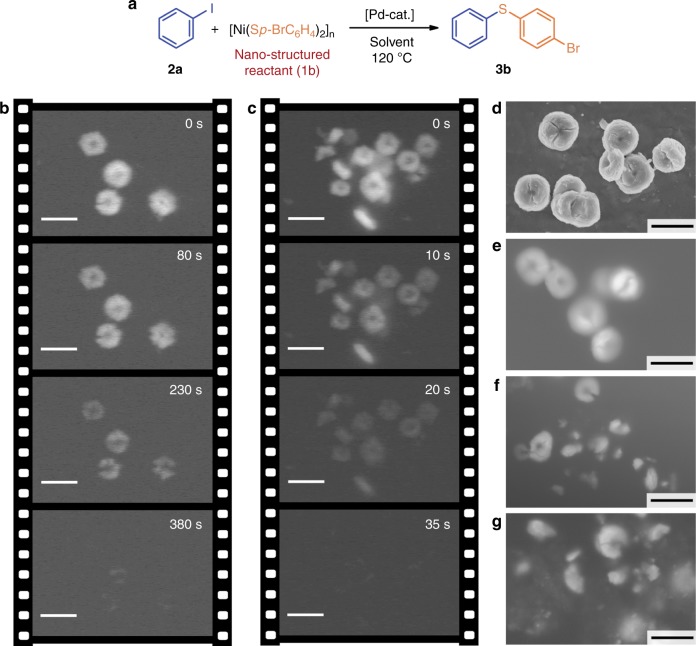
Liquid-phase SEM monitoring of the C–S cross-coupling reaction. Model C–S cross-coupling reaction between **1b** and iodobenzene (**a**). Snapshots taken from SEM videos of dissolution of thiolate **1b** particles in Pd(OAc)_2_/dppe system in the absence (**b**) and in the presence (**c**) of iodobenzene; FE-SEM images of **1b** particles in the solid phase (**d**) and in isopropanol suspension (**e**); and liquid-phase FE-SEM images of the C–S cross-coupling reaction mixture containing **1b** after 45 min (**f**) and 2 h (**g**) of the reaction. Scale bars–5 μm for all images

A unique metal organic erythrocyte-like morphology of the nickel thiolate with a bromine substituent [Ni(S*p*-BrC_6_H_4_)_2_]_*n*_ (**1b**) provided an excellent opportunity for real-time dynamic FE-SEM observations. C–S cross-coupling with iodobenzene (**2a**) was selected as a model reaction (Fig. 4a). It proceeded smoothly in the presence of the Pd(OAc)_2_ catalyst and phosphine ligand (PPh_3_ or dppe), leading to the corresponding sulfide product (**3b**) in up to 95% yield (see Supplementary Methods for reactions description).

At the first step of the liquid-phase FE-SEM observations, we used C–S cross-coupling conditions with a metal acetate/phosphine system for video monitoring of the nickel thiolate disassembling process. It was found that a Pd(OAc)_2_/dppe solution in ethanol (0.02 M metal acetate and 0.04 M dppe) reacted with [Ni(S*p*-BrC_6_H_4_)_2_]_*n*_ particles leading to their dissolution within 6–7 min. At the same time, electron beam irradiation of the reaction mixture for a relatively long time did not lead to detectable side processes. Snapshots taken from the recorded FE-SEM video for the Pd(OAc)_2_/dppe system are shown in Fig. 4b (see Supplementary Movie 1 and also Supplementary Fig. 65 for additional snapshots).

The reactions were conducted under mild conditions in a controlled manner using commercially available Quantomix^TM^ QX102 capsules for in situ SEM^68^, so the behavior of individual particles could be easily monitored. At the beginning, cracks grew on the particles surface, but the overall size of the particles did not change notably. Further, continuous growth of defects within the particles led to the detachment of small fragments (approximately 1/5–1/3 of the initial particle size), but the effective diameter of the combined fragments was still close to that of the thiolate discs before the reaction. After that, all the detached fragments dissolved evenly with the formation of a uniform solution (Fig. 4b). In the presence of the second coupling reagent, iodobenzene (**2a**, 0.4 M concentration), in the studied system, the rate of dissolution increased significantly. A complete disappearance of the nickel thiolate particles was observed after only 35 s of the reaction (Fig. 4c). Thus, iodobenzene played the role of the promoter of the disassembling process activating the initial palladium species or directly participating in the interphase C–S cross-coupling (see Supplementary Movie 2 and also Supplementary Fig. 66 for additional snapshots).

At the next step, monitoring of the C–S cross-coupling in synthetic transformation was performed by direct study of the catalytic system from the flask. Nickel thiolate **1b** and iodobenzene were used as reagents, and a Pd(OAc)_2_/PPh_3_ system was employed as a catalyst. Samples were taken after 45 min and after 2 h from the beginning of the reaction. Solid nickel thiolate and its suspension in isopropanol were used as reference samples (Fig. 4d–g). FE-SEM showed that the biconcave disc shape (4–5 μm in size) with characteristic cracks on the surface was stable and retained the morphology in the solid state and in liquid media (Fig. 4d, e). The FE-SEM study revealed that the dissolution of the thiolate particles during the reaction was not uniform. In the reaction, the original particles broke into subunits with sizes of approximately 1–2 μm (Fig. 4f, g). This observation suggests that particles of **1b** had a complex structure with a number of potentially reactive points, and the reaction occurred first at the defect sites, leading to particle cracking and fragment detachment. The small particle fragments dissolved evenly (for additional SEM images see Supplementary Figs. 67 and 68).

### Exploring reaction with nickel thiolates at molecular level

**Fig. 5:**
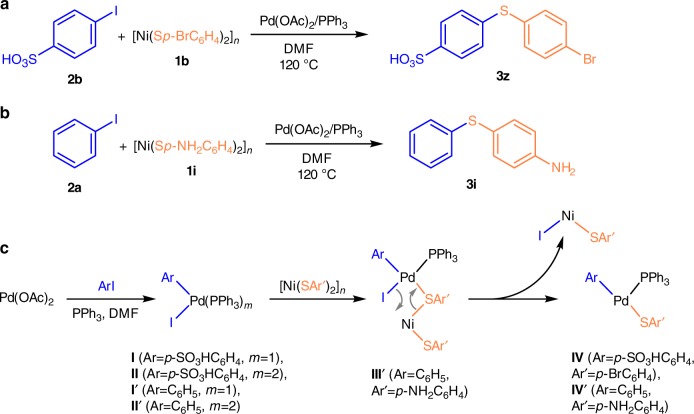
ESI-MS study of the reaction mechanism in solution. Model reactions used for on-line ESI-MS monitoring in negative (**a**) and positive (**b**) ion modes, and intermediates detected in ESI-MS experiments (**c**). Gray arrows (intermediate **III’**) show the cleavage and formation of chemical bonds during transmetallation process

Inspired by the fascinating microscopy study, we investigated the processes occurring in the C–S cross-coupling reaction with nickel thiolates at molecular level. Mass spectrometry is a well-established technique for detection of organic and organometallic compounds even at low concentrations^69,70^. On-line ESI-MS monitoring of the reaction progress allows to study the dynamic behavior of reactive species in solution. In order to increase the sensitivity of the method, charge-tagged reactants were employed as coupling partners (Fig. 5). Organic halide with an easily deprotonating *p*-SO_3_H substituent (**2b**) was used for the reaction study in a negative ion mode (Fig. 5a) and nickel thiolate with an amino group (**1i**) prone to protonation was used for the reaction monitoring in a positive ion mode (Fig. 5b). A number of plausible organometallic intermediates were detected in solution (Fig. 5c).

In the case of the reaction monitoring in a negative ion mode (Fig. 5a), the reactants were initially stirred at 120 °C for 1 min, followed by sequential addition of the ligand (PPh_3_) and Pd(OAc)_2_ catalyst with 1 min delay. Mass spectrometry showed the absence of the reaction without the palladium catalyst and phosphine ligand, which allowed excluding the possibility of the non-catalytic reaction proceeding during the electrospraying process. Once the palladium source was added, the reaction started, which resulted in a decrease of the signal at *m/z* 282 and an increase of the signal at *m/z* 344 (see Supplementary Fig. 69), corresponding to reactant (**2b**) and product (**3z**), respectively (see Supplementary Figs. 70–78 for spectra of all the ions described here and below). The reaction proceeded in less than 1 min notwithstanding the low concentration of the reactants (3.5 × 10^–4^ M).

**Fig. 6:**
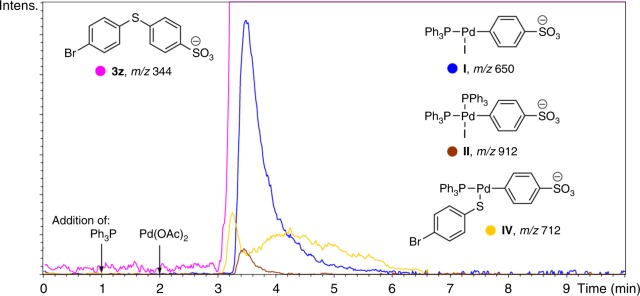
ESI-MS study in the negative ion mode. Intermediate ions detected during on-line monitoring of the reaction between 4-iodobenzenesulpfonic acid (**2b**) and nickel thiolate **1b**

The wide dynamic range and high sensitivity of a time-of-flight mass spectrometer allowed us to detect several key palladium-containing intermediates of the studied reaction, thus elucidating its mechanism. The first detected intermediate at *m/z* 712 (intermediate **IV**) was the result of oxidative addition of aryl iodide to palladium and subsequent transmetallation: the palladium atom with aryl fragments from **1b** and **2b** and the PPh_3_ ligand (Fig. 6). This ion behaved not in a straightforward manner, demonstrating two maxima of intensity. The second group of signals appeared next and referred to oxidative addition products. It should be noted that the ion with only one phosphine ligand at *m/z* 650 (intermediate **I**) was more abundant during the reaction, as compared to the bisphosphine complex (intermediate **II**) at *m/z* 912 (Fig. 6). The characteristic behavior of the signals with time clearly evidenced their intermediate nature, as they rose when the reaction product came to the scene and slowly dropped down within the reaction progress (Fig. 6). On the basis of these observations, one can conclude that the cross-coupling reaction involves a contribution of in-solution (homogeneous) and on-surface (heterogeneous) modes. At the beginning of the reaction, the heterogeneous process at the solid-liquid interface prevailed over the homogeneous one and only the last intermediate of transmetallation released in the solution was detected by ESI-MS. After the partial disassembling of nickel thiolate particles, the reaction entered the homogeneous mode and intermediates were accumulated in the liquid phase. Importantly, this ESI-MS result clearly showed the role of the transmetallation process in the nickel thiolate disassembling.

**Fig. 7:**
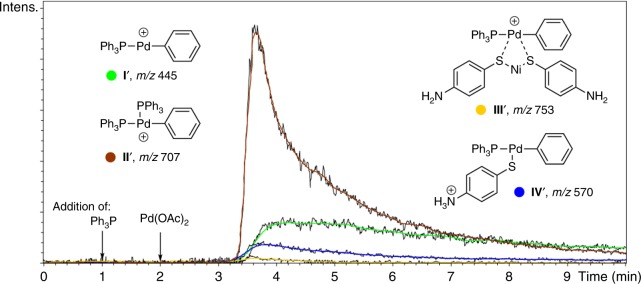
ESI-MS study in positive ion mode. Intermediate ions detected during on-line monitoring of the reaction between iodobenzene (**2a**) and nickel thiolate **1i**. Black curves represent experimental raw data, colored curves correspond to Gauss smoothed representation (3 sec step, 5 cycles)

For independent confirmation, we performed the experiments in a positive ion mode. An amino group with a charge-bearing potential was introduced into thiophenolate reagent **1i** (Fig. 5b). Key intermediates were clearly detected mainly due to ionization via the dehalogenation process. The ions appeared in a spectrum only after injection of both catalyst components (palladium acetate and PPh_3_) into the reaction flask (Fig. 7). A monophosphine complex of oxidative addition at *m/z* 445 (intermediate **I’**) and a bisphosphine complex (intermediate **II’**) at *m/z* 707 were clearly observed (Fig. 7). The product of transmetallation (intermediate **IV’**) was detected at *m/z* 570 (Fig. 7). In this case, ionization was achieved with the help of the functionality due to the good proton affinity of the amine group. An additional intermediate was observed, as compared to the ESI-MS study in a negative ion mode. A low intensity signal at *m/z* 753 corresponded to a bimetallic Pd–Ni complex (intermediate **III’**)–the product of the reaction of intermediate **I’** with nickel thiolate (Fig. 7).

As a result of the mass spectrometry study, key intermediates of the C–S cross-coupling reaction were detected in the solution. Both heterogeneous and homogeneous reaction pathways were traced on the basis of ESI-MS measurements.

### Structure-reactivity correlation for nickel thiolates

**Fig. 8:**
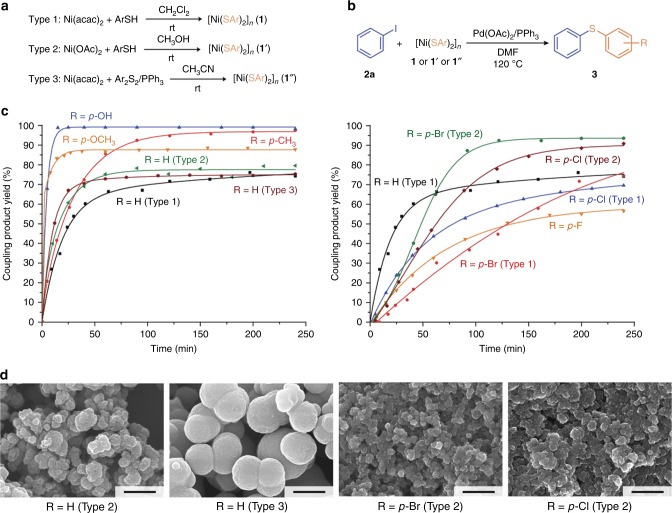
Kinetic measurements for C–S cross-coupling reactions with nickel thiolates. Synthesis of different types of thiolates (**a**); C–S cross-coupling reaction conditions (**b**); obtained kinetic curves (**c**, thiolate of Type 1, was used unless specified otherwise); FE-SEM images of nickel thiolates of types 2 and 3 (**d**). Scale bars–500 nm for all images

The successful mechanistic insight into the C–S cross-coupling reaction with nano-structured reagents revealed the key processes occurring in the solution and on the surface of the solid reagent. At the next step, we performed kinetic measurements in order to reveal the contribution of nano-scale and molecular level properties in the reactivity of nickel thiolates (Fig. 8).

For this purpose, we synthesized a number of nickel thiolates with electron donating and electron withdrawing groups with the use of the above-described procedure. Additionally, in order to vary the morphology of thiolates, we carried out syntheses using Ni(OAc)_2_ as a starting material or Ar_2_S_2_ as a sulfur group source (Fig. 8a, see Supplementary Methods for detailed synthetic procedures and Supplementary Figs. 49–64 for SEM/EDX characterization). All the synthesized compounds were tested in the model C–S cross-coupling reaction catalyzed by a palladium acetate/PPh_3_ system (Fig. 8b).

Kinetic measurements showed the influence of the nature of the substituent in the aromatic rings of thiolates on their performance in the C–S cross-coupling reaction (Fig. 8c). Electron-donating substituents like *p*-OH, *p*-OCH_3_, and *p*-CH_3_ groups increased the reactivity of the thiolate, as compared to the unsubstituted [Ni(SPh)_2_]_*n*_ reactant, favoring the coupling process possibly due to the increase of the negative charge on the sulfur atoms. High yields of the products up to 99% could be reached within first 20 min for *p*-OH and *p*-OCH_3_. For the *p*-CH_3_ substituent with weaker donating properties the reaction occurred in 120 min. On the contrary, the introduction of electron-withdrawing groups into the aromatic rings of the thiolates led to a significant drop in the reaction rate. The activity of the thiolate reagent decreased in the case of halide substituents like *p*-F, *p*-Cl, and *p*-Br. The continuous slow growth of the product yields was observed for all 4 h of the reaction monitoring. However, it should be noted that all the nickel thiolates containing halogen atoms in aromatic rings consisted of relatively large particles, as compared to other thiolates (Fig. 2b), and the size effect obviously could not be neglected.

In order to estimate the influence of the morphology of thiolates on their performance in the C–S bond formation reaction, we tested the series of thiolates with the same [Ni(SAr)_2_]_*n*_ composition, but with different particle sizes and shapes. Thiolate **1a**, which was prepared with the use of the standard procedure, consisted of small particles with figured edges and sizes about 0.5–1 μm (Fig. 2b). When the nickel precursor was changed from Ni(acac)_2_ to Ni(OAc)_2_, the size of the particles decreased to 100–300 nm with the preservation of the shape (Type 2, Fig. 8d). In the case of the thiolate synthesis from disulfide (Ph_2_S_2_) in the presence of PPh_3_ as reducing agent, the compound with round particle morphology and sizes about 0.5–1 μm was obtained (Type 3, Fig. 8d). All the prepared particles were used for the cross-coupling reaction. Results of the kinetic measurements demonstrated a visible difference in the activity of thiolates (Fig. 8c). The particles with small sizes (Type 2) and a regular shape (Type 3) had higher activity in the studied transformation. A remarkable effect was detected in the case of significant alterations in the thiolate morphology. By using Ni(OAc)_2_-based synthesis, it was possible to change the biconcave morphology of the *p*-Br-substituted thiolate and meshy morphology of the *p*-Cl-substituted one (Fig. 2b). The prepared compounds (Type 2, Fig. 8d) consisted of small particles of 50–100 nm in diameter with a distorted spherical shape. Such a decrease in the particle sizes led to a significant increase in the activity of the thiolates. In both cases, the reaction rate increased significantly, and the product yield reached 85–95% within 2–3 h (Fig. 8c).

On the basis of the observed tendencies, one can conclude that the electronic properties of the substituent in the aromatic rings of thiolates affect their activity in the cross-coupling reaction. Electron-rich thiolates reacted with high rates, while electron-deficient ones (with halogen substituents) acted relatively slowly. At the same time, the morphological factor plays a very important role in the process involving a nanostructured reagent. Moreover, the high activity of nickel thiolates can be achieved by changing the particle morphology even in the case of the negative substituent effect.

### Cross-coupling between organohalides and nickel thiolates

**Fig. 9:**
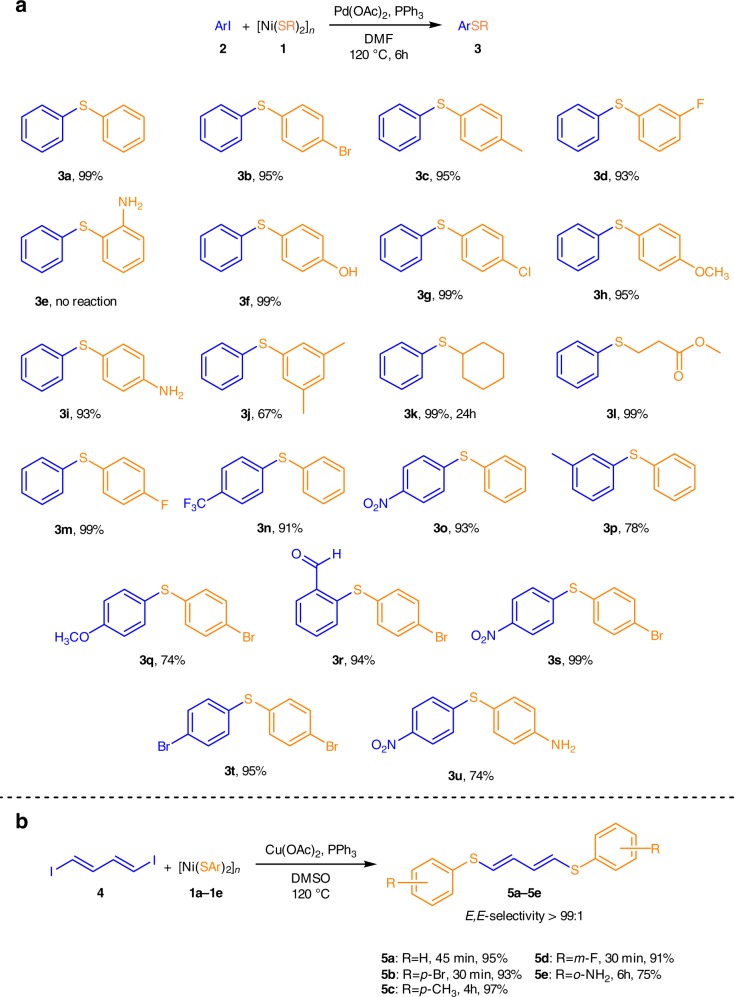
C–S cross-coupling reactions with nickel thiolates. Palladium-catalyzed C–S bond formation reaction between various aryl iodides and nickel thiolates (**a**). Copper-catalyzed C–S cross-coupling with (*E*,*E*)−1,4-diiodobuta-1,3-diene (**b**). See Supplementary Methods for experimental details

To address synthetic applications, we carried out the cross-coupling reaction between various aryl halides and nickel thiolates (Fig. 9a). The C–S bond formation reaction between iodobenzene and thiolates **1a**–**1d** and **1f**–**1m** proceeded smoothly. Substituted diaryl sulfides and alkyl aryl sulfides **3** were formed in 65–99% yields (see Fig. 9a and Supplementary Methods for experimental details). Thus, both thiolates with aromatic and aliphatic substituents can be successfully used as a source of SR groups for the cross-coupling reaction. The influence of the unprotected remote *p*-OH and *p*-NH_2_ groups in thiolates on the reaction outcome was negligible; diaryl sulfides **3f** and **3i** were formed with 99 and 93% yields, respectively. The reaction of nickel thiolates with various aryl iodides resulted in the formation of the desired diaryl sulfides for the wide range of substituents in the aromatic rings of the organic halides. The yields of products **3n**–**3u** were 74–99%, depending on the nature of the substituent. A lower reactivity was observed in the case of electron-donating groups in the aryl halides, and a higher reactivity – in the case of electron-withdrawing groups (Fig. 9a). Interestingly, thiolate **1e** demonstrated no reactivity in the cross-coupling, probably because of the strong chelating binding of the SAr and NH_2_ groups to the Ni atom confirmed by XAS measurements (Fig. 3) and density functional theory (DFT) calculations (see Supplementary Discussion for results of DFT studies). Aryl bromides and chlorides were successfully utilized in the reaction using a very simple Pd(OAc)_2_/PPh_3_ catalytic system (see Supplementary Table 1).

The studied scope was not limited to aryl halides, and the highly sensitive substrate (*E*,*E*)−1,4-diiodobuta-1,3-diene was utilized as a reagent. Its practical relevance is clearly demonstrated in the preparation of conjugated sulfur-substituted dienes with unprecedented selectivity. Challenging butadiene derivatives **5a**–**5e** were isolated in 75–97% yields (Fig. 9b). Remarkably, such a sensitive parameter as the geometry of both double bonds was completely preserved in **5a**–**5e** (Fig. 9b). Importantly, in this case a copper catalyst was successfully used instead of a palladium-based catalytic system.

### Mechanistic features of C–S coupling with nickel thiolates

**Fig. 10:**
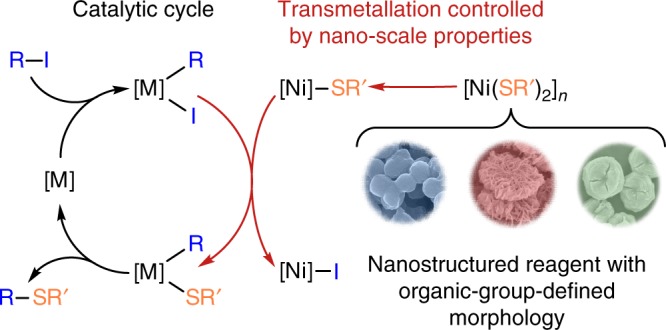
Proposed mechanism of the coupling reaction with nickel thiolates. Schematic representation of mechanism of catalytic C–S cross-coupling reaction with nano-structured nickel thiolates (M = Pd or Cu)

To highlight the concept proposed in the present study, the fundamental mechanistic difference between the standard well-known catalytic cycle and the nano-scale-defined catalytic cycle developed herein (Fig. 10) should be emphasized. The catalytic cycle of the standard C–S cross-coupling reaction involves oxidative addition of organic halide, followed by transmetallation and C–S reductive elimination^43–45^. Typically, in order to influence the reaction and to gain control over the process in such a case, one needs to introduce substituents with specific electron or/and steric properties into the substrates or into the ligands coordinated to the metal center. In the developed system, the use of nanostructured reagents allows to control the process and substrate properties at the nanoscale level without the need of modification of the molecular structure of the reacting moieties (Fig. 10). Transmetallation involving a nano-structured reagent was successfully incorporated into a well-known catalytic system (Fig. 10). Very good performance for a variety of substrates was demonstrated with rather simple Pd(OAc)_2_/PPh_3_ and Cu(OAc)_2_/PPh_3_ catalytic systems. Thus, nano-structured reagents provide an additional level of the reaction control within the practically valuable catalytic process.

Overall, real-time FE-SEM observation in the liquid phase revealed the mechanism of the C–S cross-coupling reaction at the nanoscale. Nickel particles with unique morphology and nanostructured surface were synthesized and successfully used as a source of reactive groups in the cross-coupling reaction. With the use of advanced liquid phase electron microscopy, evolution of metal particles during the reaction was captured on FE-SEM images and video. Mass spectrometry experiments allowed detecting the key intermediates of oxidative addition and transmetallation processes in solution. Solid-phase electron microscopy studies and kinetic measurements demonstrated that the variation in the substituents of the organic group results in crucial change of thiolates activity and their nanoscale morphology and provide the rationalization to organic-group-defined nanoscale reagents. The nanoscale modulation of reactants will open wide opportunities for optimization of synthetic processes. The application domain of our findings is apparently beyond the studied cross-coupling reaction and can be readily extended to several other catalytic reactions with nanostructured organization of the reactants.

The possibility of recording a video of a chemical transformation in liquid phase has been desperately awaited by many chemists. However, the prospects of direct recording are slim due to the notorious limitations of electron microscopy of liquid samples. In this article, we present a detailed real-time video record of the catalytic cross-coupling reaction at the nanoscale level. The approach and findings can be extrapolated to similar yet unstudied systems and are likely to form the basis of a promising direction in nano-science and chemical research.

## Methods

### General considerations

All starting materials, catalysts and solvents were purchased from the commercial sources.

NMR data were recorded using Bruker Fourier-300 (working frequency 300.1 MHz for ^1^H), Bruker Avance-400 (400.1 MHz for ^1^H), Bruker DRX-500 (500.1 MHz for ^1^H) and Bruker Avance-600 (600.1 MHz for ^1^H) NMR spectrometers. Residual solvent signal was used as a chemical shift reference.

The solid-state scanning electron microscopy measurements were performed with the use of Hitachi SU8000 field-emission scanning electron microscope (FE-SEM) operating in secondary electron mode at 10 kV accelerating voltage. Before the measurements powdered samples supported on aluminum foil were fixed on 25 mm aluminum specimen stub by conductive silver glue followed by the coating with 7 nm of gold/palladium alloy (60:40) with the use of magnetron sputter coater. The values of the magnification given on the figures were measured in relation to the standard 1280 × 960 frame with 256 dpi resolution. EDX-SEM studies were carried out using Oxford Instruments X-max EDX system at 20 kV accelerating voltage. Before the measurements all samples were fixed on the surface of conductive carbon tape and coated with a thin film (15 nm) of carbon.

Detailed description of the all synthetic procedures is available in the Supplementary Methods.

### SEM observations of 1b disassembling in liquid

Five milligram of **1b** were dispersed in 1 mL of isopropanol. Ten microliter of the resulting suspension were placed into the sample dish of a Quantomix^TM^ QX-102 liquid SEM capsule. The solvent was removed by air flow. Then, the sample dish was filled with 15 μL of the working solution prepared by dissolution of Pd(OAc)_2_ (4.5 mg, 0.02 mmol) and dppe (16 mg, 0.04 mmol) in 1 mL of EtOH. After that, the capsule was sealed and transferred to the electron microscope chamber. The observations were performed using a Hitachi SU8000 field-emission scanning electron microscope (FE-SEM). Electron microscopy data were acquired in backscattered electron mode (with compositional contrast) at 30 kV accelerating voltage. For observations of reference samples, pure alcohol (isopropanol or ethanol) was used as a working solution. In the case of all liquid SEM measurements, the use of relatively small accelerating voltage of 30 kV (for sample analysis inside capsules) and very low probe current (typical for cold field emission instrument) allowed minimizing the influence of electron beam on sensitive samples.

### SEM observations of PhI-assisted 1b disassembling in liquid

Five milligram of **1b** were dispersed in 1 mL of isopropanol. 10 μL of the resulting suspension were placed into the sample dish of a Quantomix^TM^ QX-102 liquid SEM capsule. The solvent was removed by air flow. Then, the sample dish was filled with 15 μL of the working solution prepared by dissolution of Pd(OAc)_2_ (4.5 mg, 0.02 mmol), dppe (16 mg, 0.04 mmol), and PhI (45 μL, 82 mg, 0.4 mmol) in 1 mL of EtOH. After that, the capsule was sealed and transferred to the electron microscope chamber. The observations were performed using a Hitachi SU8000 field-emission scanning electron microscope (FE-SEM). Electron microscopy data were acquired in backscattered electron mode (with compositional contrast) at 30 kV accelerating voltage.

### FE-SEM video recording

Scanning electron microscopy videos were recorded with the use of Epiphan DVI2USB3.0 frame grabber. The video capture device was connected to the workstation of electron microscope and the video stream was captured and saved on a laptop with the use of Epiphan Frame Grabber software (v. 3.29.1.0). The slow scanning mode (2 frames/s) of electron microscope was used for video recording.

### FE-SEM monitoring of reaction of 1b with iodobenzene

4.5 mg (0.02 mmol) of Pd(OAc)_2_, 21 mg (0.08 mmol) of PPh_3_ and **1b** (85.8 mg, 0.2 mmol) were placed in a 5 mL test-tube equipped with a magnetic stir bar. Then, 1 mL of DMF and 0.045 mL (82 mg, 0.4 mmol) of iodobenzene were added to the test-tube. The reaction mixture was stirred at 120 °C. Samples of the reaction mixture were taken after 45 min and 2 h from the beginning of the reaction and were diluted with isopropanol (1 mL of isopropanol per 50 μL of the reaction mixture). 15 μL of the diluted sample was placed in a Quantomix^TM^ QX-102 sample dish. After that, the capsule was sealed and transferred to the electron microscope chamber. The observations were performed using a Hitachi SU8000 field-emission scanning electron microscope (FE-SEM). Images were acquired in backscattered electron mode (with compositional contrast) at 30 kV accelerating voltage.

### Mass spectrometry studies

Mass spectra were measured using Bruker maXis instrument equipped with an electrospray ionization source and spectra were recorded with *m/z* 50–1500 range. Capillary Voltage was set: for the positive ion mode to –4.5 kV, and for the negative ion mode to + 4.0 kV. For both modes applied Spray Shield Offset was set to –0.5 kV. For calibration of the mass spectra a low-concentration tuning mix solution by Agilent Technologies was utilized. Nitrogen was applied as a nebulizer gas (0.4 bar) and dry gas (4.0 L × min^−1^, 250 °C). All the MS spectra were recorded at 2 Hz (positive mode) or 4 Hz (negative mode) frequency. Spectra were processed after background subtraction. Bruker Data Analysis 4.0 software package was used.

### Online ESI-MS monitoring of coupling reaction in (–)MS mode

A 25 mL flask for monitoring (one neck and one condenser pointed with a needle-valve) equipped with a magnetic stir bar was loaded with 4-iodobenzenesulfonic acid (**2b**, 1.0 mg, 3.5 µmol) and thiolate **1b** (0.8 mg, 1.75 µmol), and then was thoroughly flushed with Ar. After that, degassed DMF (10 mL) was charged into the flask under argon backflush, and the neck was closed with a silicon septum. A red PEEK capillary (72 cm) was pulled into the flask through the septum and was immersed into the reaction mixture. The flask was placed into a hot oil bath, and then was heated at 120 °C under continuous magnetic stirring. After heating for ~1 min, a degassed 1.18 × 10^–2^ M solution of PPh_3_ (60 µL) was injected into the flask via a syringe through the septum. After heating for ~2 min, a degassed 4.45 × 10^–3^ M solution of Pd(OAc)_2_ (40 µL) was injected into the flask via a syringe through the septum. The reaction was monitored for 10 min (2 trials). Reactant, product, and intermediates were monitored as signals of [M – H]^-^ ions.

### Online ESI-MS monitoring of coupling reaction in (+)MS mode

A 25 mL flask for monitoring (one neck and one condenser pointed with a needle-valve) equipped with a magnetic stir bar was loaded with thiolate **1i** (0.5 mg, 1.75 µmol), and was thoroughly flushed with Ar. Then degassed DMF (10 mL) and iodobenzene (0.7 mg, 3.5 µmol) were charged into the flask under argon backflush, and the neck was closed with a silicon septum. A red PEEK capillary (72 cm) was pulled into the flask through the septum and was immersed into the reaction mixture. The flask was placed into a hot oil bath, and then was heated at 120 °C under continuous magnetic stirring. After heating for ~1 min, a degassed 1.18 × 10^–2^ M solution of PPh_3_ (60 µL) was injected into the flask via a syringe through the septum. After heating for ~2 min, a degassed 4.45 × 10^–3^ M solution of Pd(OAc)_2_ (40 µL) was injected into the flask via a syringe through the septum. The reaction was monitored for 45 min (2 trials). Reactant, product, and intermediates were monitored as signals of [M − I]^+^ or [M + H]^+^ ions.

### Kinetic measurements

Palladium acetate (4.5 mg, 0.02 mmol), PPh_3_ (21 mg, 0.08 mmol) and corresponding nickel thiolate **1** (0.2 mmol) were placed in a 5 mL test-tube equipped with a magnetic stir bar. Then, 1 mL of DMF and 0.045 mL (82 mg, 0.4 mmol) of iodobenzene were added to the tube. The reaction vessel was flushed with argon and sealed with a screw cap. The reaction mixture was stirred at 120 °C. 20 µL samples of the reaction mixture were taken at specified time intervals. Each sample was diluted with 600 µL of CH_2_Cl_2_. The liquid phase was separated by centrifugation and analyzed by using a SCION 436-GC gas chromatograph with a flame ionization detector. Product yields were calculated on the basis of the normalization of the sum of iodobenzene and all the compounds derived from iodobenzene to 100%. The reliability of quantitative data was estimated by independent ^1^H NMR analysis of selected samples.

### X-ray spectroscopy

Experiments were carried out on beamline ID26 of the European Synchrotron Radiation Facility (ESRF). The incident energy was selected by the (111) reflection of a pair of cryogenically cooled Si crystals. Harmonic rejection was achieved by three Si mirrors working at 2.5 mrad in total reflection. The Ni Kβ-lines were recoded using the (551) reflection of five spherically bent (R = 1 m) Si crystals arranged with an Avalanche photodiode in Rowland geometry. High energy resolution fluorescence detected X-ray absorption spectra (HERFD-XAS) were obtained by tuning a fluorescence spectrometer to the maximum of the Kβ_1,3_ line and scanning the incoming energy. The beam footprint on the sample was 0.1 × 1 mm^2^ (vertical × horizontal). The samples were cooled to 10 K in a He cryostat. Before the measurements, the samples were mixed with microcrystalline cellulose (10% w/w of sample) and pressed to pellets. The spectra were processed using PyMCA 5.1.1 and Demeter 0.9.24 software packages.

### Data availability

All data supporting the findings of the current study are available in the article and its Supplementary Information. Additional relevant data are available from the corresponding author upon reasonable request.

## Electronic supplementary material

Supplementary Information

Descriptions of Additional Supplementary Files

Supplementary Movie 1

Supplementary Movie 2
