# Supplementary material for: Exploring the performance of nanostructured reagents with organic-group-defined morphology in cross-coupling reaction
Source: Nat Commun. 2018 Jul 26;9:2936. doi: 10.1038/s41467-018-05350-x (PMC6062554; doi:10.1038/s41467-018-05350-x)
Supplement: Supplementary file 1 — Supplementary Information [file 41467_2018_5350_MOESM1_ESM.pdf]

Supplementary Information

Exploring the Performance of Nanostructured Reagents with Organic-group-defined Morphology in Cross-Coupling Reaction

Alexey S. Kashin, Evgeniya S. Degtyareva, Dmitry B. Eremin, and Valentine P. Ananikov*

Zelinsky Institute of Organic Chemistry, Russian Academy of Sciences, Leninsky Prospect, 47, Moscow 119991, Russia; E-mail: val@ioc.ac.ru; http://AnanikovLab.ru

Table of Contents

Supplementary Methods 3
Supplementary Figures 7
Supplementary Tables 47
Supplementary Discussion 48
Supplementary References 48

Supplementary Methods

Synthesis of nickel thiolates 1a-1e. 385.4 mg (1.5 mmol) of nickel acetylacetonate were dissolved in 6 mL of methylene chloride (or in the mixture of 2 mL of CH_2Cl_2 and 6mL of acetone in the case of **1c**). Then, 30 mmol of thiol (as a neat liquid, or saturated solution in CH_2Cl_2, or saturated solution in acetone for **1c**) were added to the solution. Reaction mixture was stirred at room temperature for 10 minutes until complete precipitation of the product. The precipitate was separated by centrifugation, washed with acetone (5 x 10 mL) and dried under reduced pressure (20 mbar).

[Ni(SPh)$_2$]$_n$ (1a): Dark violet powder. Yield: 415 mg (99%). Elemental analysis: found (%): C – 51.75, H – 3.79, Ni – 21.01, S – 22.70; calculated for $C_{12}H_{10}NiS_2$ (%): C – 52.03, H – 3.64, Ni – 21.19, S – 23.15.

[Ni(S*p*-BrC$_6$H$_4$)$_2$]$_n$ (1b): Black powder. Yield: 609 mg (93%). Elemental analysis: found (%): C – 32.71, H – 1.90, Br – 35.48, Ni – 14.45, S – 14.23; calculated for $C_{12}H_8Br_2NiS_2$ (%): C – 33.15, H – 1.85, Br – 36.75, Ni – 13.50, S – 14.75.

[Ni(S*p*-CH$_3$C$_6$H$_4$)$_2$]$_n$ (1c): Dark brown powder. Yield: 225 mg (50%). Elemental analysis: found (%): C – 54.66, H – 4.57, Ni – 19.40, S – 19.71.; calculated for $C_{14}H_{14}NiS_2$ (%): C – 55.12, H – 4.63, Ni – 19.24, S –21.02.

[Ni(S*m*-FC$_6$H$_4$)$_2$]$_n$ (1d): Brown powder. Yield: 291 mg (62%). Elemental analysis: found (%): C – 45.88, H – 2.52; calculated for $C_{12}H_8F_2NiS_2$ (%): C – 46.05, H – 2.58.

[Ni(S*o*-NH$_2$C$_6$H$_4$)$_2$]$_n$ (1e): Yellow-green powder. Yield: 431 mg (93%). Elemental analysis: found (%): C – 47.21, H – 3.90, N – 9.24, Ni – 18.90, S – 20.63; calculated for $C_{12}H_{12}N_2NiS_2$ (%): C – 46.94, H – 3.94, N – 9.12, Ni – 19.11, S – 20.88.

Synthesis of nickel thiolates 1f-1m. 385.4 mg (1.5 mmol) of nickel acetylacetonate were dissolved in 6 mL of methylene chloride (or in the mixture of 2 mL of CH_2Cl_2 and 4 mL of acetone in the case of **1h**, or in the mixture of 2 mL of CH_2Cl_2 and 4mL of toluene in the case of **1k** and **1l**). Then, 7.5 mmol of thiol (as a neat liquid or a saturated solution in CH_2Cl_2 for solid thiols) were added to the solution. Reaction mixture was kept at room temperature until complete precipitation of the product (from 30 minutes for **1f-1g** to 3 days for **1k-1l**). The precipitate was separated by centrifugation, washed with acetone (5 x 10 mL) (or toluene in the case of **1k-1l**) and then dried in the open air.

[Ni(S*p*-OHC$_6$H$_4$)$_2$]$_n$ (1f): Dark brown powder. Yield: 405 mg (87%).

[Ni(S*p*-ClC$_6$H$_4$)$_2$]$_n$ (1g): Brown powder. Yield: 214 mg (41%).

[Ni(S*p*-OCH$_3$C$_6$H$_4$)$_2$]$_n$ (1h): Black powder. Yield: 310 mg (61%).

[Ni(S*p*-NH$_2$C$_6$H$_4$)$_2$]$_n$ (1i): Dark brown powder. Yield: 460 mg (99%).

[Ni(S*m*-(CH$_3$)$_2$C$_6$H$_3$)$_2$]$_n$ (1j): Dark brown powder. Yield: 312 mg (63%).

[Ni(SCy)$_2$]$_n$ (1k): Dark brown powder. Yield: 365 mg (84%).

[Ni(SCH$_2$CH$_2$COOCH$_3$)$_2$]$_n$ (1l): Dark brown powder. Yield: 45 mg (10%).

[Ni(S*p*-FC$_6$H$_4$)$_2$]$_n$ (1m): Brown powder. Yield: 435 mg (93%).

Synthesis of nickel thiolates 1a', 1b', 1g'. 373.3 mg (1.5 mmol) of nickel acetate were dissolved in 6 mL of methanol. Then, 7.5 mmol of thiol (as a neat liquid for **1a'** or a saturated solution in CH_2Cl_2 for **1b'** and **1g'**) were added to the solution. Reaction mixture was kept at room

temperature until complete precipitation of the product (for 30 minutes). The precipitate was separated by centrifugation, washed with acetone (5 x 10 mL) and then dried in the open air.

$[Ni(SPh)_2]_n$ (1a'): Dark brown powder. Yield: 383 mg (92%).

$[Ni(S$*p*$-BrC_6H_4)_2]_n$ (1b'): Dark brown powder. Yield: 370 mg (57%).

$Ni(S$*p*$-ClC_6H_4)_2]_n$ (1g'): Dark brown powder. Yield: 412 mg (79%).

Synthesis of nickel thiolate 1a''. 385.4 mg (1.5 mmol) of nickel acetylacetonate and 3.93 g (15 mmol) of PPh_3 were dissolved in 25 mL of acetonitrile. Then, saturated solution of 3.28 g (15 mmol) of diphenyldisulfide in acetonitrile was added to the reaction mixture. Resulting solution was kept at room temperature until complete precipitation of the product (for 30 minutes). The precipitate was separated by centrifugation, washed with acetone (10 x 10 mL) and then dried in the open air.

$[Ni(SPh)_2]_n$ (1a''): Dark brown powder. Yield: 180 mg (43%).

Reaction of 1b with iodobenzene in the presence of PPh_3. Palladium acetate (4.5 mg, 0.02 mmol), PPh_3 (21 mg, 0.08 mmol) and **1b** (87 mg, 0.2 mmol) were placed in a 5-mL test-tube equipped with a magnetic stirrer bar. Then, 1 mL of DMF and 0.045 mL (82 mg, 0.4 mmol) of iodobenzene were added to the test-tube. The reaction vessel was flushed with argon and sealed with a screw cap. The reaction mixture was stirred at 120°C for 6h. After completion of the reaction the liquid phase was separated and analyzed by ^1H NMR spectroscopy. NMR yield of the coupling product was 95%.

Reaction of 1b with iodobenzene in the presence of bis(diphenylphosphino)ethane (dppe). Palladium acetate (4.5 mg, 0.02 mmol), dppe (16 mg, 0.04 mmol) and **1b** (87 mg, 0.2 mmol) were placed in a 5-mL test-tube equipped with a magnetic stirrer bar. Then, 1 mL of ethanol and 0.045 mL (82 mg, 0.4 mmol) of iodobenzene were added to the test-tube. The reaction vessel was flushed with argon and sealed with a screw cap. The reaction mixture was stirred at 120°C for 4h. After completion of the reaction the liquid phase was separated and analyzed by ^1H NMR spectroscopy. NMR yield of the coupling product was 35%.

Pd-catalyzed cross-coupling between nickel thiolates and aryl iodides. Palladium acetate (4.5 mg, 0.02 mmol), PPh_3 (21 mg, 0.08 mmol), nickel thiolate (0.2 mmol) and aryl iodide (0.4 mmol, in the case of solid aryl iodides) were placed in a 5-mL test-tube equipped with a magnetic stirrer bar. Then, 1 mL of DMF and 0.4 mmol of aryl iodide (in the case of liquid aryl iodides) were added to the test-tube. The reaction vessel was flushed with argon and sealed with a screw cap. The reaction mixture was stirred at 120°C for 6h (or 24h in the case of thiolate **1k**). After completion of the reaction the liquid phase was separated and diluted with 5 mL of diethyl ether. Resulting suspension was washed with 10 mL of water. Liquid organic phase was separated, solvent was removed on rotary evaporator and the residue was analyzed by ^1H NMR spectroscopy. The products **3a-3u** were identified according to the published data [1-8]. Obtained crude products containing minor (or trace) amounts of unreacted aryl halide and phosphine can be further purified by column chromatography using procedure described earlier [9].

Pd-catalyzed cross-coupling between nickel thiolates and aryl bromides/chlorides. Palladium acetate (4.5 mg, 0.02 mmol), PPh_3 (21 mg, 0.08 mmol), nickel thiolate (0.2 mmol) and aryl halide (0.4 mmol) were placed in a 5-mL test-tube equipped with a magnetic stirrer bar. Then,

1 mL of DMF was added to the test-tube. The reaction vessel was flushed with argon and sealed with a screw cap. The reaction mixture was stirred at 120°C for 24h or 72h (see Supplementary Table 1 for the reaction times). After completion of the reaction the liquid phase was separated and diluted with 5 mL of diethyl ether. Resulting suspension was washed with 10 mL of water. Liquid organic phase was separated, solvent was removed on rotary evaporator and the residue was analyzed by ^{1}H NMR spectroscopy. The products were identified according to the published data [10-12]. Obtained crude products containing minor (or trace) amounts of unreacted aryl halide and phosphine can be further purified by column chromatography using procedure described earlier [9].

Synthesis of (*E*,*E*)-1,4-diiodobuta-1,3-diene (4). The synthesis was performed according to the published procedure [13]. Platinum (IV) chloride (0.0118 g, 0.035 mmol), sodium iodide (0.6 g, 4 mmol) and iodine (1.015 g, 4 mmol) were placed in a 20-mL test-tube equipped with a magnetic stirrer bar. Then, 5 mL of saturated solution of acetylene in acetone were added to the test-tube and the reaction vessel was immediately sealed with a screw cap. The reaction mixture was stirred at room temperature for 20 hours. After completion of the reaction, 30 mL of distillated water were added to the reaction mixture resulting in the product precipitation. The formed solid was separated by centrifugation and then washed with water (1x5 mL), saturated aqueous NaI solution (2x5 mL) and again with water (2x5 mL). Obtained crude product was dried in the open air. Purification of the product by sublimation in vacuum (30-35 °C, 0.06-0.07 mbar) yielded high-purity (99+%) compound.

Yellow crystalline solid. Yield: 0.2 g. NMR ^{1}H (500 MHz, acetone-d6, δ): 7.08-7.16 (m, 2H), 6.74-6.82 (m, 2H). NMR ^{13}C{^{1}H} (125 MHz, acetone-d6, δ): 145.9 (s), 82.3 (s).

Synthesis of dienes 5a-5d. Copper acetate (5.4 mg, 0.03 mmol), PPh_3 (31 mg, 0.12 mmol), corresponding nickel thiolate (0.33 mmol) and (*E*,*E*)-1,4-diiodobuta-1,3-diene (92 mg, 0.3 mmol) were placed in a 20-mL test tube equipped with a magnetic stirrer bar. Then, 1.5 mL of DMSO were added to the test-tube. The reaction vessel was flushed with argon and sealed with a screw cap. The reaction mixture was stirred at 120°C until complete thiolate dissolution. After completion of the reaction, reaction mixture was diluted with ether (50 mL), filtered through the Celite pad, washed with water (3x50 mL) and brine (50 mL). The ether fraction was dried over anhydrous sodium sulfate. Solvent was removed under the reduced pressure. The obtained residue was dissolved in hexanes and filtered through the column with aluminum oxide. Described purification method gives the product with 90-95% purity.

(*E*,*E*)-1,4-bis(phenylthio)buta-1,3-diene (5a): White crystalline solid. Yield: 77 mg (95%). NMR ^{1}H (600 MHz, acetone-d_6, δ): 7.33-7.42 (m, 8H), 7.25-7.32 (m, 2H), 6.48-6.60 (m, 4H). NMR ^{13}C{^{1}H} (150 MHz, acetone-d_6, δ): 135.8 (s), 131.6 (s), 130.2 (s), 130.1 (s) , 127.8 (s), 126.3 (s).

(*E*,*E*)-1,4-bis(4-bromophenylthio)buta-1,3-diene (5b): White crystalline solid. Yield: 118 mg (93%). NMR ^{1}H (600 MHz, acetone-d_6, δ): 7.52-7.57 (m, 4H), 7.31-7.36 (m, 4H), 6.52-6.63 (m, 4H). ^{13}C{^{1}H} (150 MHz, acetone-d_6, δ): 135.5 (s), 133.2 (s), 132.3 (s), 131.8 (s), 125.9 (s), 121.2 (s).

(*E*,*E*)-1,4-bis(4-methylphenylthio)buta-1,3-diene (5c): White crystalline solid. Yield: 86 mg (97%). NMR ^{1}H (600 MHz, acetone-d_6, δ): 7.24-7.31 (m, 4H), 7.17-7.23 (m, 4H), 6.38-6.51 (m, 4H), 2.32 (s, 6H). ^{13}C{^{1}H} (150 MHz, acetone-d_6, δ): 138.0 (s), 131.9 (s), 130.9 (s), 130.8 (s), 130.7 (s), 126.8 (s), 21.0 (s).

(*E*,*E*)-1,4-bis(3-fluorophenylthio)buta-1,3-diene (5d): White crystalline solid. Yield: 85 mg (91%). NMR ^{1}H (600 MHz, acetone-d_6, δ): 7.37-7.47 (m, 2H), 7.19-7.23 (m, 2H), 7.13-7.19 (m, 2H),

7.01-7.07 (m, 2H), 6.61-6.70 (m, 4H). $^{13}C\{^{1}H\}$ (150 MHz, acetone-d_6, δ): 163.8 (d, 247.1 Hz), 138.7 (d, 7.7 Hz), 132.9 (s), 131.8 (d, 8.6 Hz), 125.6 (s), 125.3 (br. s), 116.0 (d, 23.4 Hz), 144.3 (d, 21.7 Hz).

Synthesis of (*E*,*E*)-1,4-bis(2-aminophenylthio)buta-1,3-diene (5e) dihydrochloride. Copper acetate (5.4 mg, 0.03 mmol), PPh_3 (31 mg, 0.12 mmol), $[Ni(S\textit{o}\text{-}NH_2C_6H_4)_2]_n$ (154 mg, 0.5 mmol) and (*E*,*E*)-1,4-diiodobuta-1,3-diene (92 mg, 0.3 mmol) were placed in a 20-mL test tube equipped with a magnetic stirrer bar. Then, 1.5 mL of DMSO were added to the test-tube. The reaction vessel was flushed with argon and sealed with a screw cap. The reaction mixture was stirred at 120°C until complete thiolate dissolution. After completion of the reaction, reaction mixture was diluted with ether (50 mL), filtered through the Celite pad, washed with water (3x50 mL) and brine (50 mL). The ether fraction was dried over anhydrous sodium sulfate. Solvent was removed under the reduced pressure. The obtained residue was treated by 10 mL of the HCl saturated solution in ether. The formed precipitate was washed with ether (3x15 mL) and dried.

Beige crystalline solid. Yield: 84 mg (75%). NMR ^{1}H (400 MHz, DMSO-d_6, δ): 8.02 (br. s, 6H), 7.38-7.45 (m, 2H), 7.26-7.38 (m, 4H), 7.07-7.18 (m, 2H), 6.28-6.47 (m, 4H). $^{13}C\{^{1}H\}$ (101 MHz, DMSO-d_6, δ): 137.81 (s), 133.54 (s), 129.96 (s), 129.40 (s), 125.05 (s), 124.82 (s), 123.04 (s), 121.15 (s).

Supplementary Figures

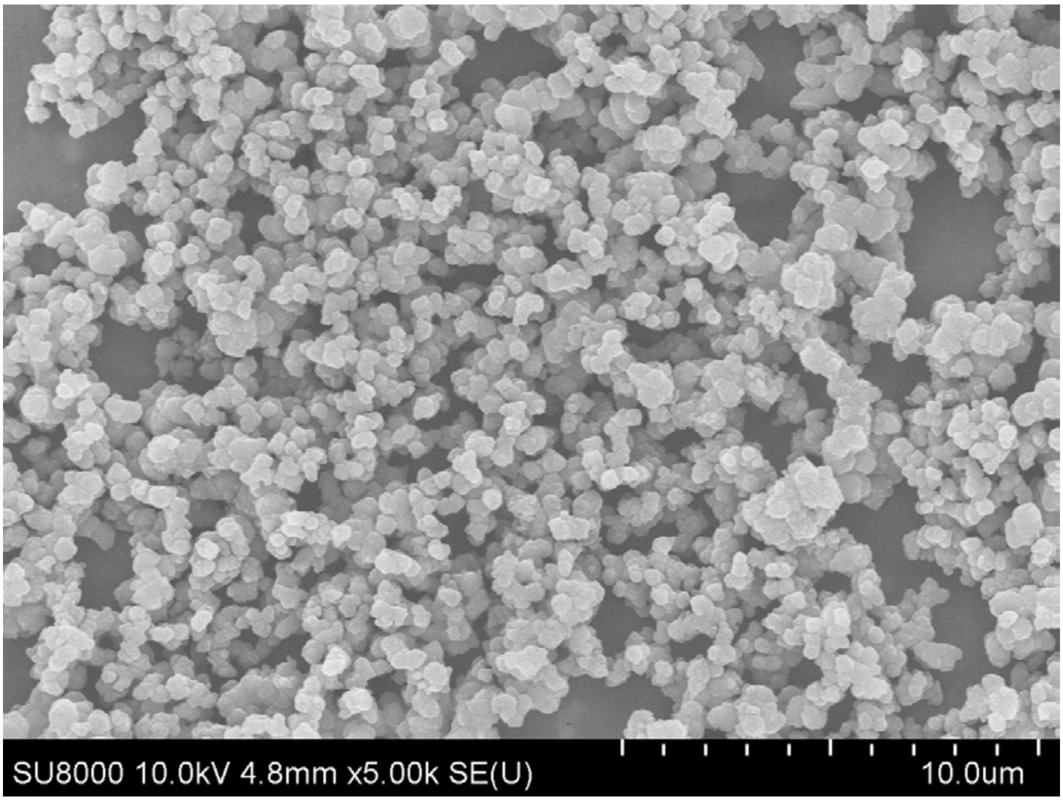

Supplementary Figure 1. FE-SEM image of $[Ni(SPh)_2]_n$ (**1a**) at x5000 magnification.

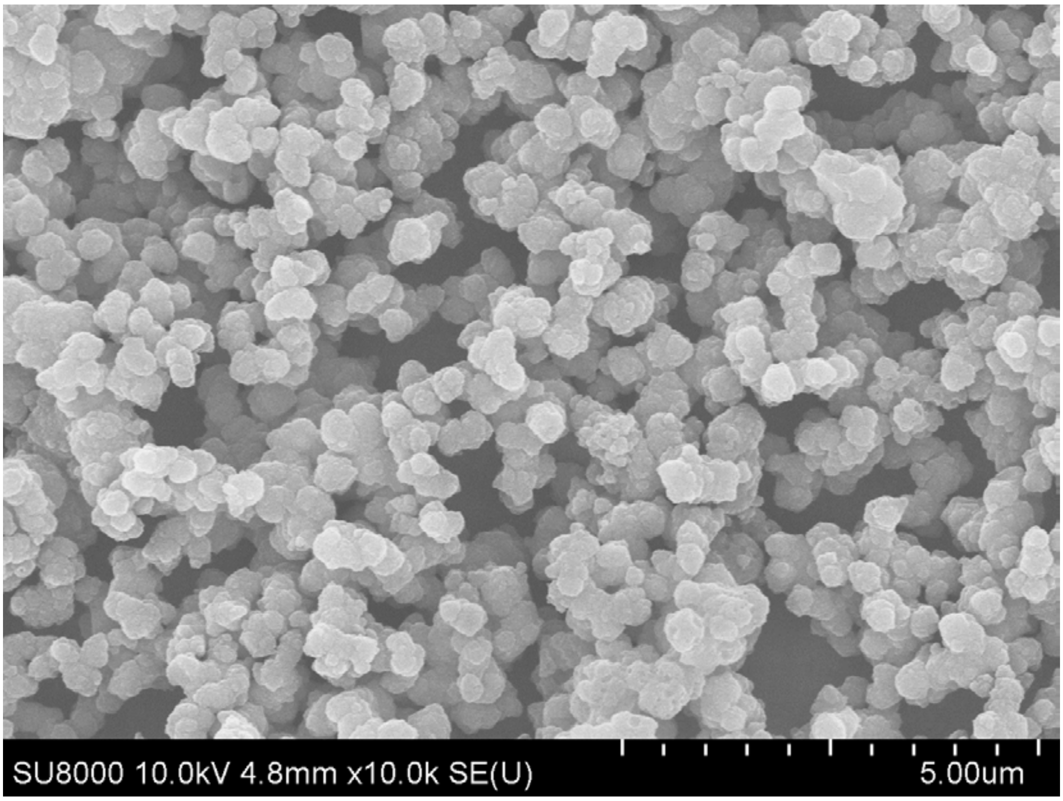

Supplementary Figure 2. FE-SEM image of $[Ni(SPh)_2]_n$ (**1a**) at x10000 magnification.

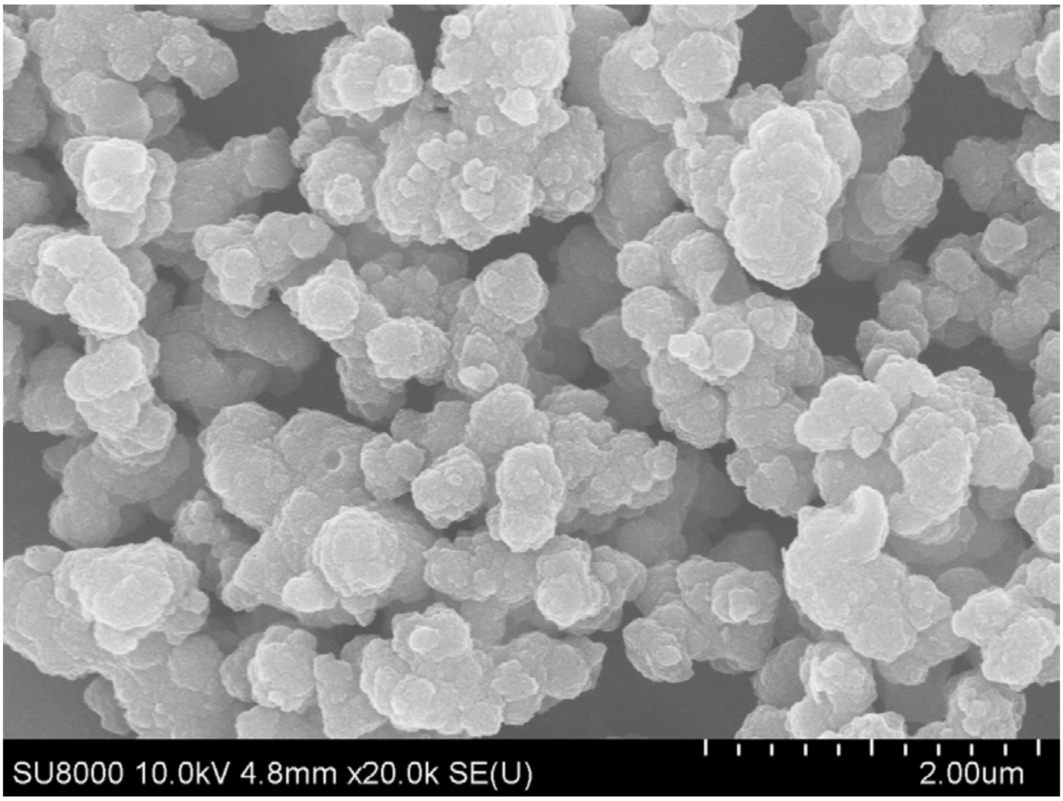

Supplementary Figure 3. FE-SEM image of $[Ni(SPh)_2]_n$ (**1a**) at x20000 magnification.

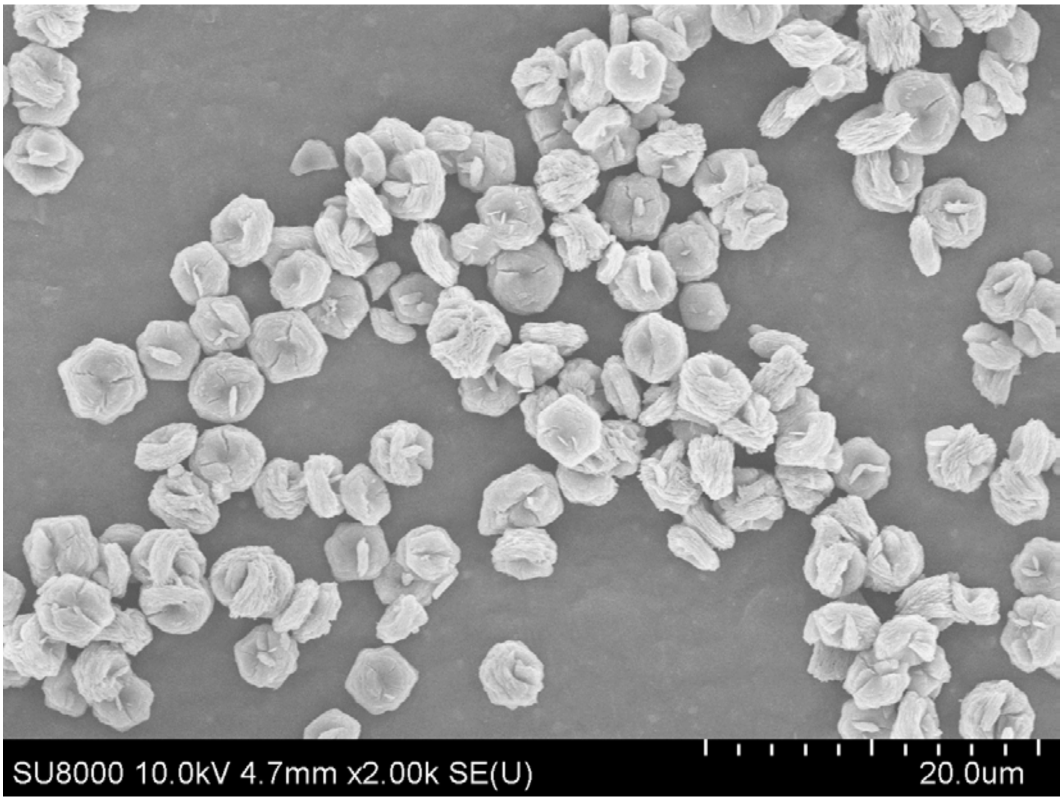

Supplementary Figure 4. FE-SEM image of $[Ni(S\textit{p}\text{-}BrC_6H_4)_2]_n$ (**1b**) at x2000 magnification.

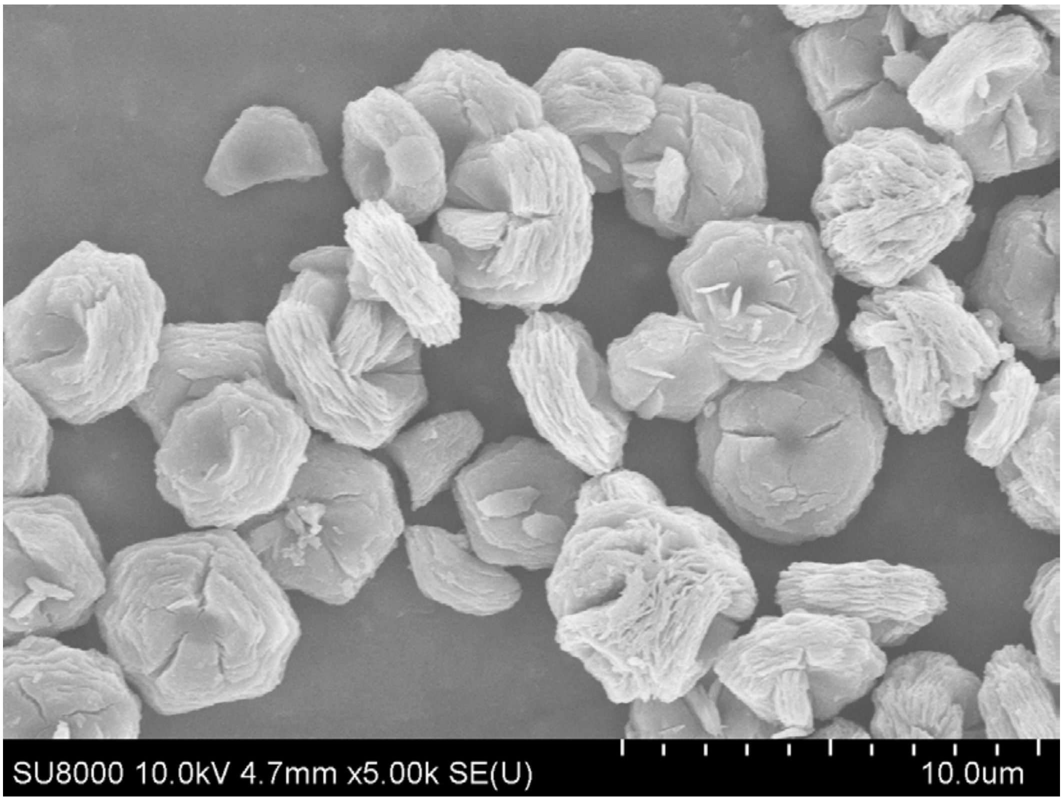

Supplementary Figure 5. FE-SEM image of $[Ni(S\textit{p}\text{-}BrC_6H_4)_2]_n$ (**1b**) at x5000 magnification.

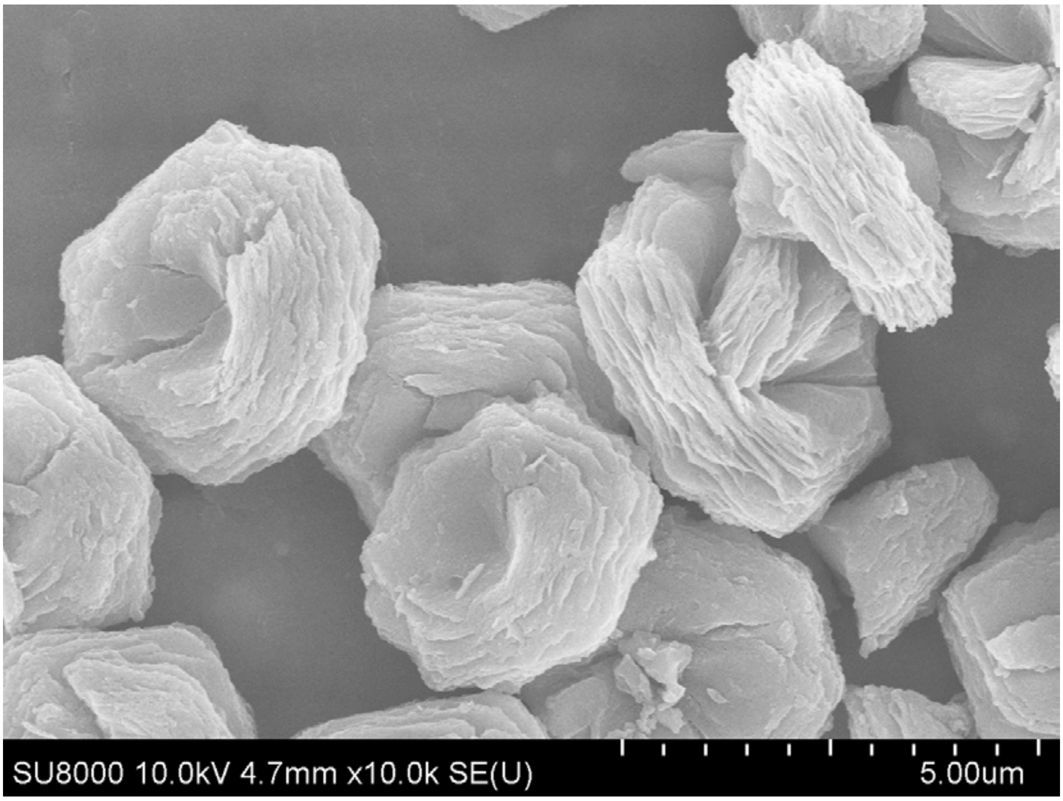

Supplementary Figure 6. FE-SEM image of $[Ni(S\textit{p}\text{-}BrC_6H_4)_2]_n$ (**1b**) at x10000 magnification.

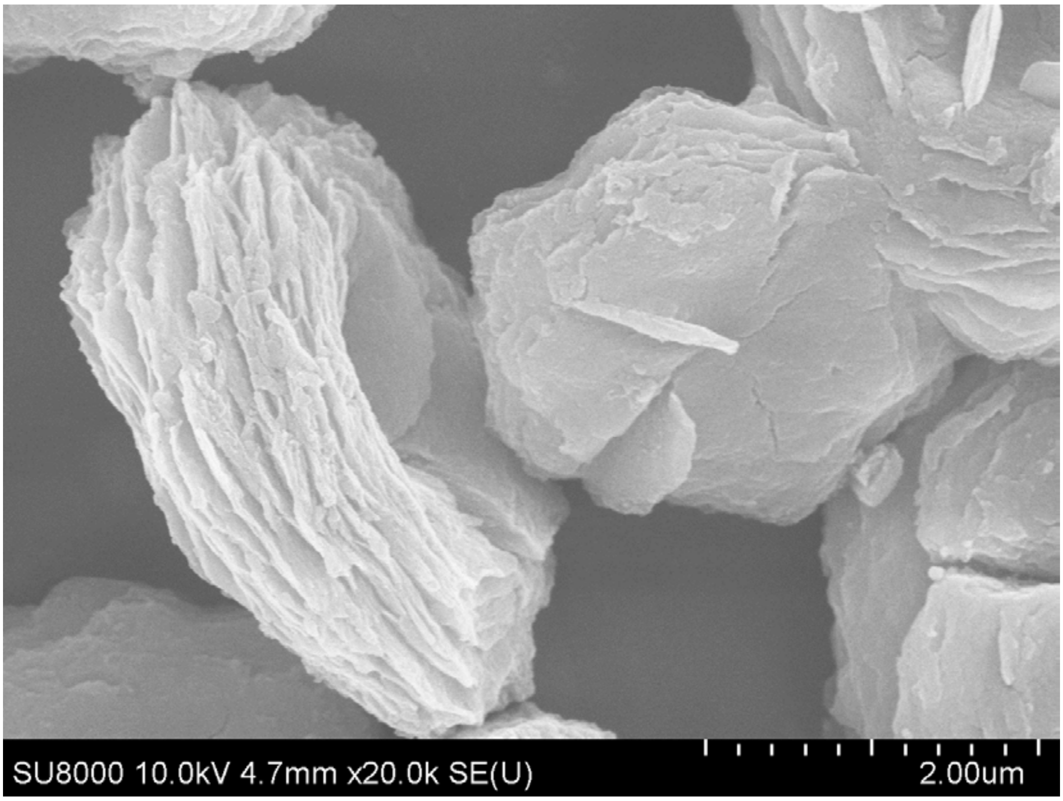

Supplementary Figure 7. FE-SEM image of $[Ni(Sp\text{-}BrC_6H_4)_2]_n$ (**1b**) at x20000 magnification.

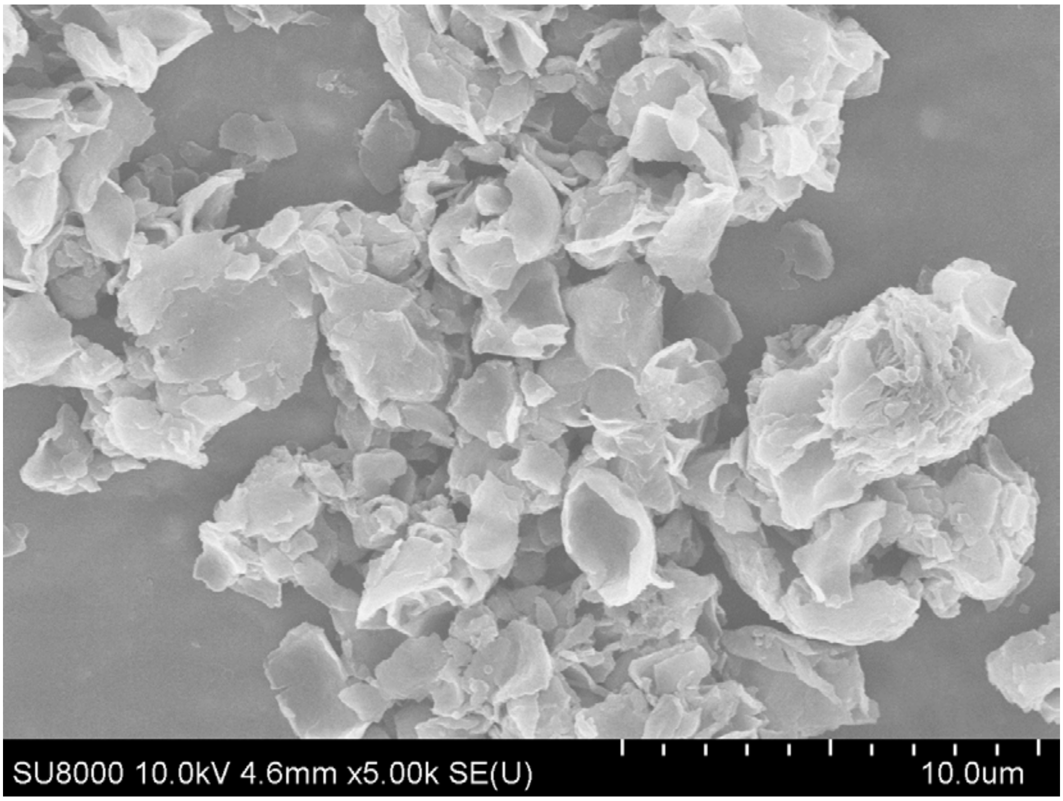

Supplementary Figure 8. FE-SEM image of $[Ni(Sp\text{-}CH_3C_6H_4)_2]_n$ (**1c**) at x5000 magnification.

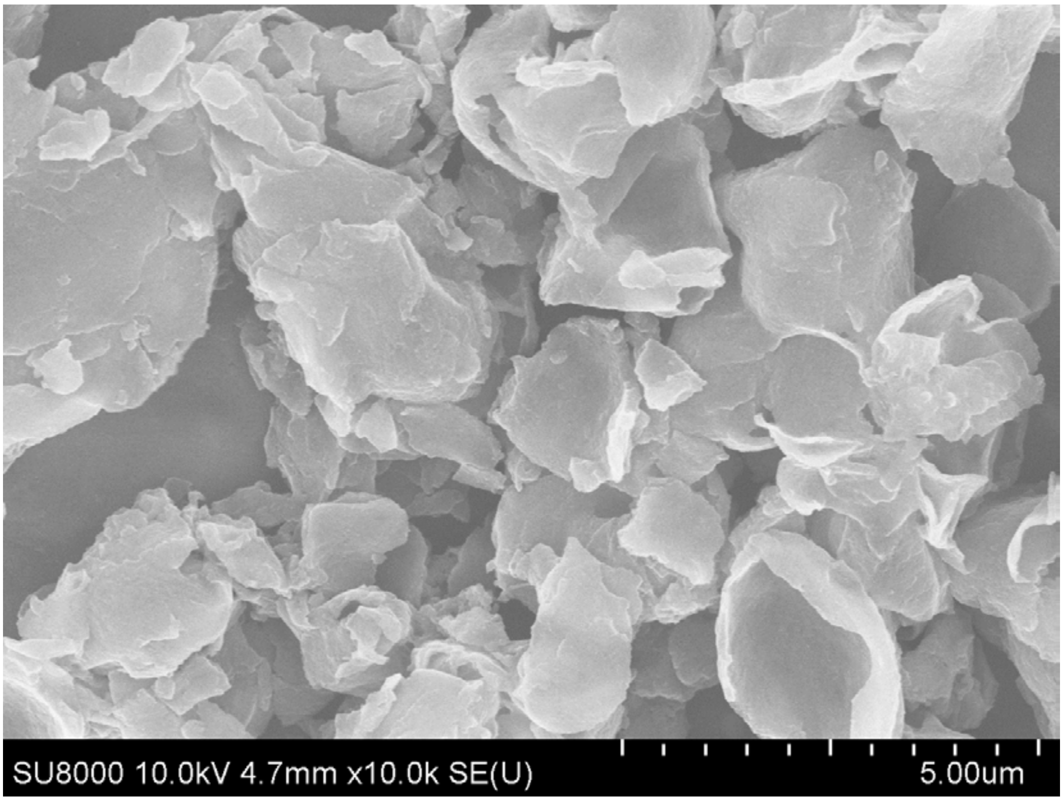

Supplementary Figure 9. FE-SEM image of $[Ni(S\textit{p}\text{-}CH_3C_6H_4)_2]_n$ (**1c**) at x10000 magnification.

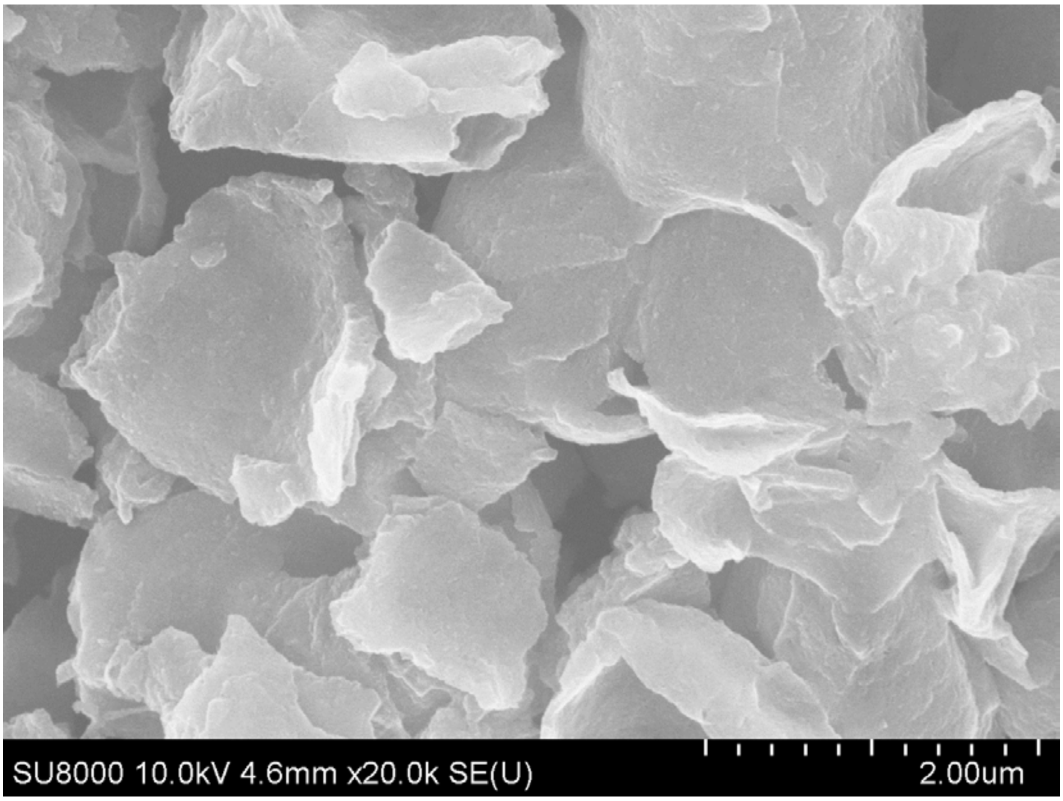

Supplementary Figure 10. FE-SEM image of $[Ni(S\textit{p}\text{-}CH_3C_6H_4)_2]_n$ (**1c**) at x20000 magnification.

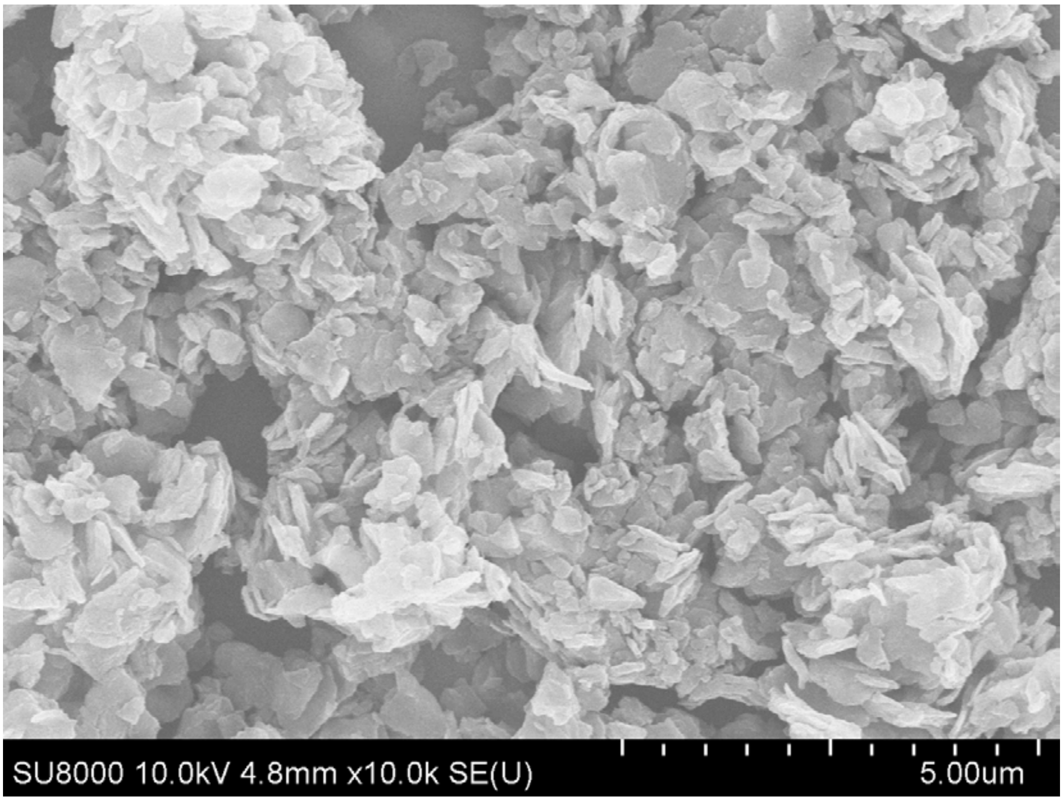

Supplementary Figure 11. FE-SEM image of [Ni(Sm-FC$_6$H$_4$)$_2$]$_n$ (**1d**) at x10000 magnification.

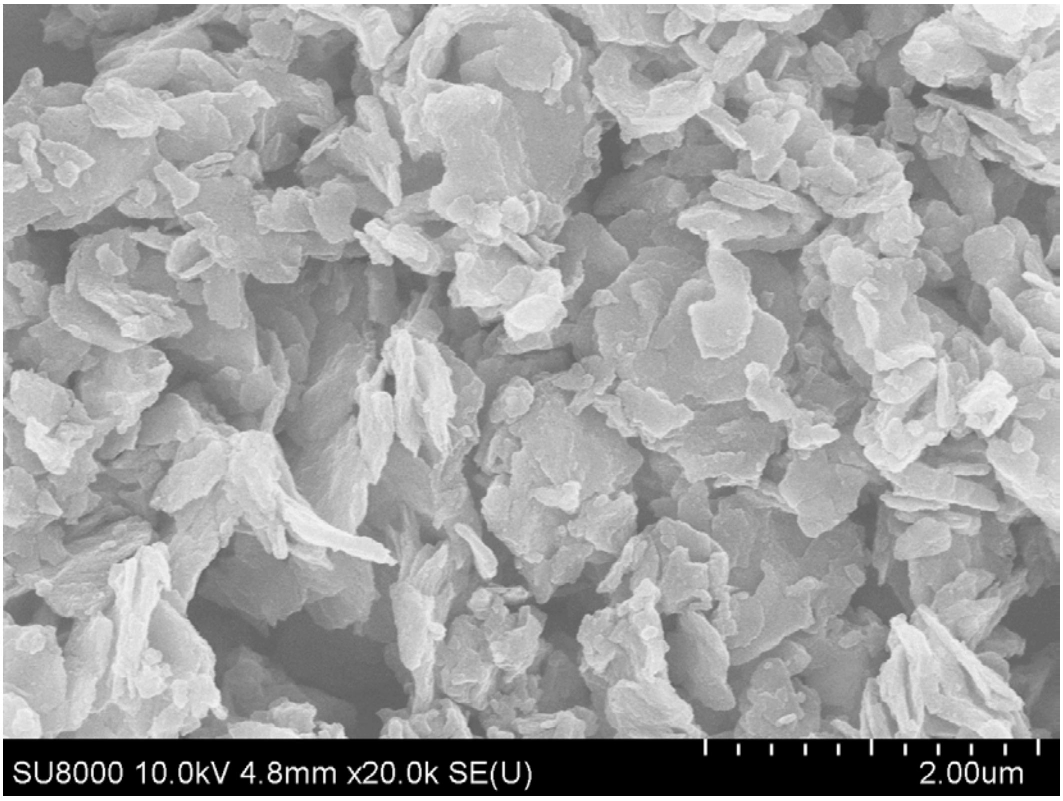

Supplementary Figure 12. FE-SEM image of [Ni(Sm-FC$_6$H$_4$)$_2$]$_n$ (**1d**) at x20000 magnification.

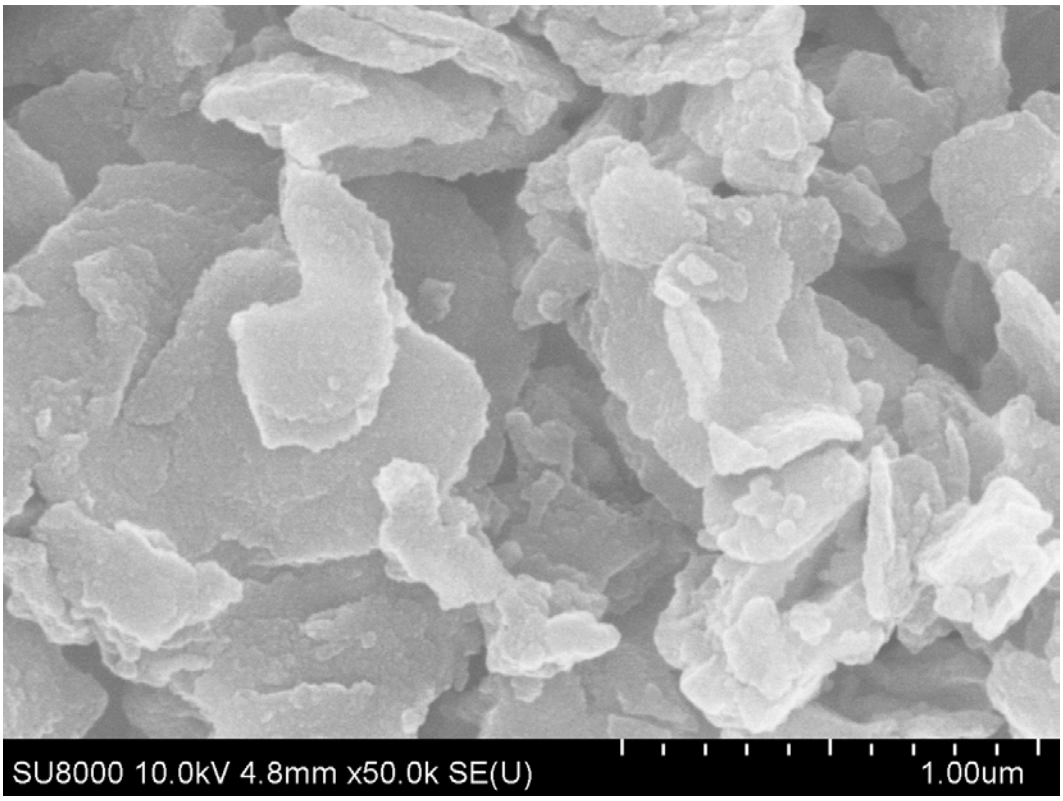

Supplementary Figure 13. FE-SEM image of [Ni(S*m*-FC_6H_4)$_2$]$_n$ (**1d**) at x50000 magnification.

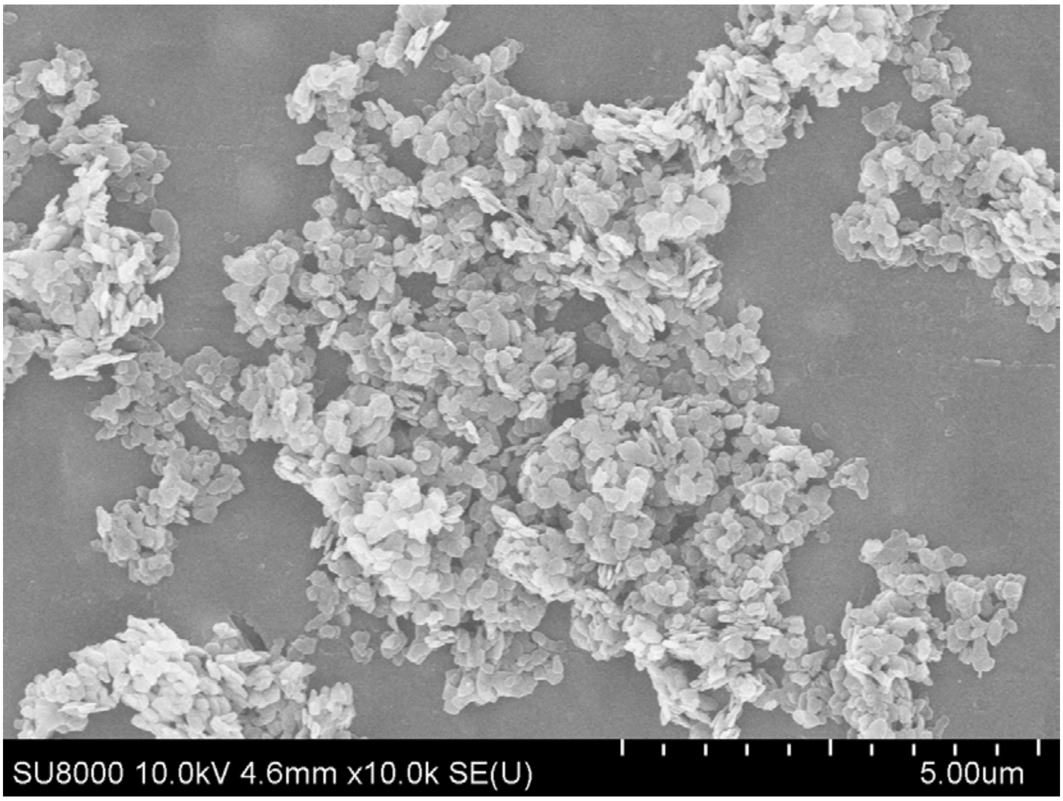

Supplementary Figure 14. FE-SEM image of [Ni(S*o*-$NH_2C_6H_4$)$_2$]$_n$ (**1e**) at x10000 magnification.

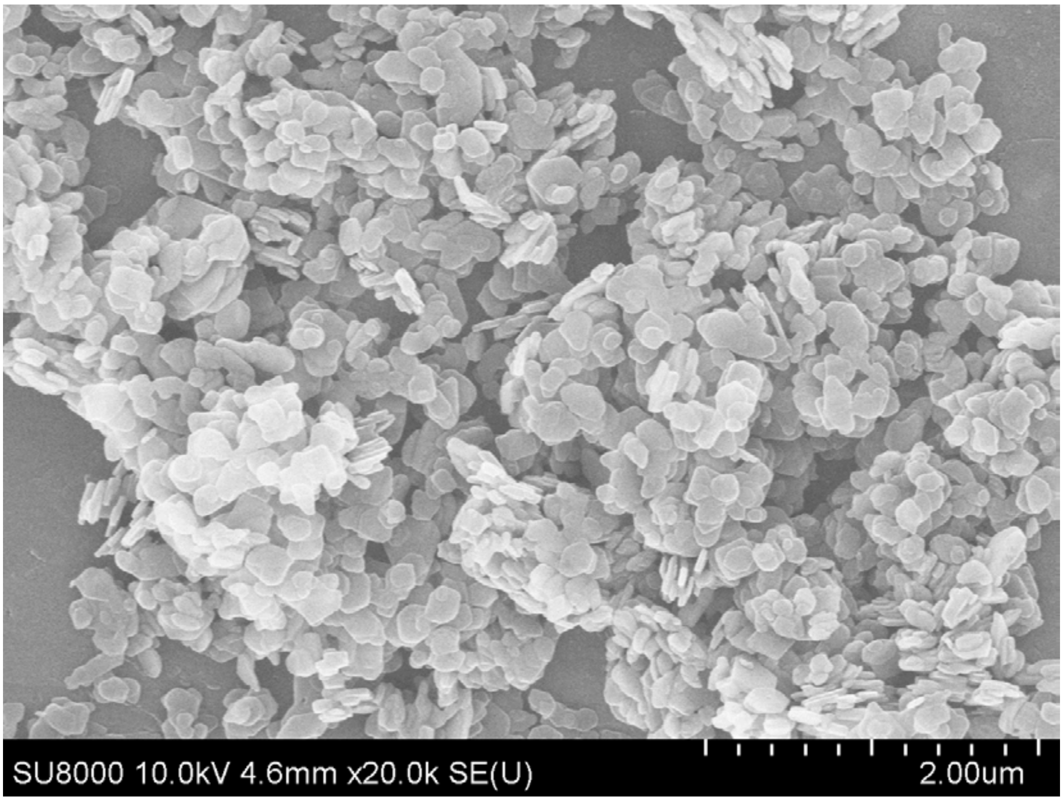

Supplementary Figure 15. FE-SEM image of $[Ni(S\textit{o}\text{-}NH_2C_6H_4)_2]_n$ (**1e**) at x20000 magnification.

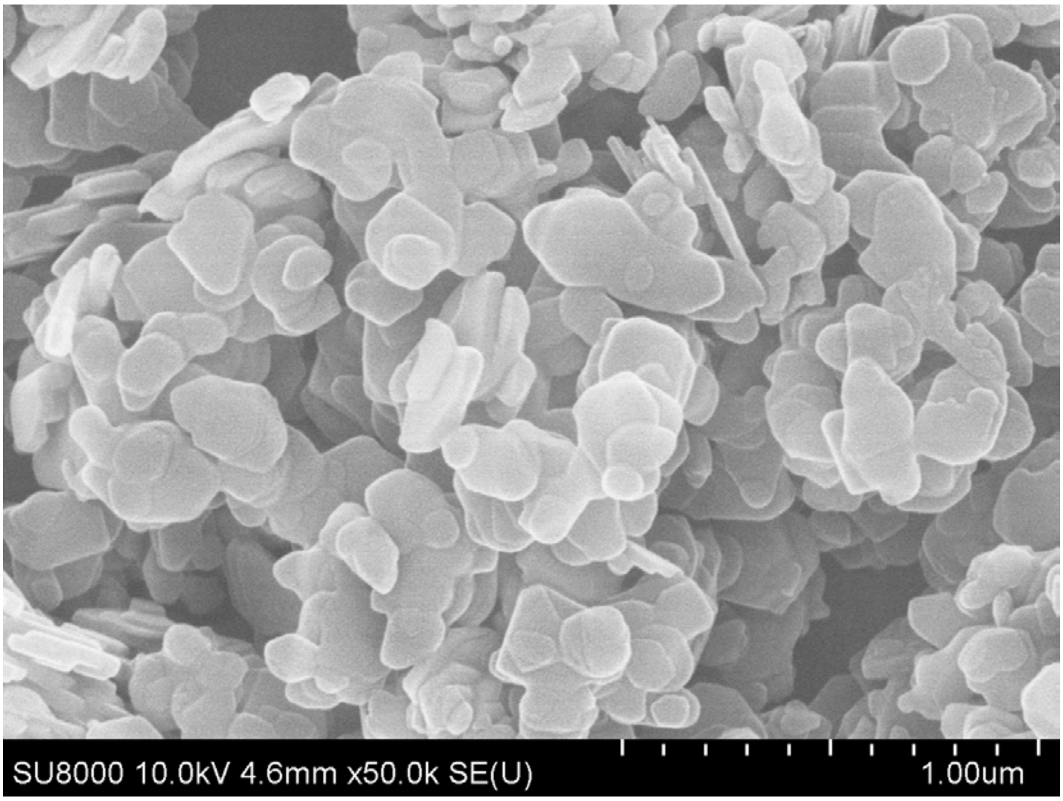

Supplementary Figure 16. FE-SEM image of $[Ni(S\textit{o}\text{-}NH_2C_6H_4)_2]_n$ (**1e**) at x50000 magnification.

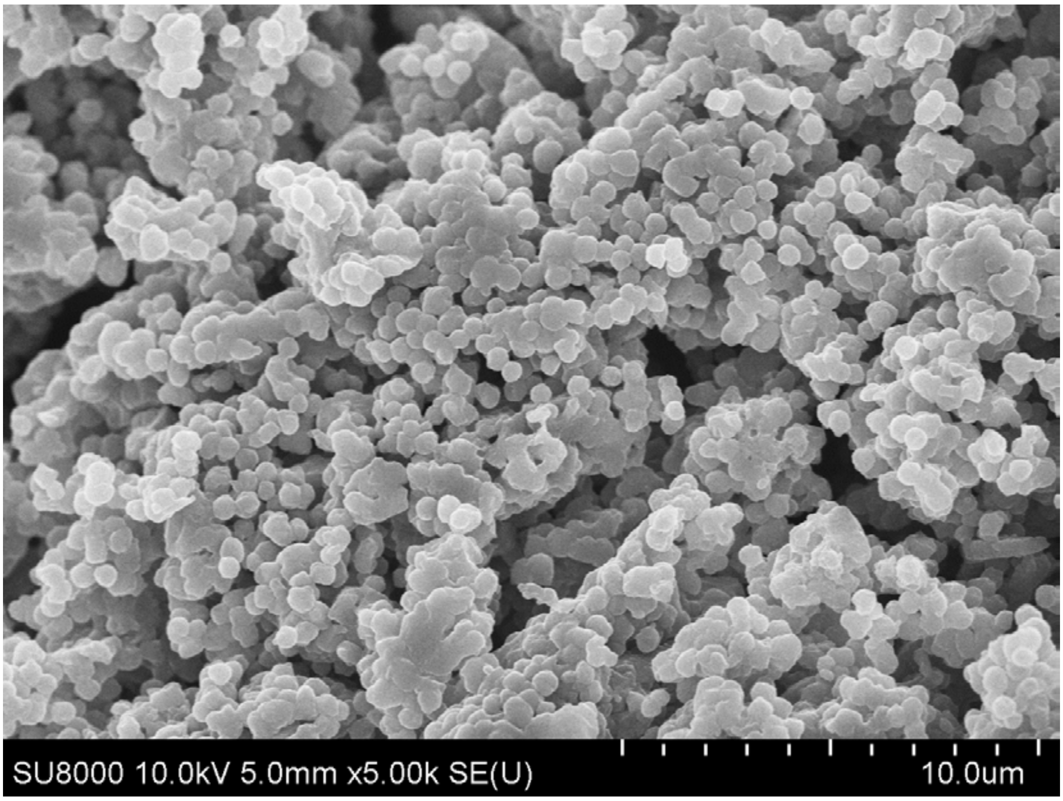

Supplementary Figure 17. FE-SEM image of [Ni(S*p*-OHC$_6$H$_4$)$_2$]$_n$ (**1f**) at x5000 magnification.

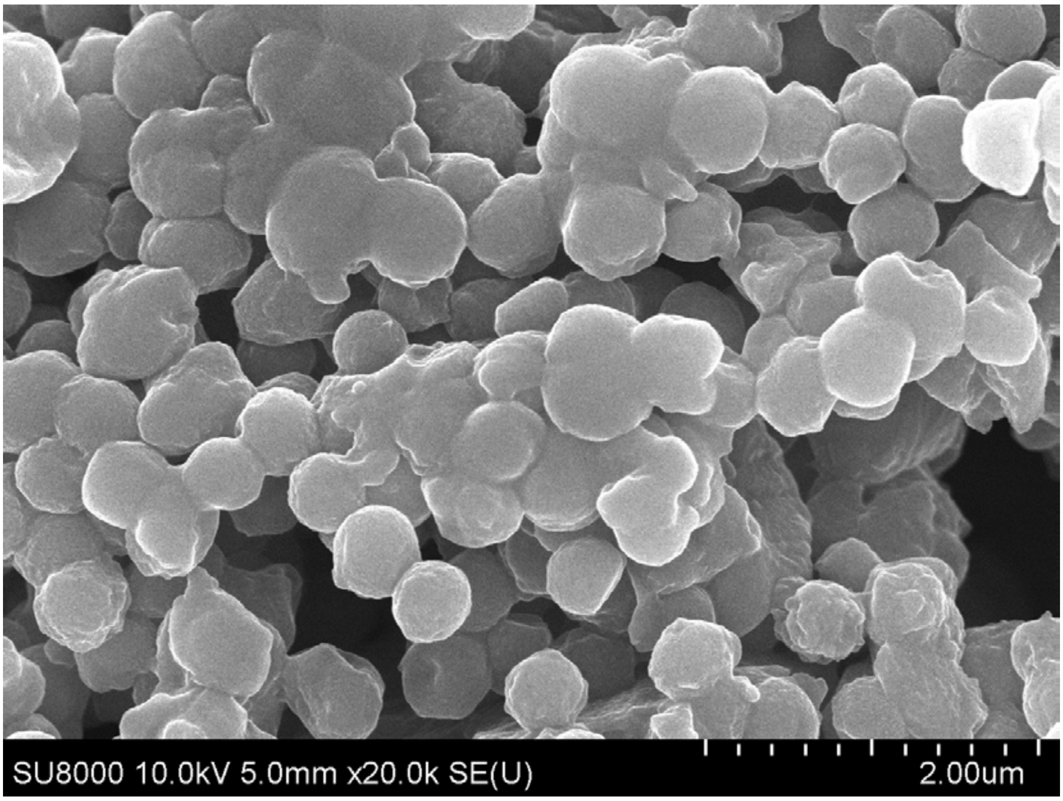

Supplementary Figure 18. FE-SEM image of [Ni(S*p*-OHC$_6$H$_4$)$_2$]$_n$ (**1f**) at x20000 magnification.

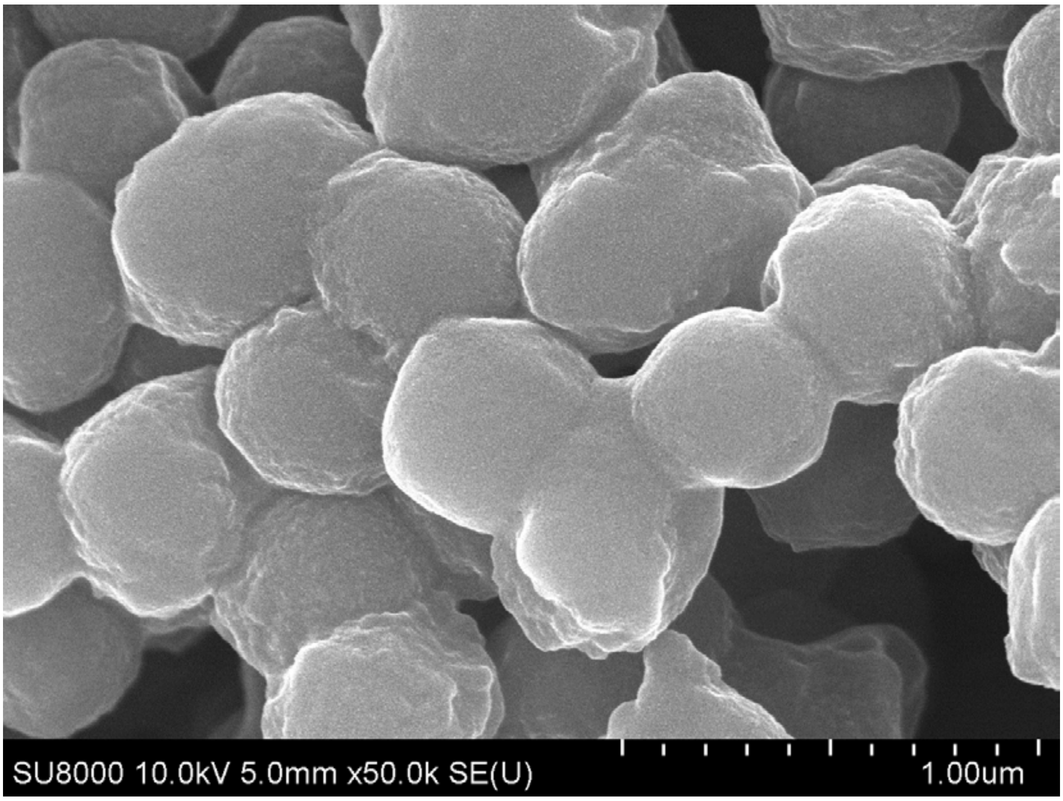

Supplementary Figure 19. FE-SEM image of $[Ni(Sp\text{-}OHC_6H_4)_2]_n$ (**1f**) at x50000 magnification.

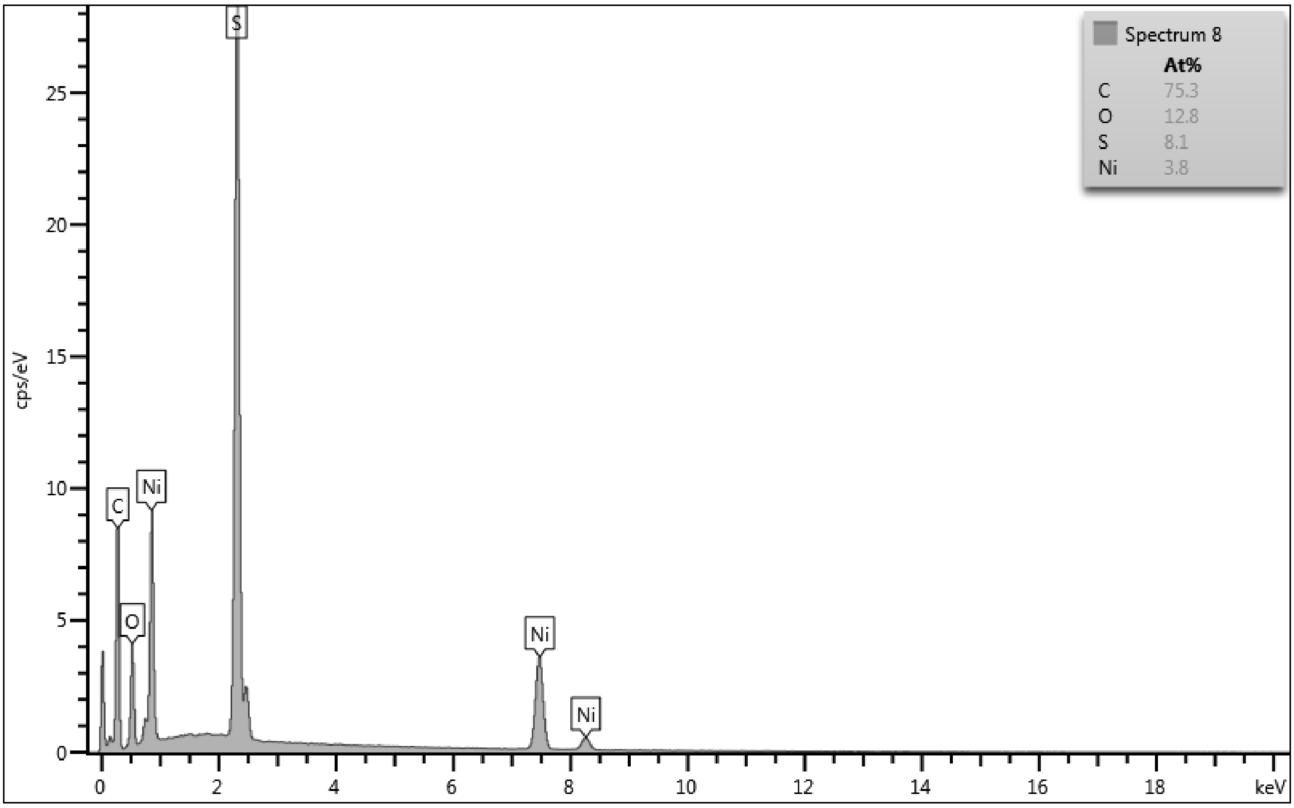

Supplementary Figure 20. EDX spectrum of $[Ni(Sp\text{-}OHC_6H_4)_2]_n$ (**1f**).

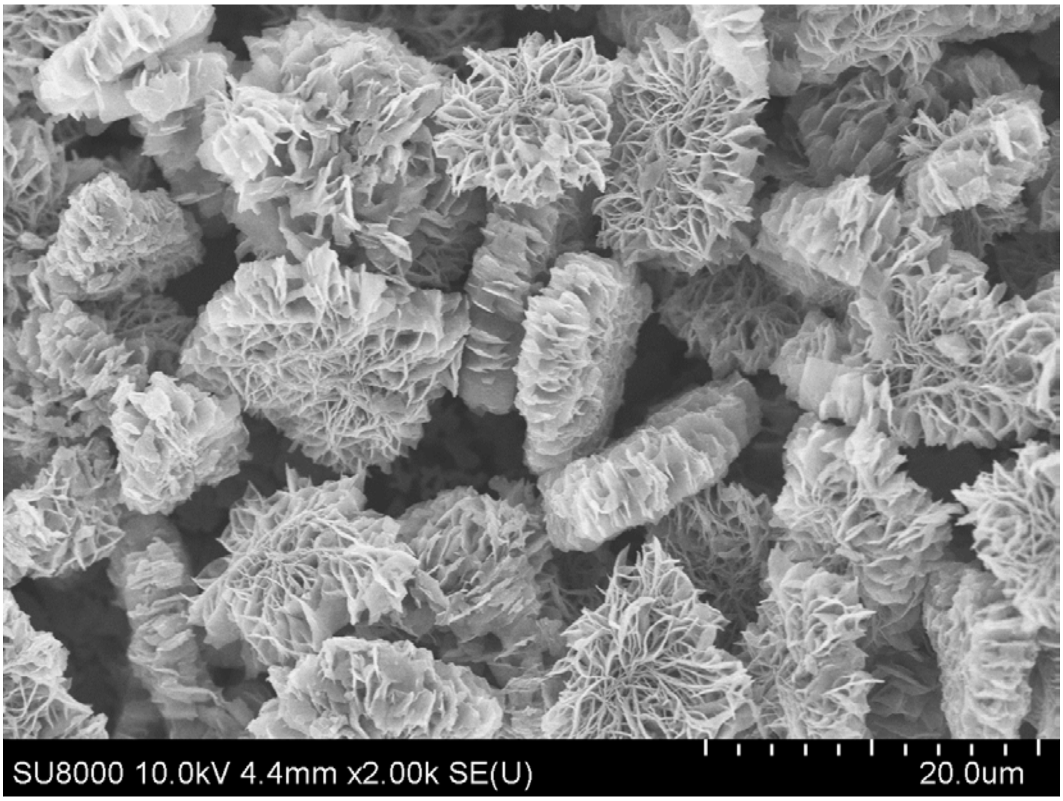

Supplementary Figure 21. FE-SEM image of [Ni(S*p*-ClC$_6$H$_4$)$_2$]$_n$ (**1g**) at x2000 magnification.

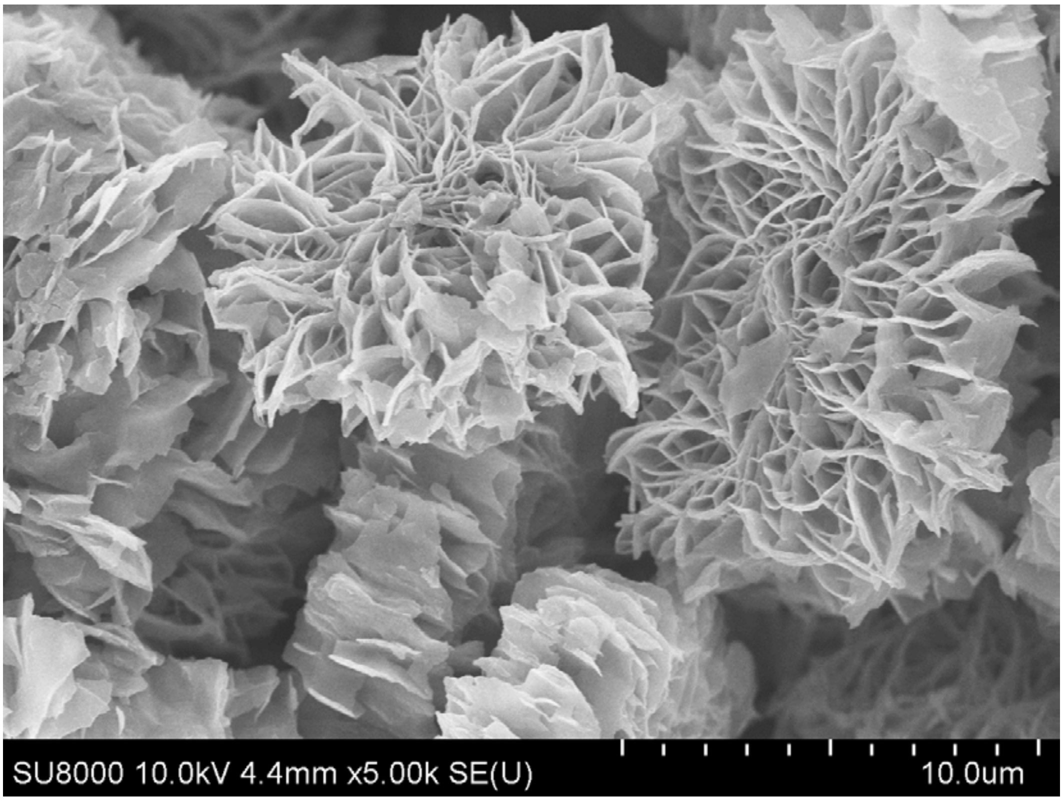

Supplementary Figure 22. FE-SEM image of [Ni(S*p*-ClC$_6$H$_4$)$_2$]$_n$ (**1g**) at x5000 magnification.

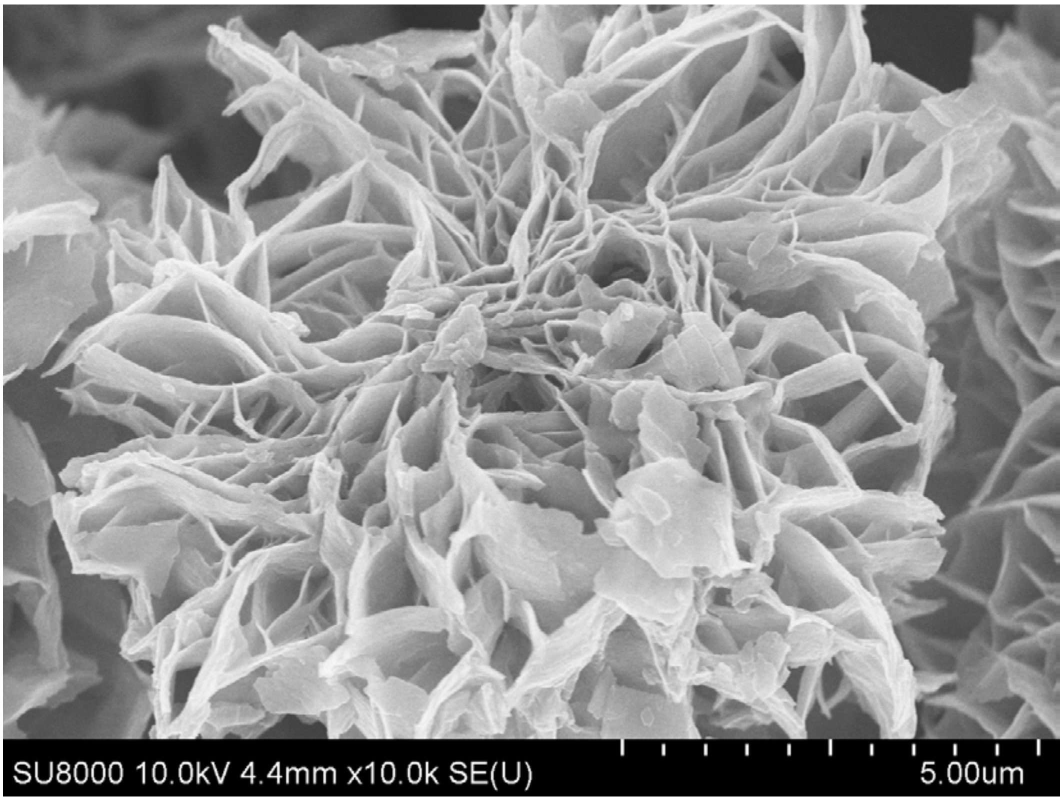

Supplementary Figure 23. FE-SEM image of $[Ni(S\textit{p}\text{-}ClC_6H_4)_2]_n$ (**1g**) at x10000 magnification.

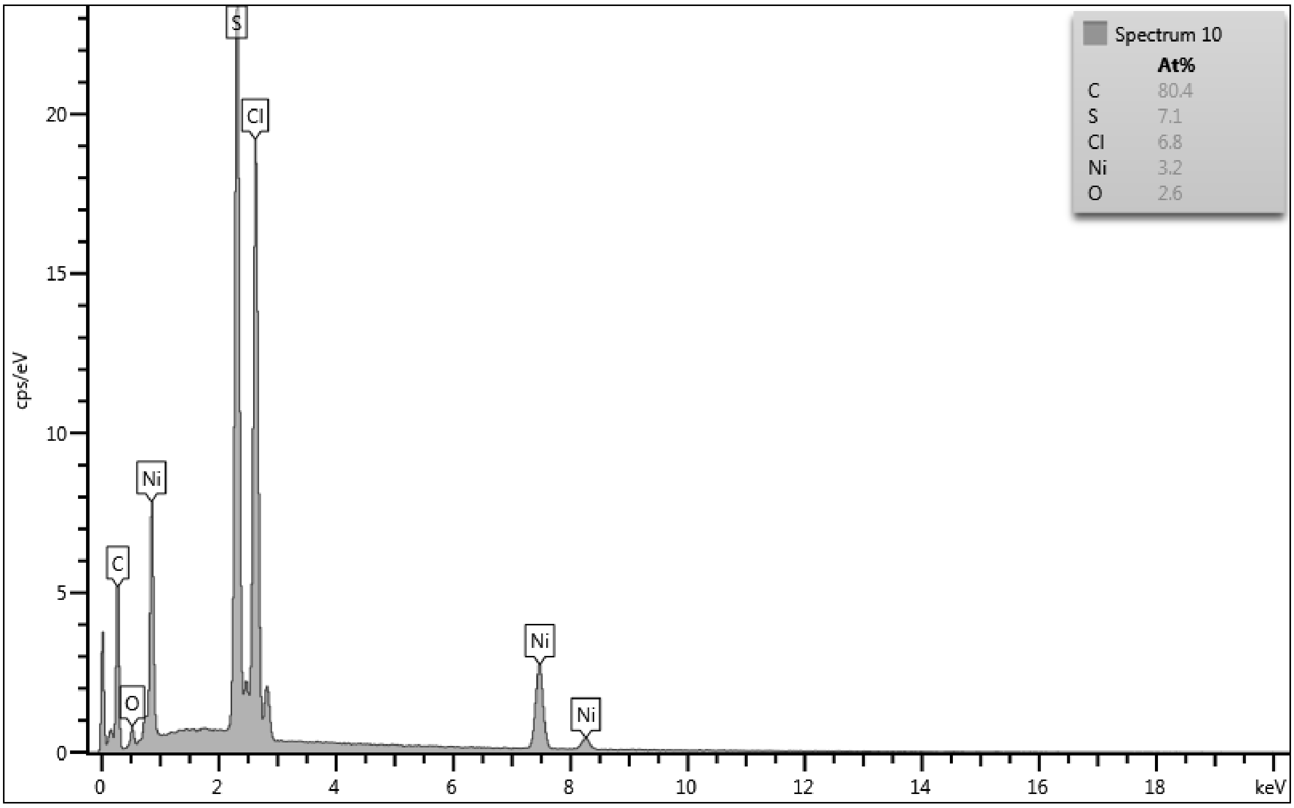

Supplementary Figure 24. EDX spectrum of $[Ni(S\textit{p}\text{-}ClC_6H_4)_2]_n$ (**1g**).

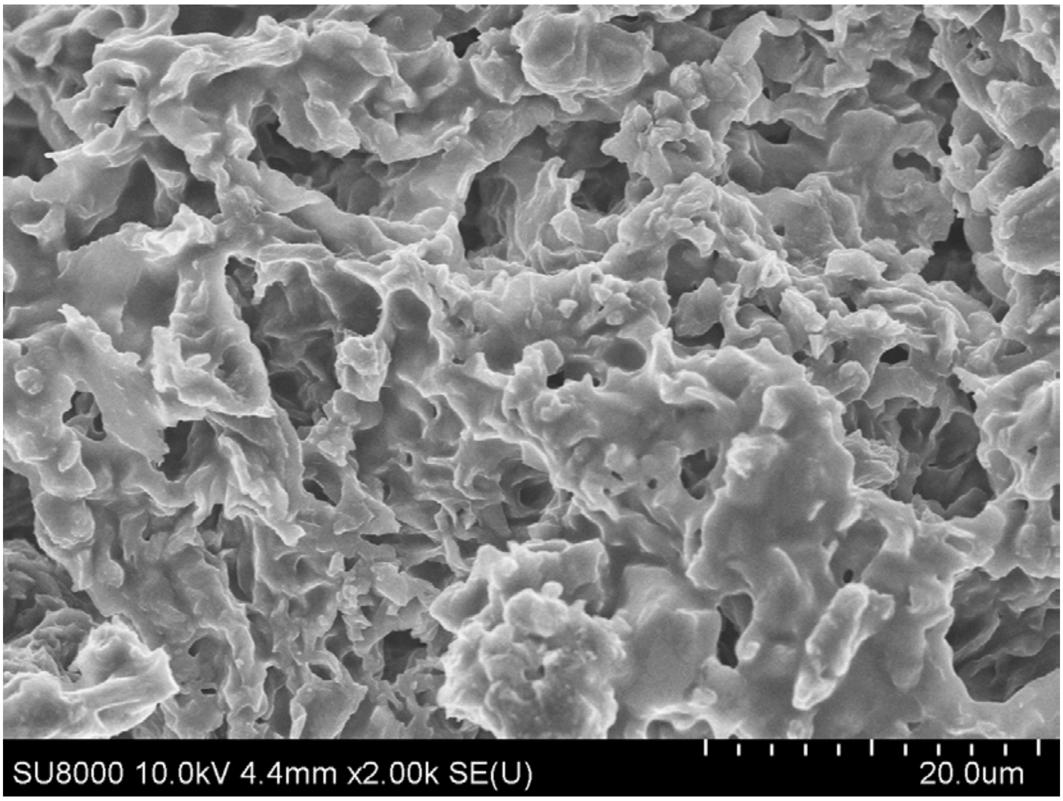

Supplementary Figure 25. FE-SEM image of $[Ni(S\textit{p}\text{-}OCH_3C_6H_4)_2]_n$ (**1h**) at x2000 magnification.

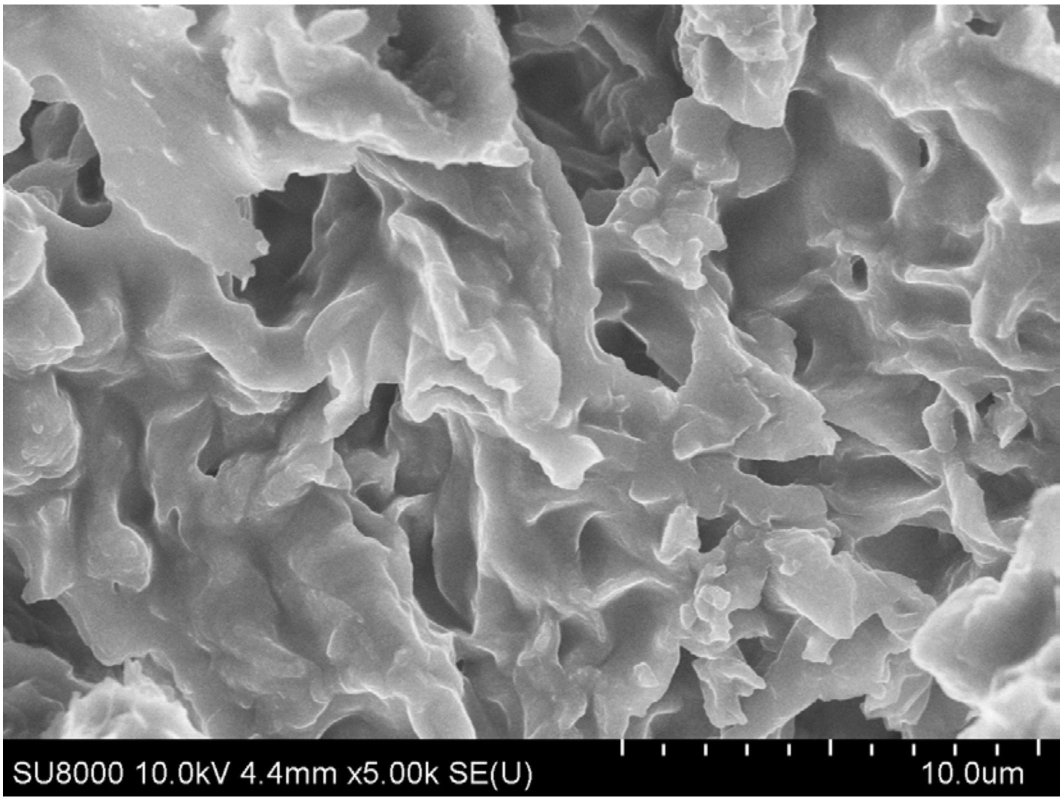

Supplementary Figure 26. FE-SEM image of $[Ni(S\textit{p}\text{-}OCH_3C_6H_4)_2]_n$ (**1h**) at x5000 magnification.

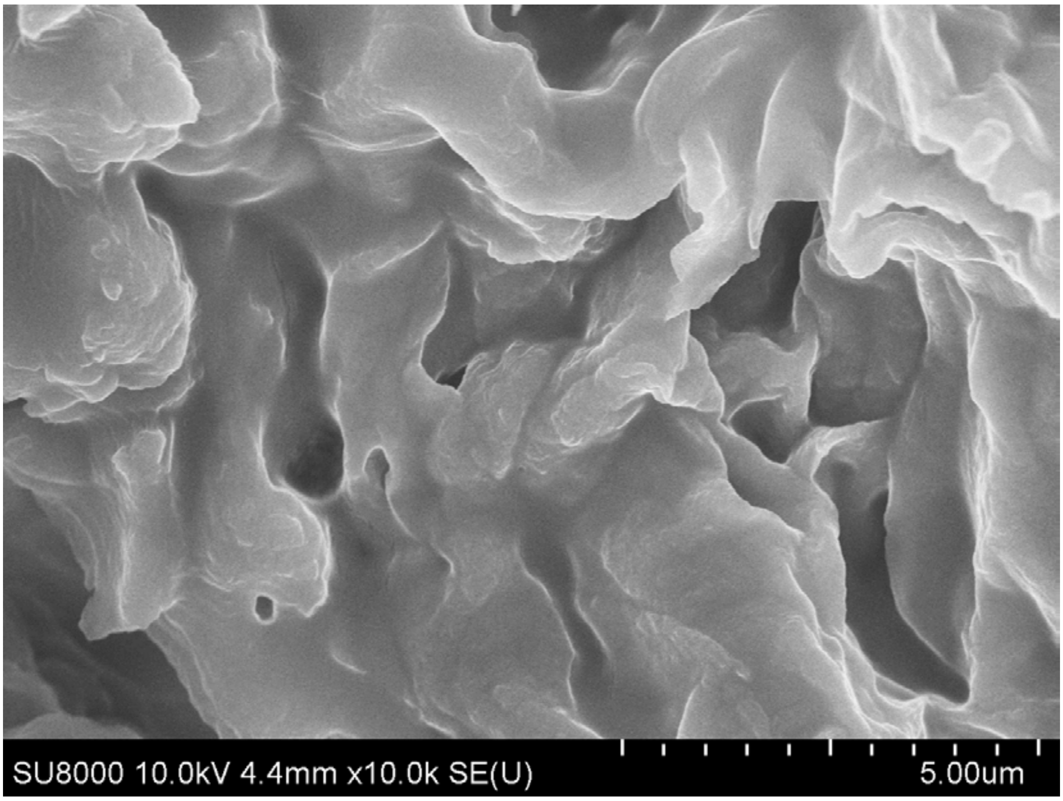

Supplementary Figure 27. FE-SEM image of [Ni(Sp-$OCH_3C_6H_4$)$_2$]$_n$ (**1h**) at x10000 magnification.

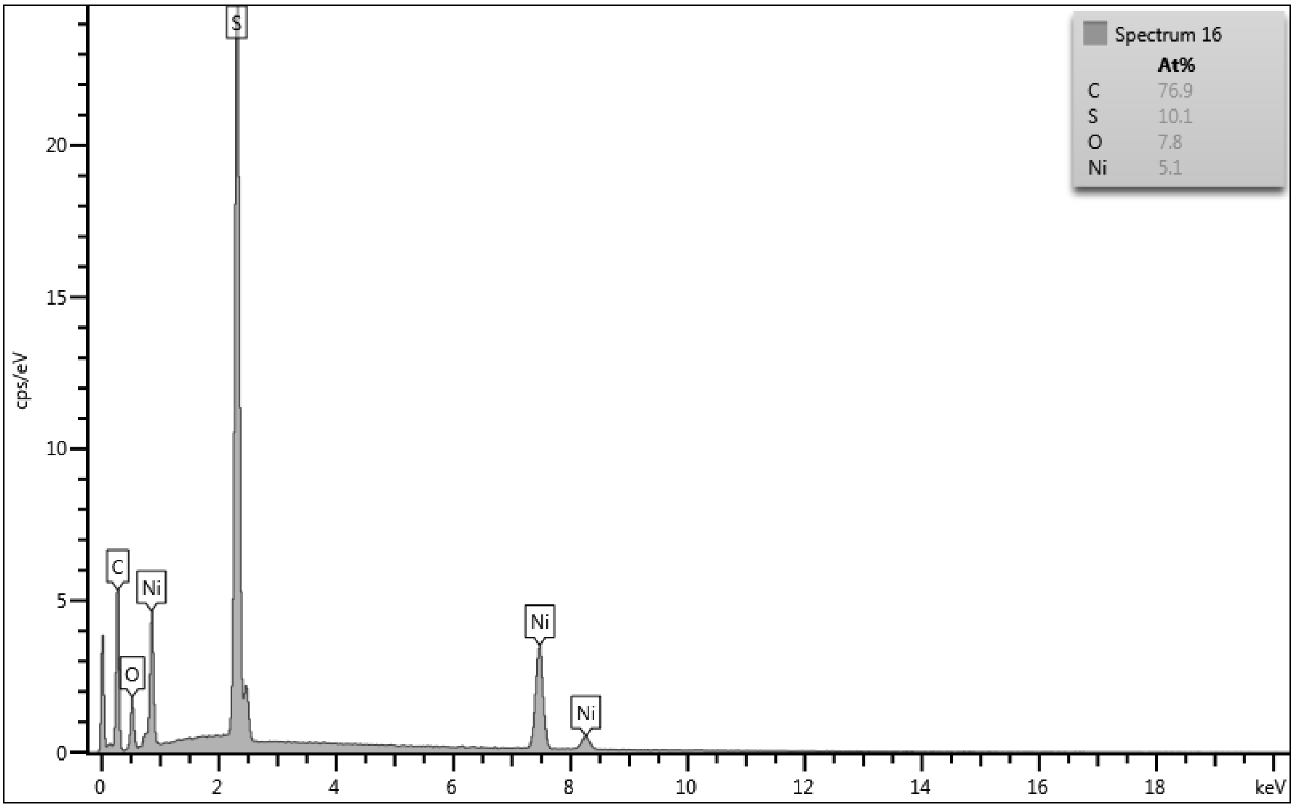

Supplementary Figure 28. EDX spectrum of [Ni(Sp-$OCH_3C_6H_4$)$_2$]$_n$ (**1h**).

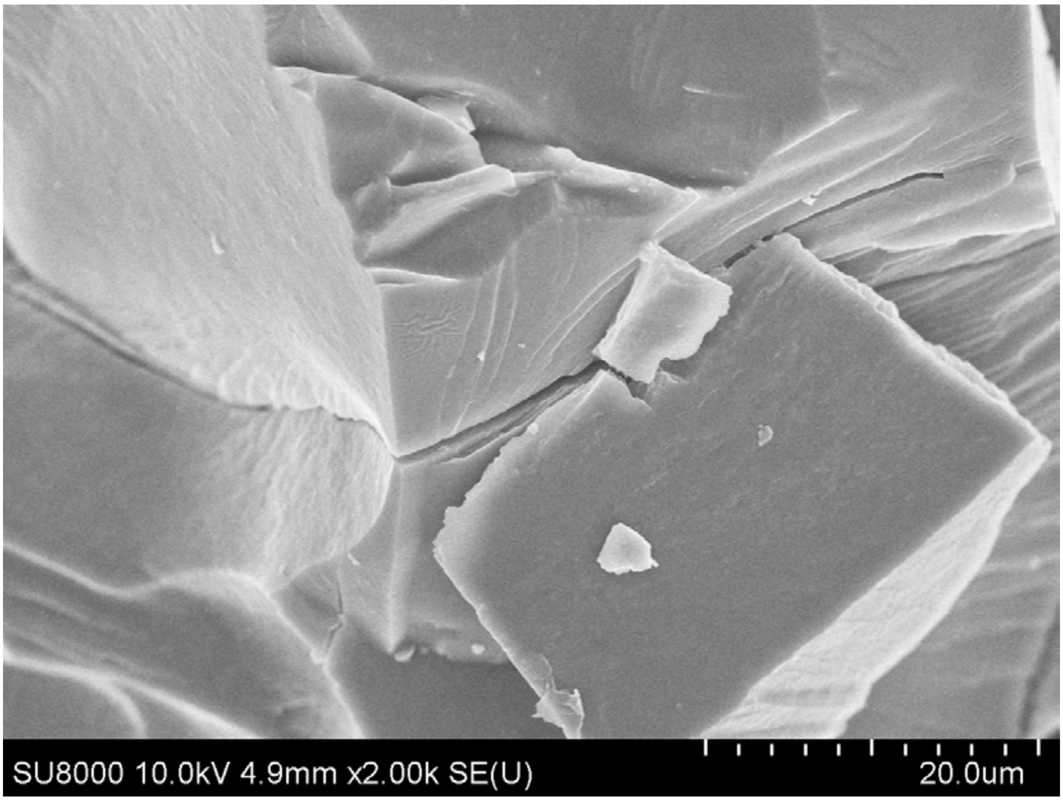

Supplementary Figure 29. FE-SEM image of [Ni(S*p*-$NH_2C_6H_4)_2]_n$ (**1i**) at x2000 magnification.

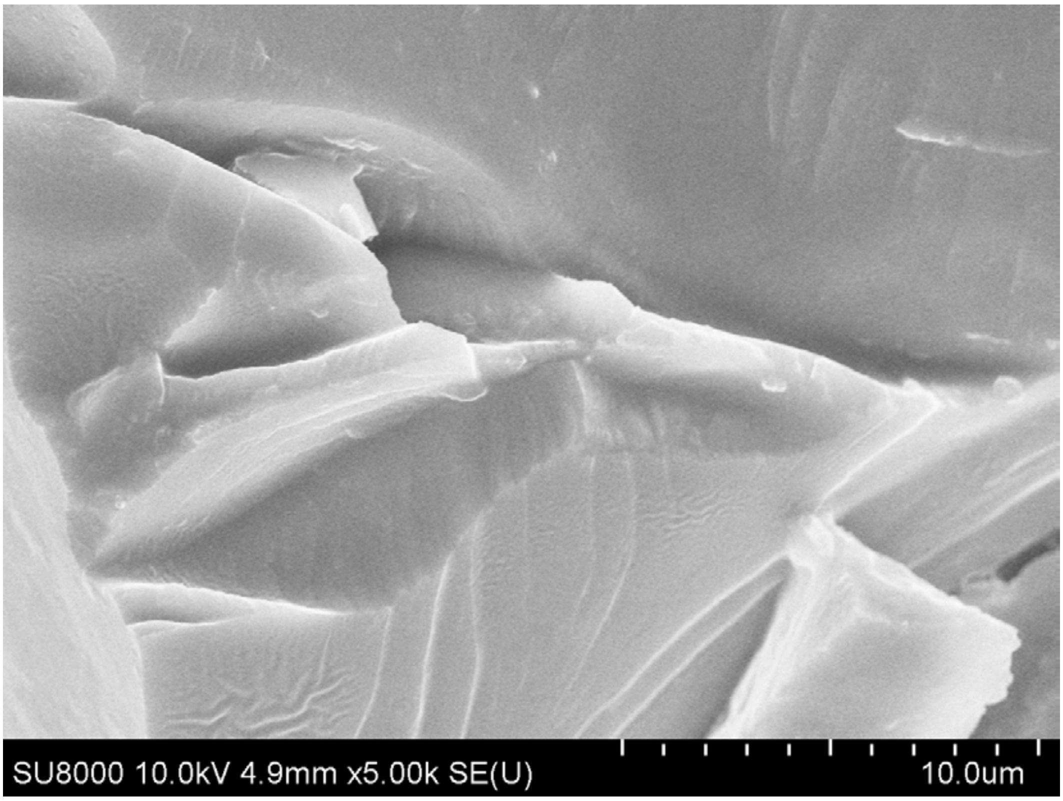

Supplementary Figure 30. FE-SEM image of [Ni(S*p*-$NH_2C_6H_4)_2]_n$ (**1i**) at x5000 magnification.

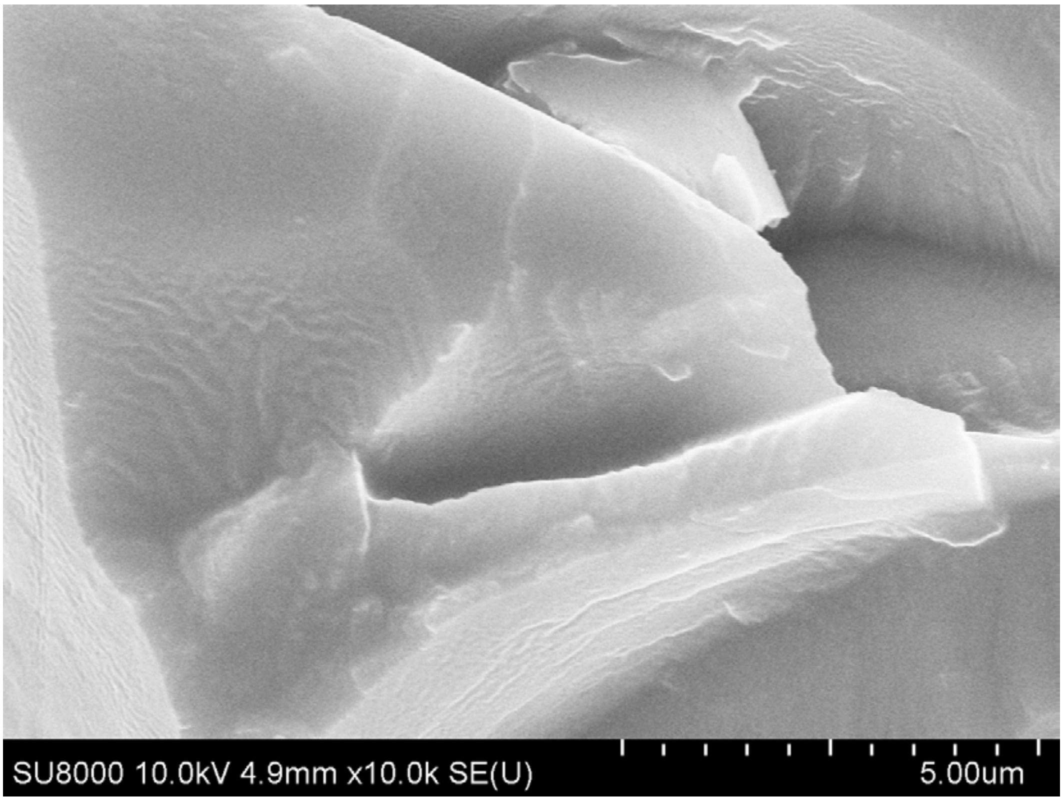

Supplementary Figure 31. FE-SEM image of [Ni(Sp-$NH_2C_6H_4)_2]_n$ (**1i**) at x10000 magnification.

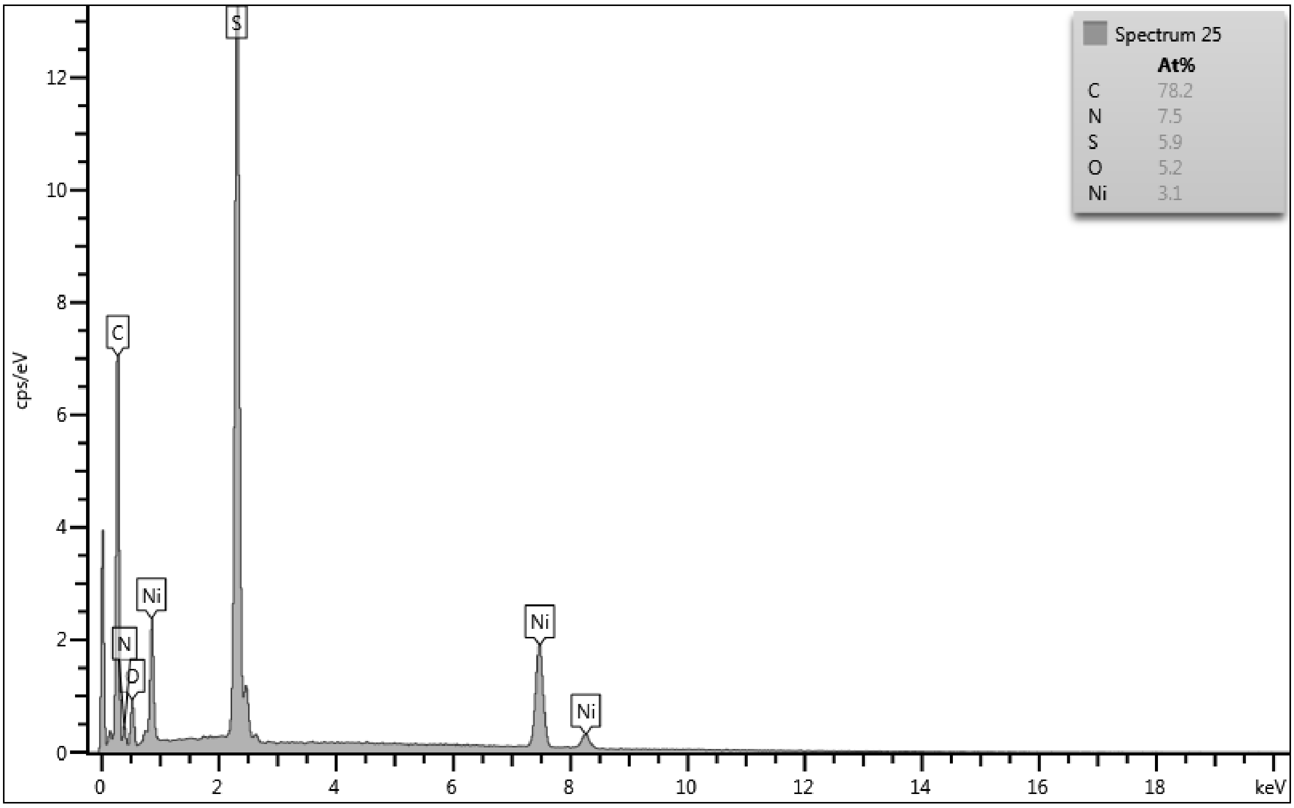

Supplementary Figure 32. EDX spectrum of [Ni(Sp-$NH_2C_6H_4)_2]_n$ (**1i**).

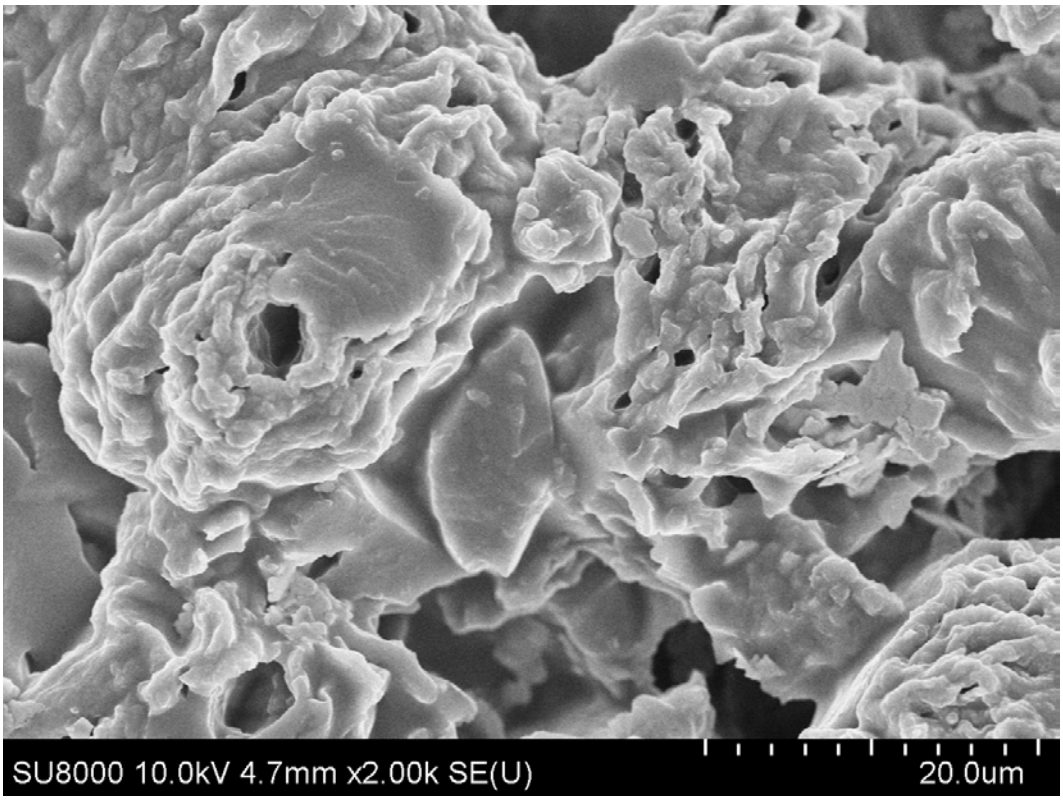

Supplementary Figure 33. FE-SEM image of $[Ni(S\textit{m}\text{-}(CH_3)_2C_6H_3)_2]_n$ (**1j**) at x2000 magnification.

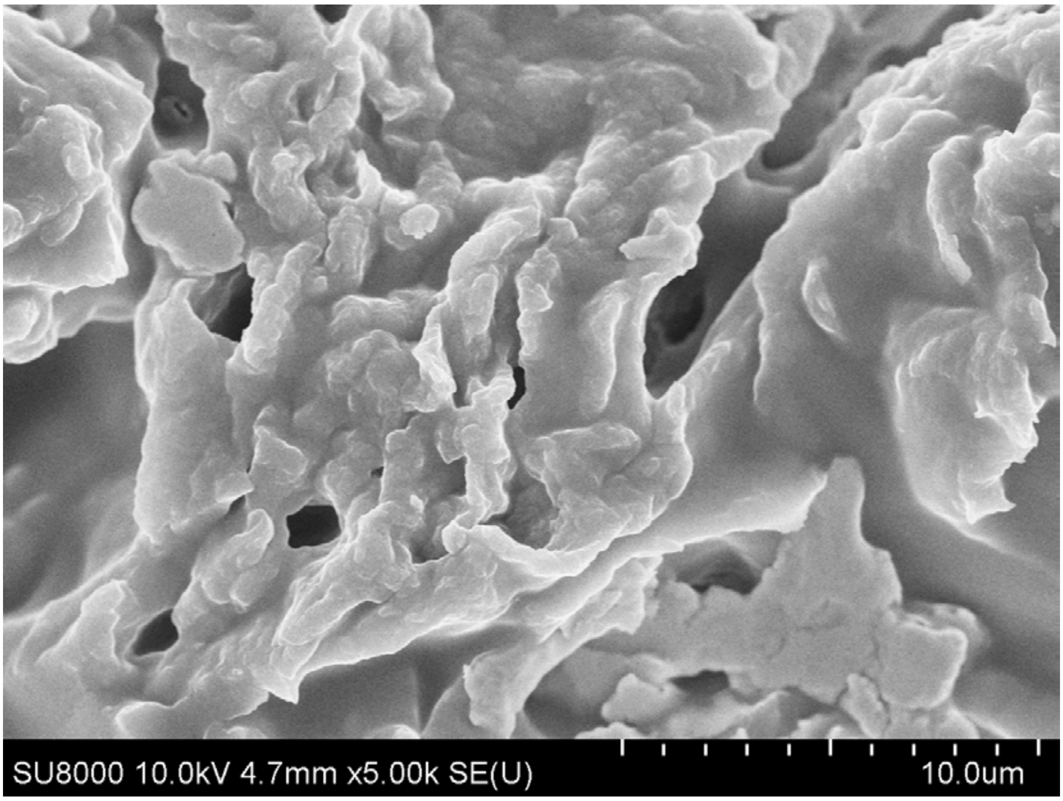

Supplementary Figure 34. FE-SEM image of $[Ni(S\textit{m}\text{-}(CH_3)_2C_6H_3)_2]_n$ (**1j**) at x5000 magnification.

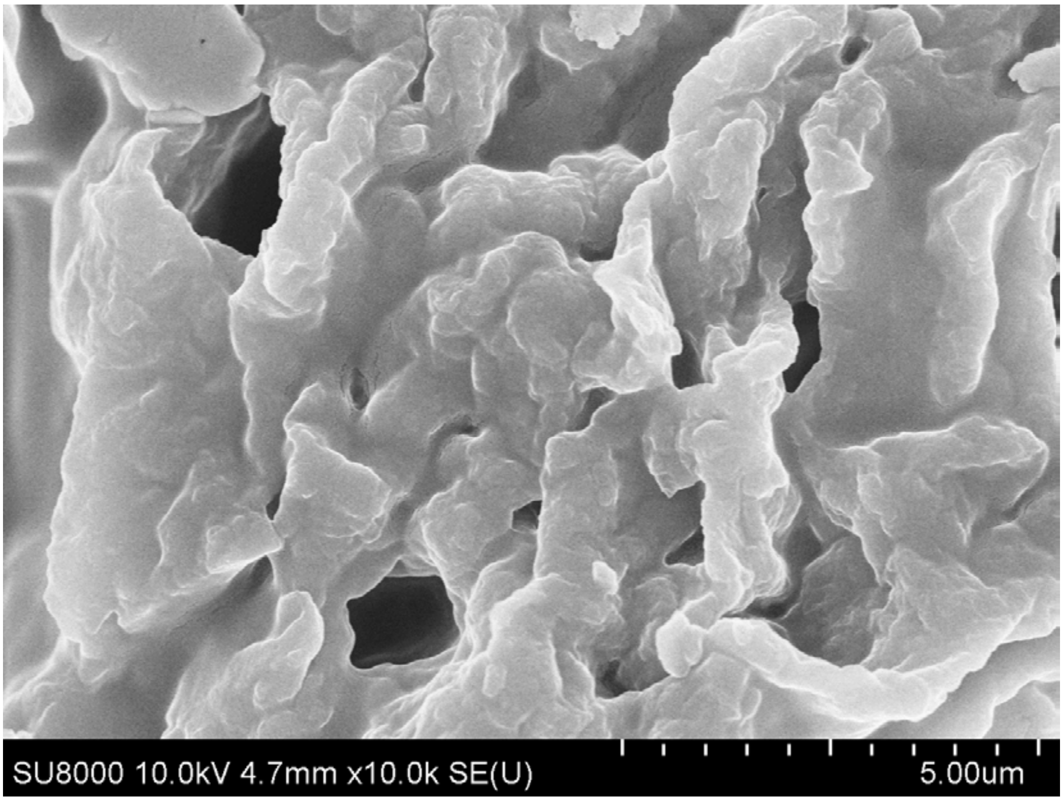

Supplementary Figure 35. FE-SEM image of $[Ni(S\textit{m}\text{-}(CH_3)_2C_6H_3)_2]_n$ (**1j**) at x10000 magnification.

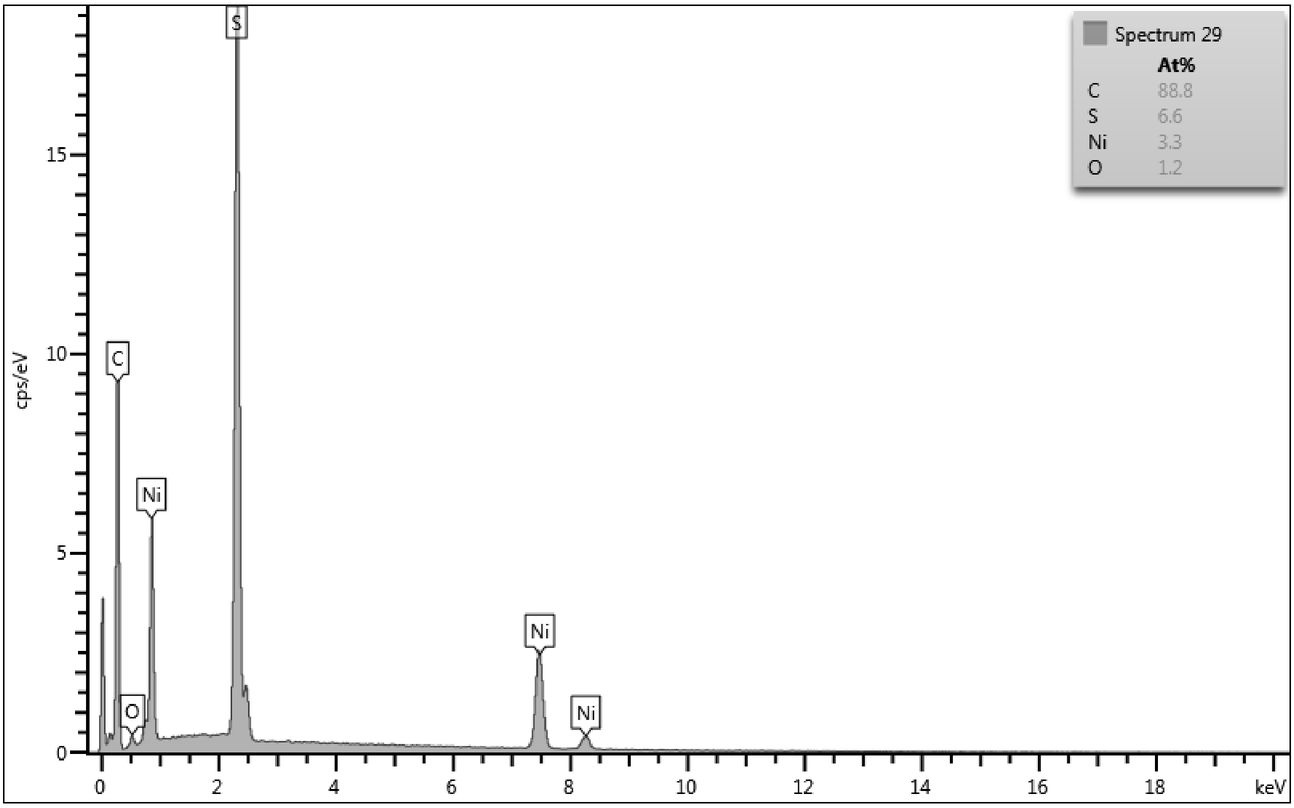

Supplementary Figure 36. EDX spectrum of $[Ni(S\textit{m}\text{-}(CH_3)_2C_6H_3)_2]_n$ (**1j**).

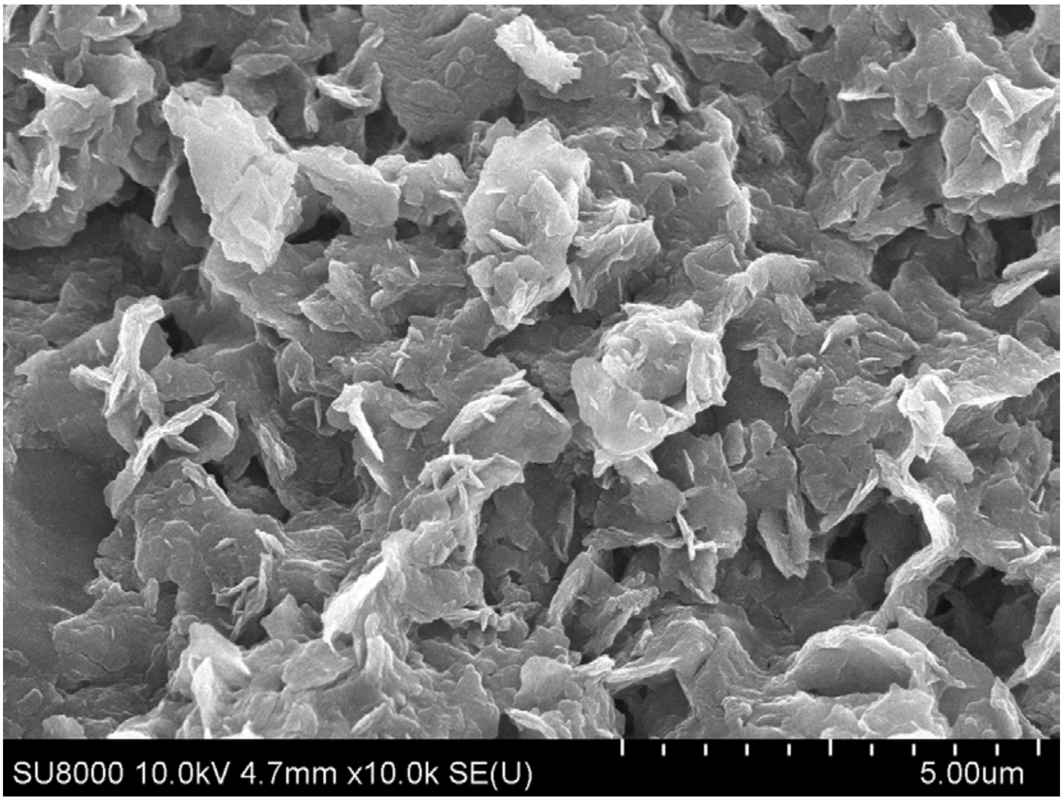

Supplementary Figure 37. FE-SEM image of $[Ni(SCy)_2]_n$ (**1k**) at x10000 magnification.

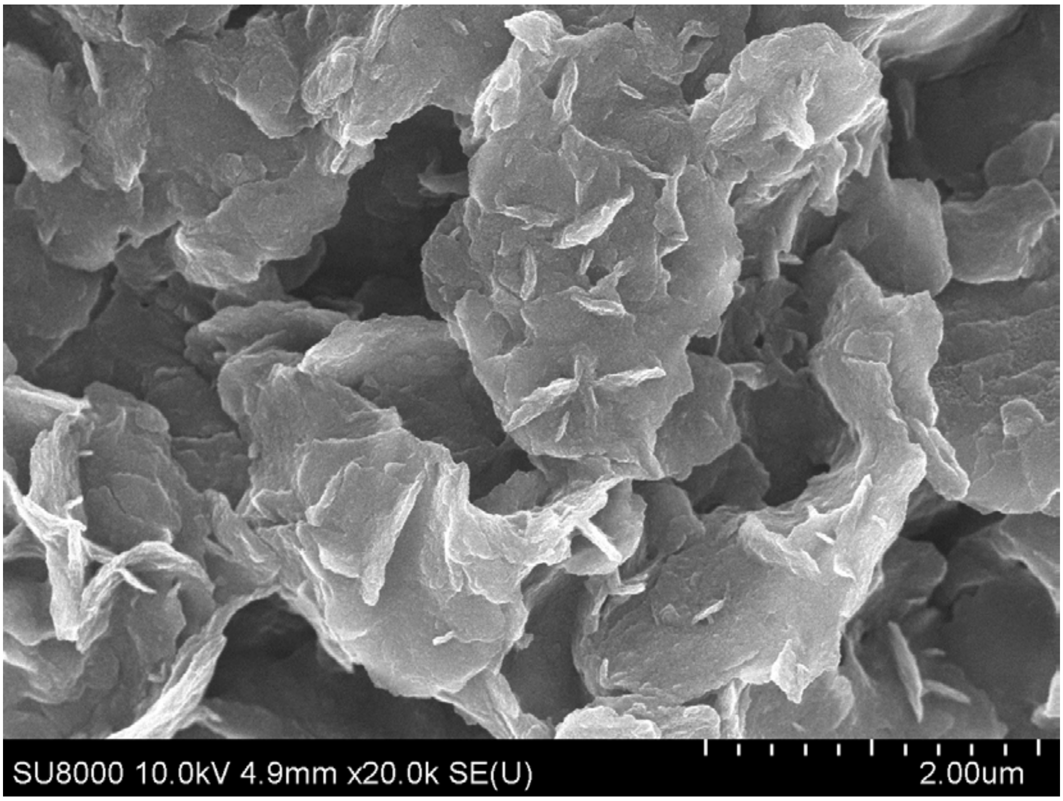

Supplementary Figure 38. FE-SEM image of $[Ni(SCy)_2]_n$ (**1k**) at x20000 magnification.

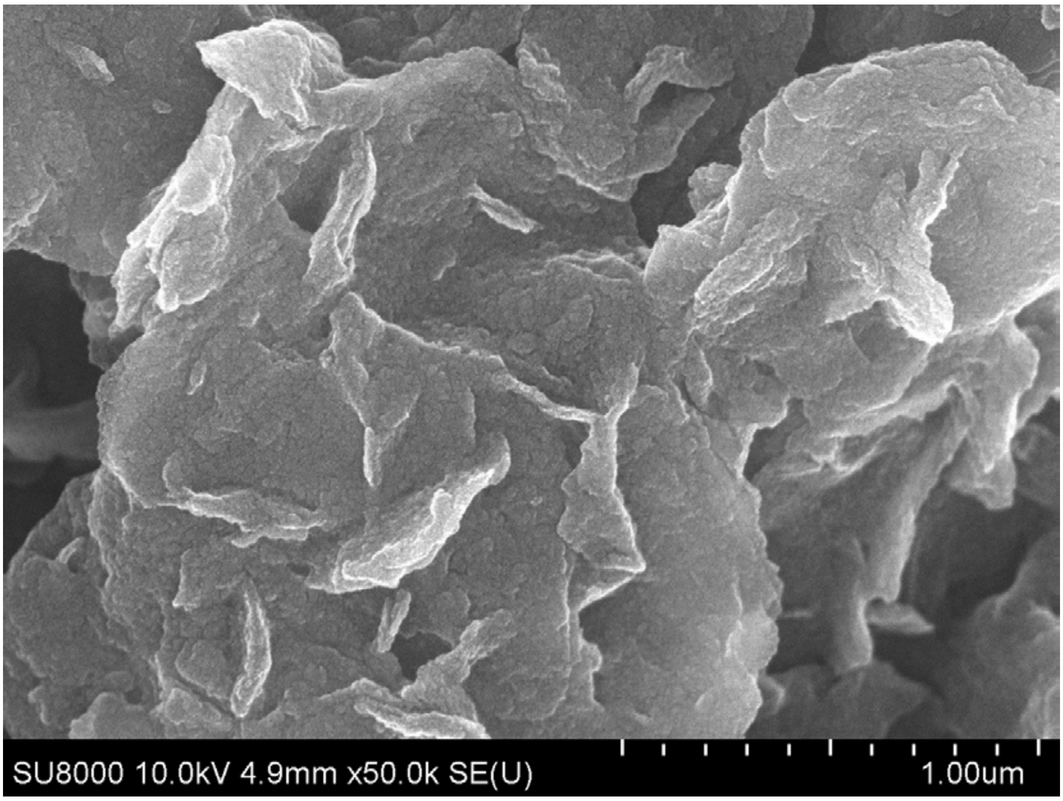

Supplementary Figure 39. FE-SEM image of $[Ni(SCy)_2]_n$ (**1k**) at x50000 magnification.

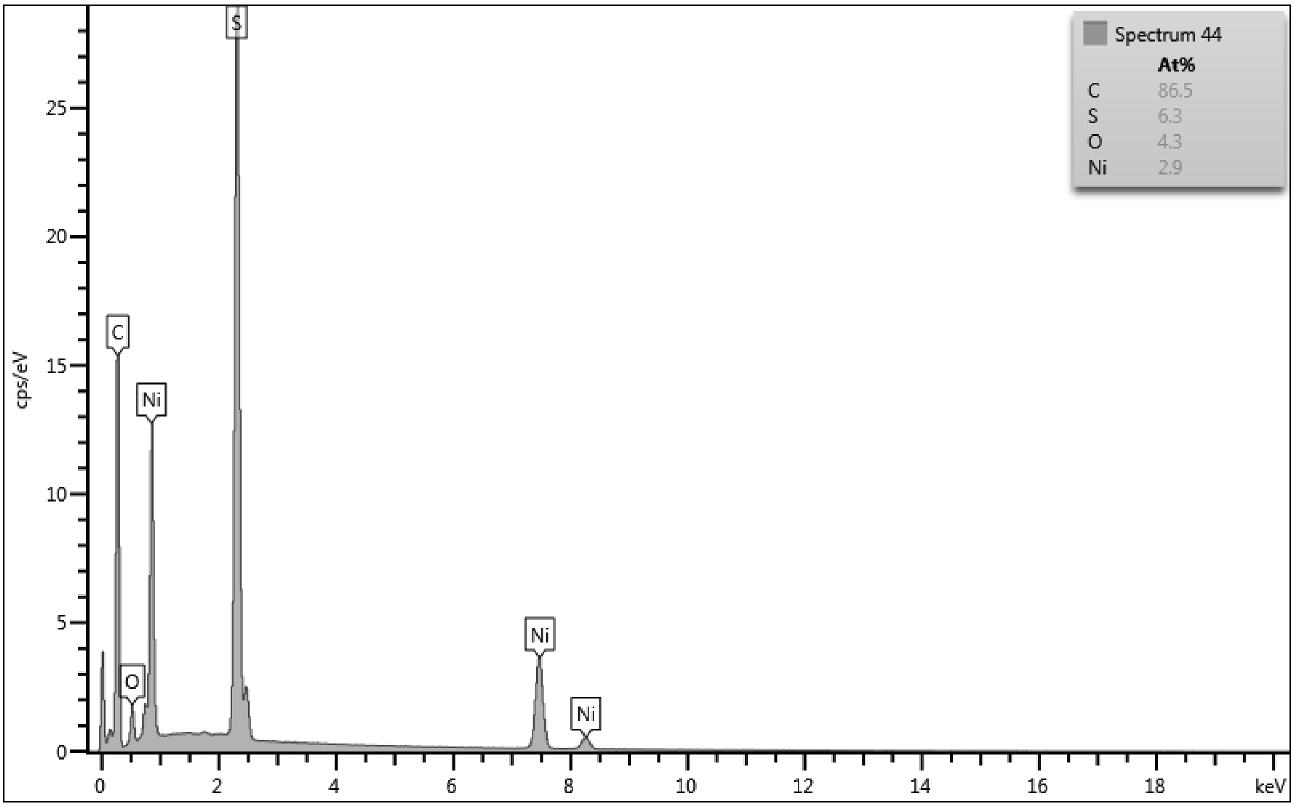

Supplementary Figure 40. EDX spectrum of $[Ni(SCy)_2]_n$ (**1k**).

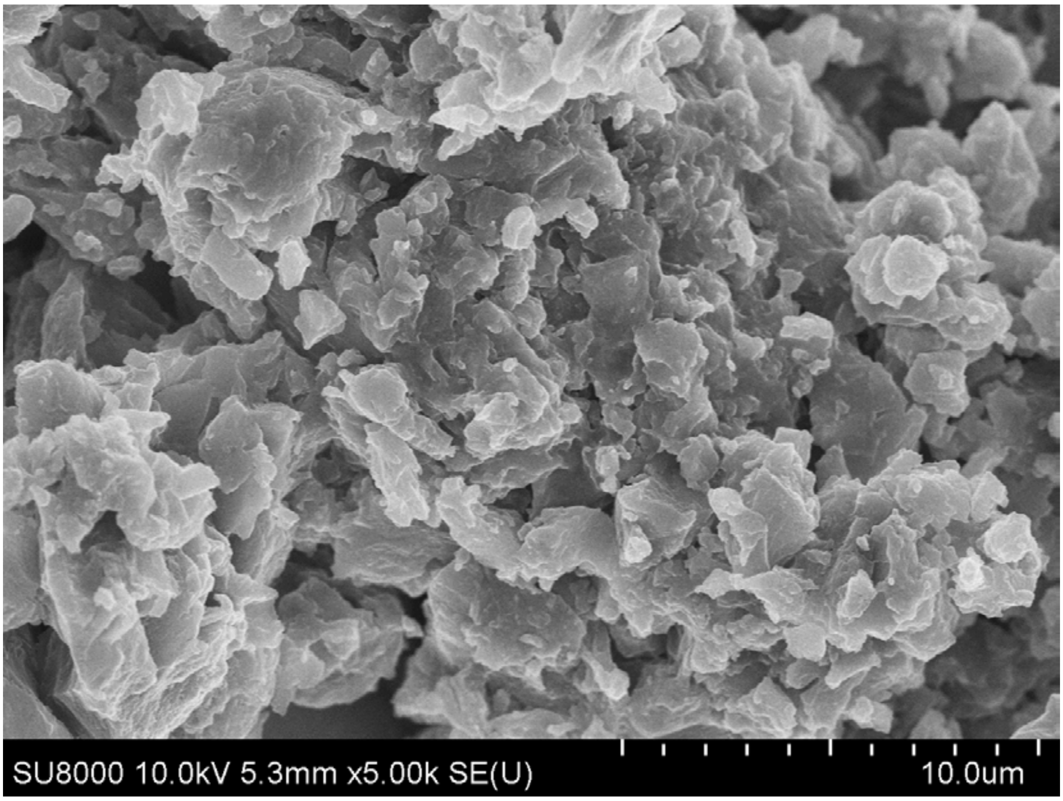

Supplementary Figure 41. FE-SEM image of $[Ni(SCH_2CH_2COOCH_3)_2]_n$ (**1l**) at x5000 magnification.

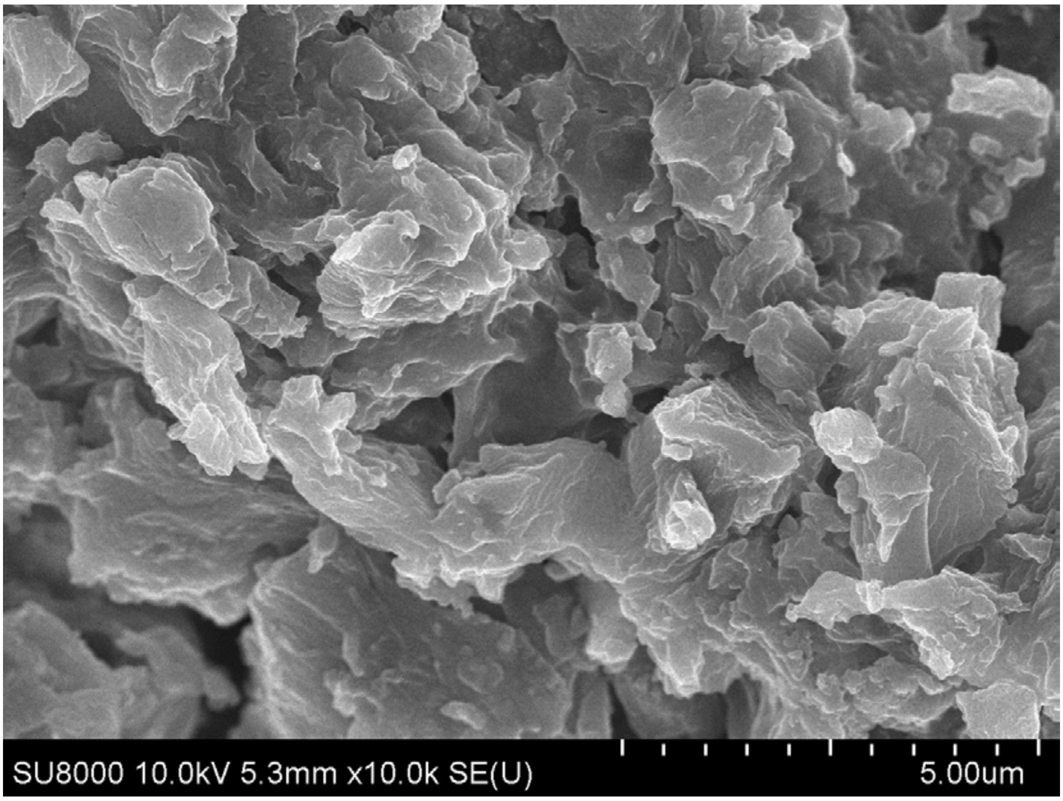

Supplementary Figure 42. FE-SEM image of $[Ni(SCH_2CH_2COOCH_3)_2]_n$ (**1l**) at x10000 magnification.

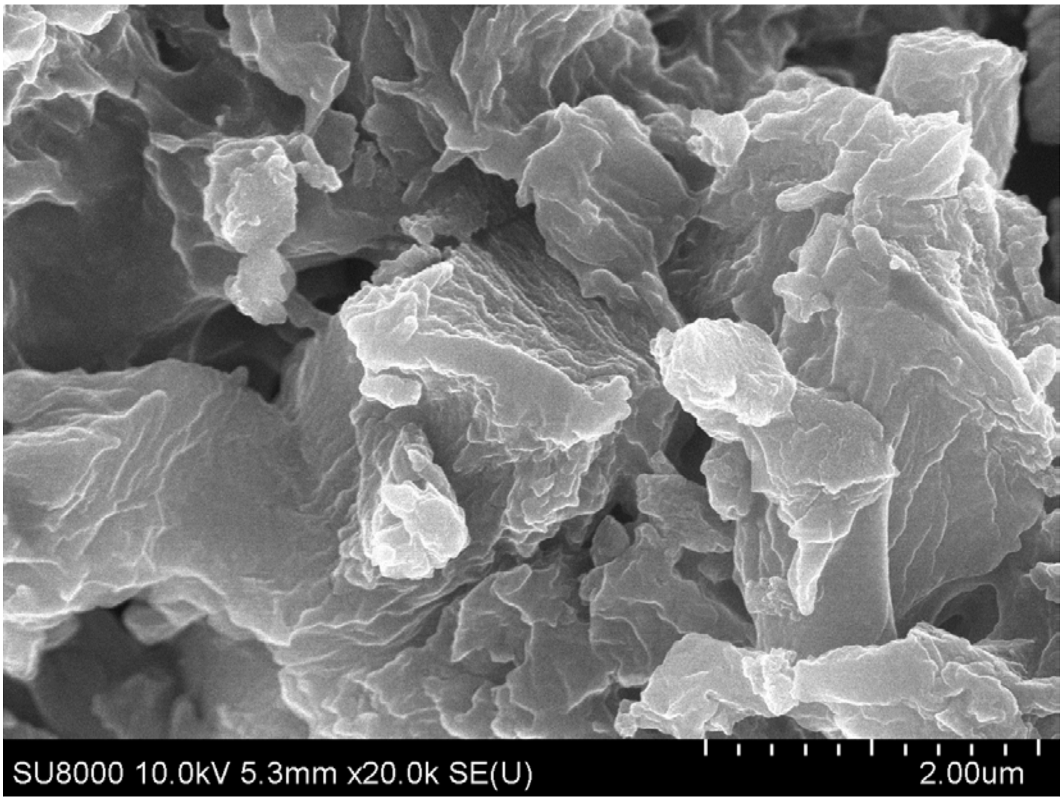

Supplementary Figure 43. FE-SEM image of $[Ni(SCH_2CH_2COOCH_3)_2]_n$ (**1l**) at x20000 magnification.

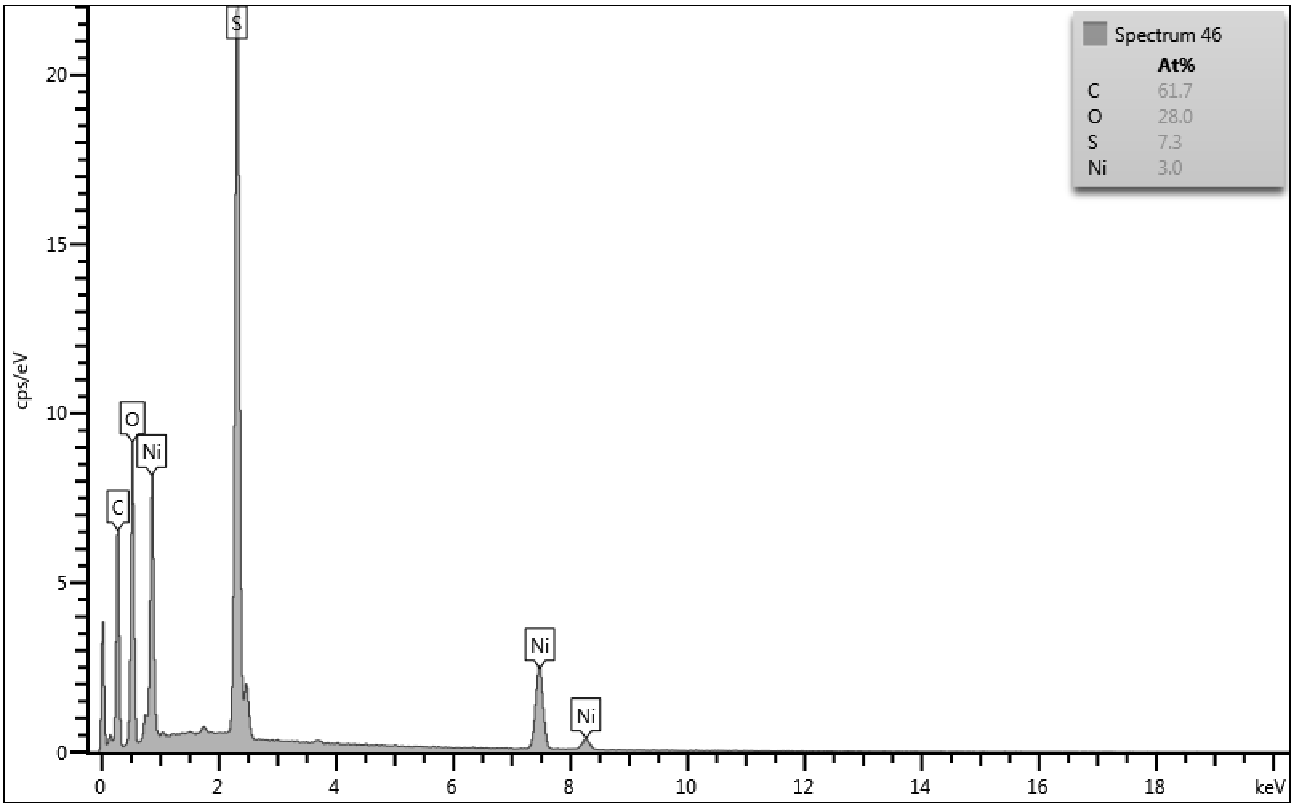

Supplementary Figure 44. EDX spectrum of $[Ni(SCH_2CH_2COOCH_3)_2]_n$ (**1l**).

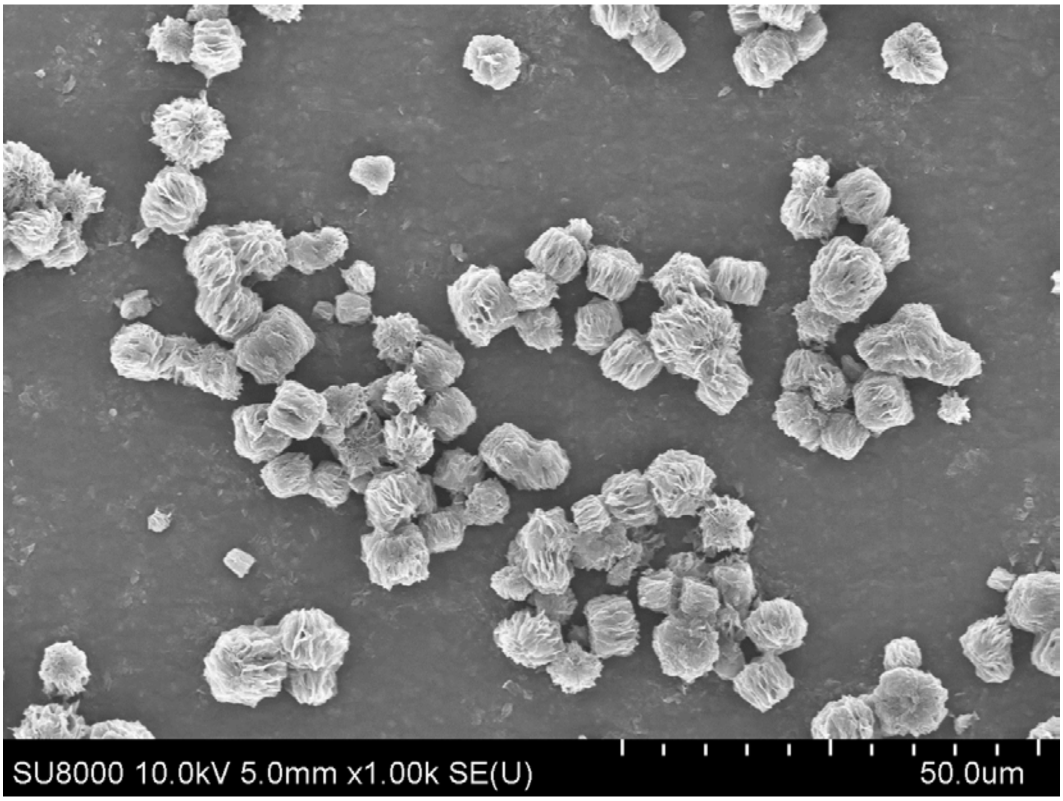

Supplementary Figure 45. FE-SEM image of $[Ni(S\textit{p}\text{-}FC_6H_4)_2]_n$ (**1m**) at x1000 magnification.

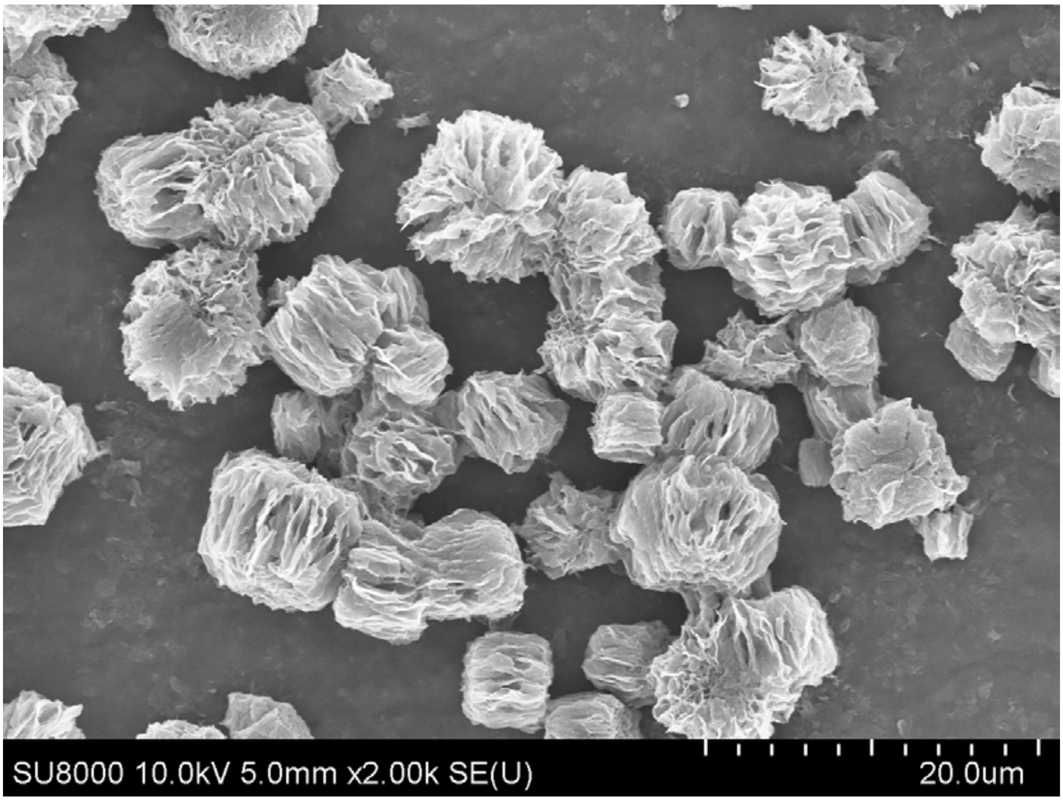

Supplementary Figure 46. FE-SEM image of $[Ni(S\textit{p}\text{-}FC_6H_4)_2]_n$ (**1m**) at x2000 magnification.

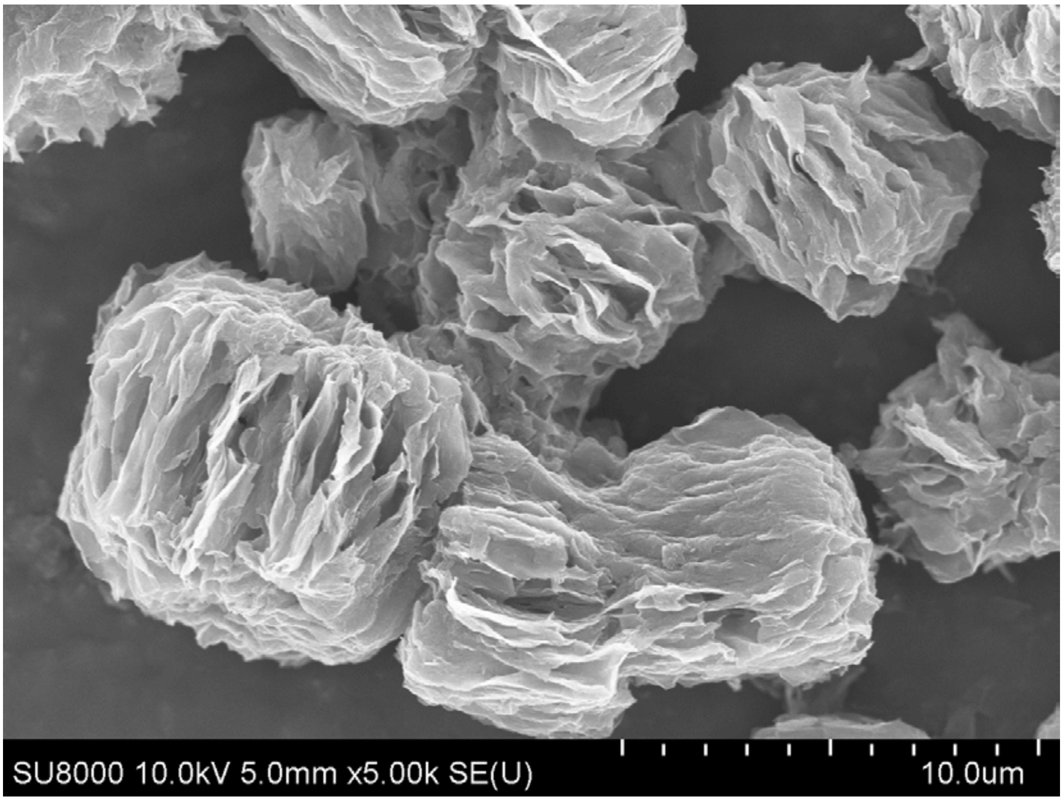

Supplementary Figure 47. FE-SEM image of $[Ni(S\textit{p}\text{-}FC_6H_4)_2]_n$ (**1m**) at x5000 magnification.

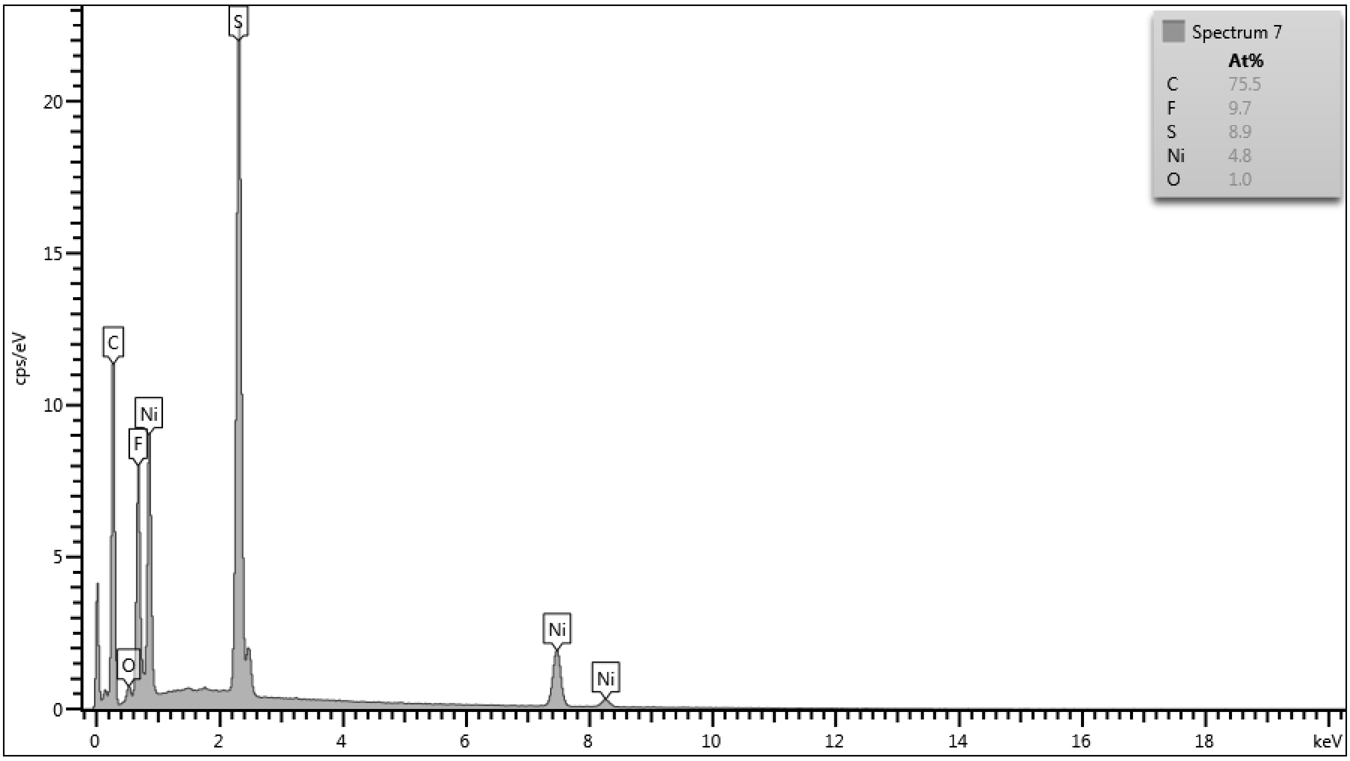

Supplementary Figure 48. EDX spectrum of $[Ni(S\textit{p}\text{-}FC_6H_4)_2]_n$ (**1m**).

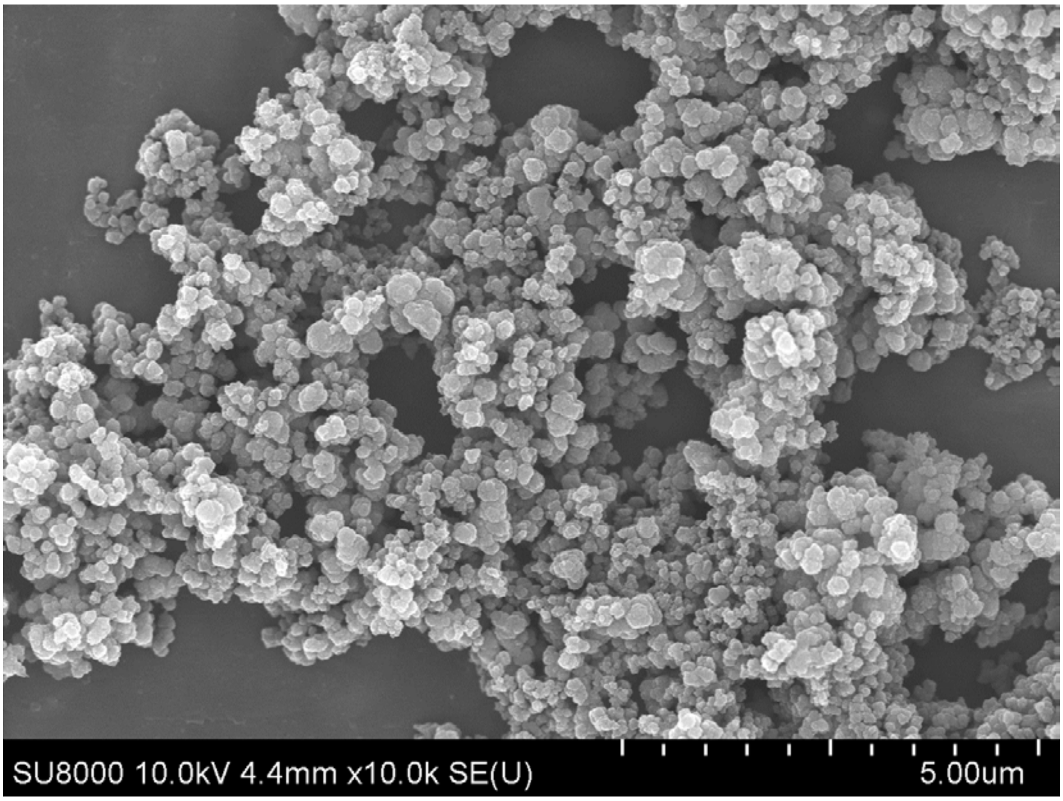

Supplementary Figure 49. FE-SEM image of $[Ni(SPh)_2]_n$ (**1a'**) at x10000 magnification.

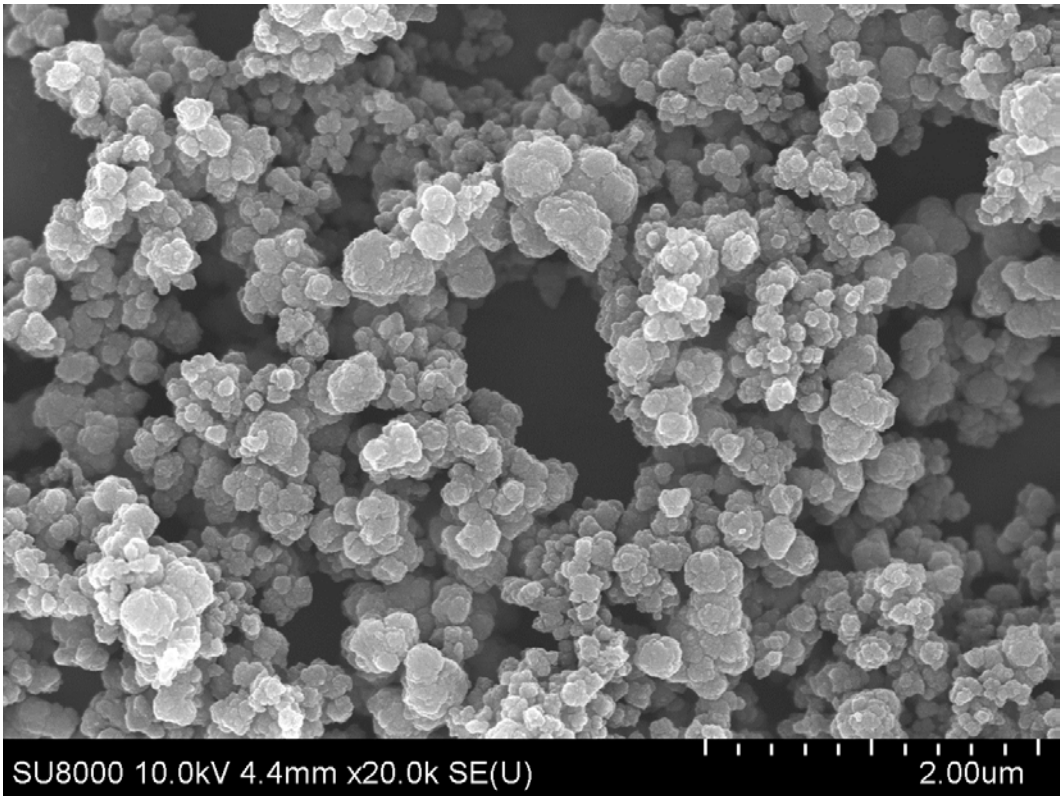

Supplementary Figure 50. FE-SEM image of $[Ni(SPh)_2]_n$ (**1a'**) at x20000 magnification.

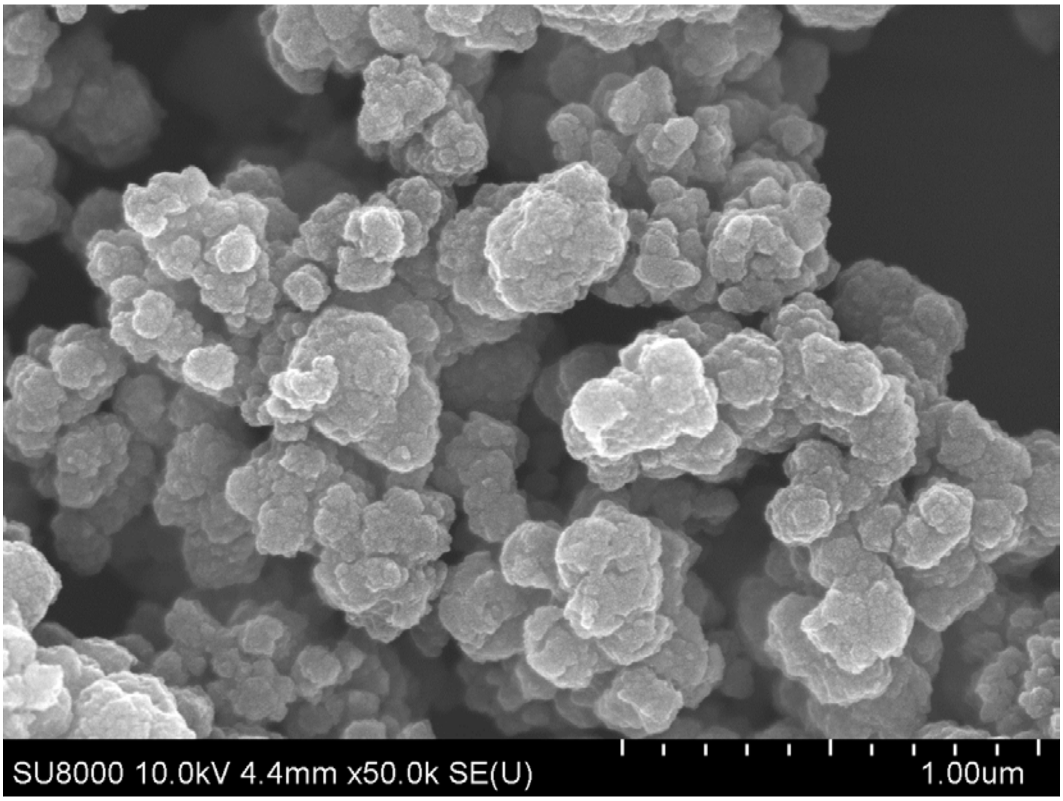

Supplementary Figure 51. FE-SEM image of $[Ni(SPh)_2]_n$ (**1a'**) at x50000 magnification.

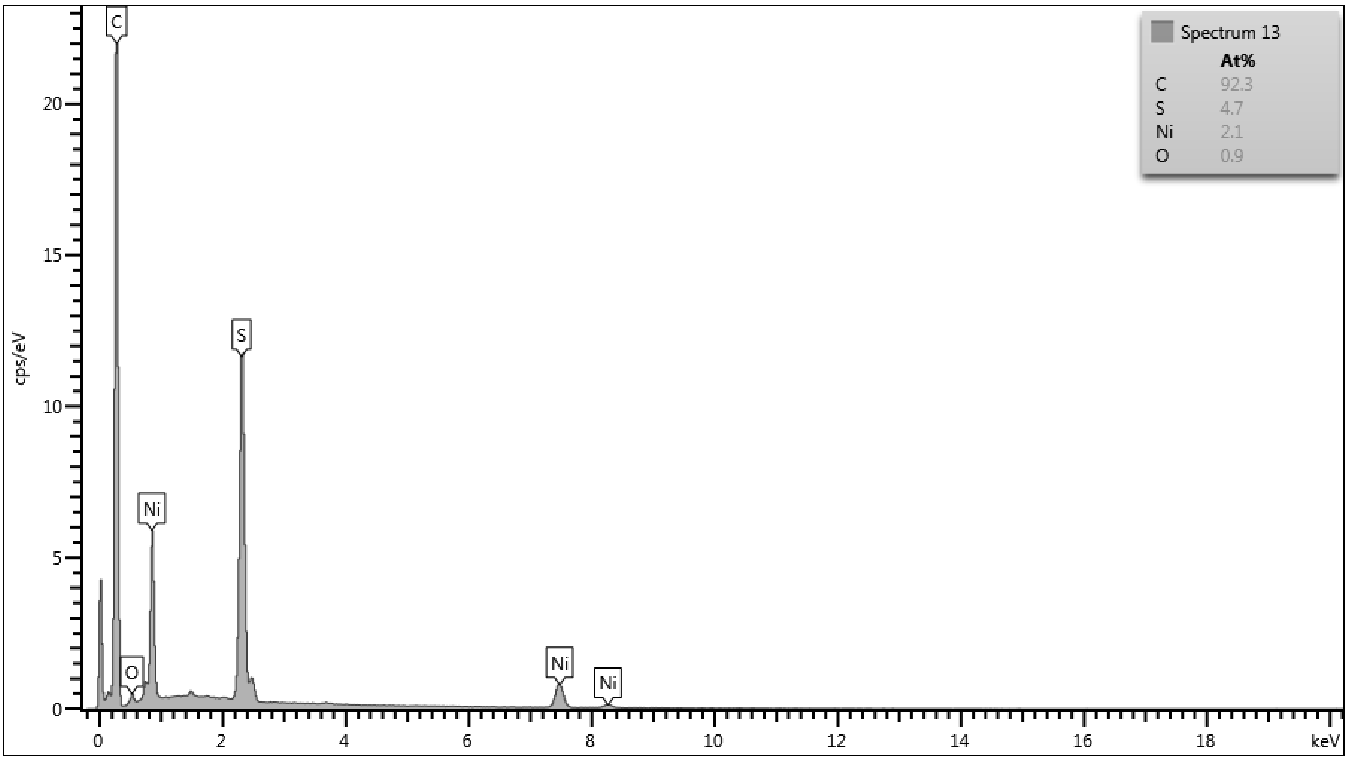

Supplementary Figure 52. EDX spectrum of $[Ni(SPh)_2]_n$ (**1a'**).

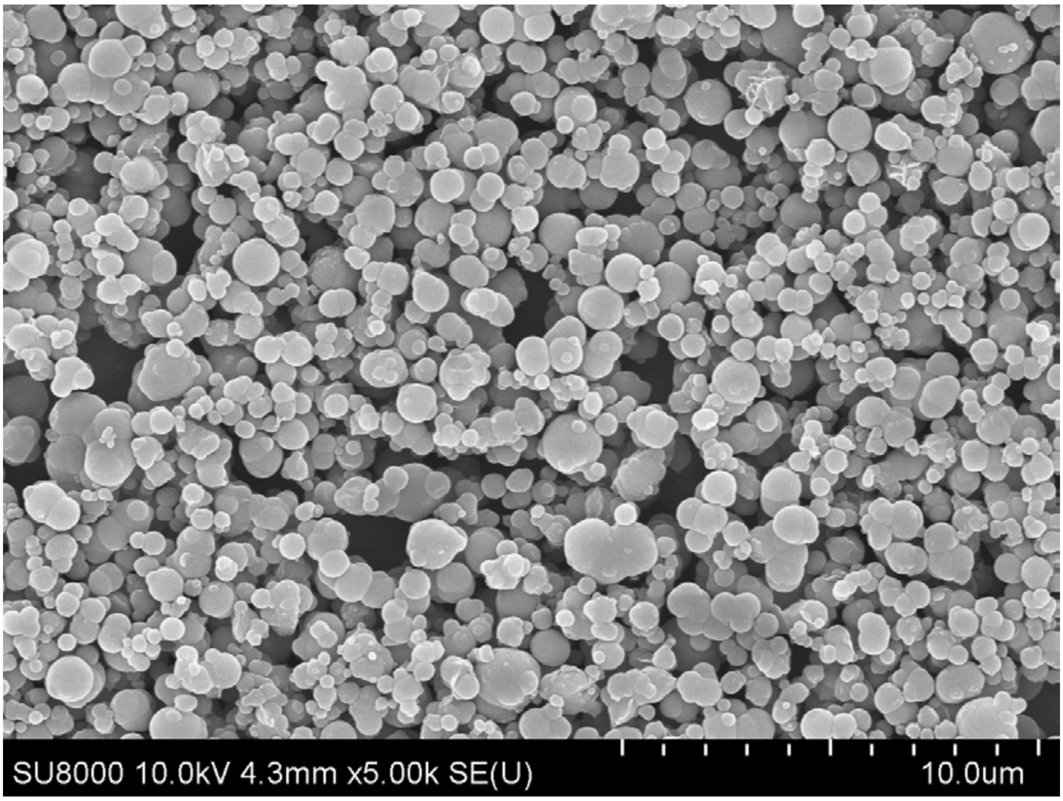

Supplementary Figure 53. FE-SEM image of $[Ni(SPh)_2]_n$ (**1a''**) at x5000 magnification.

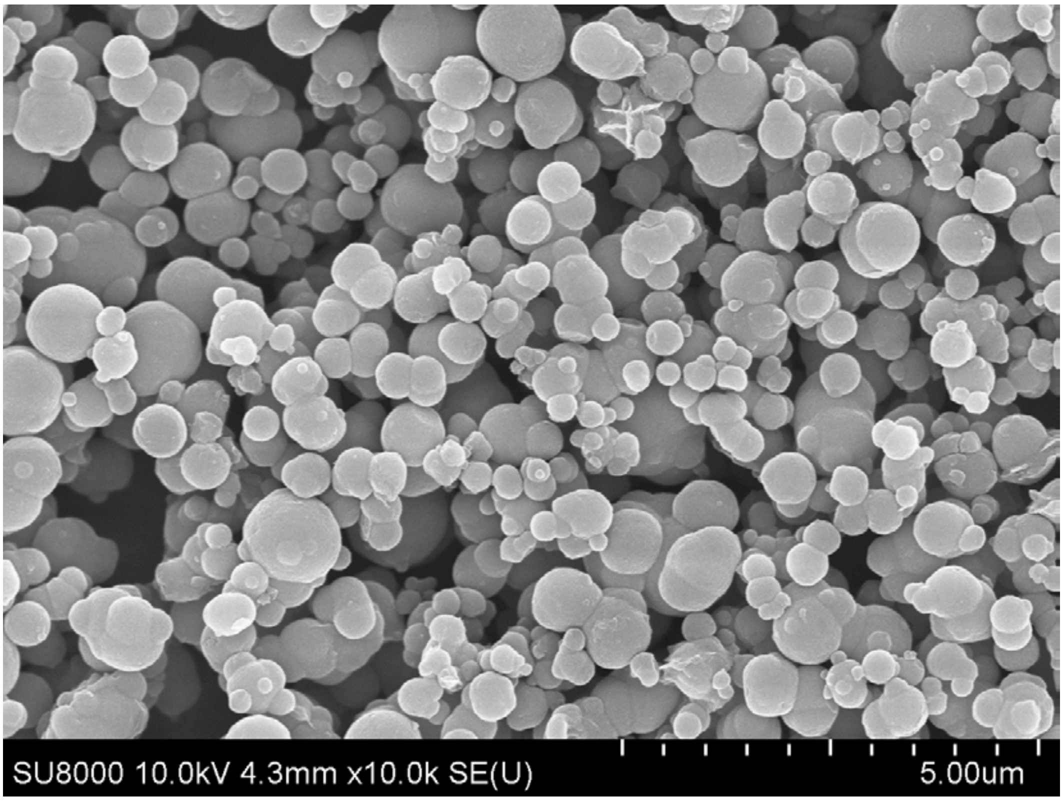

Supplementary Figure 54. FE-SEM image of $[Ni(SPh)_2]_n$ (**1a''**) at x10000 magnification.

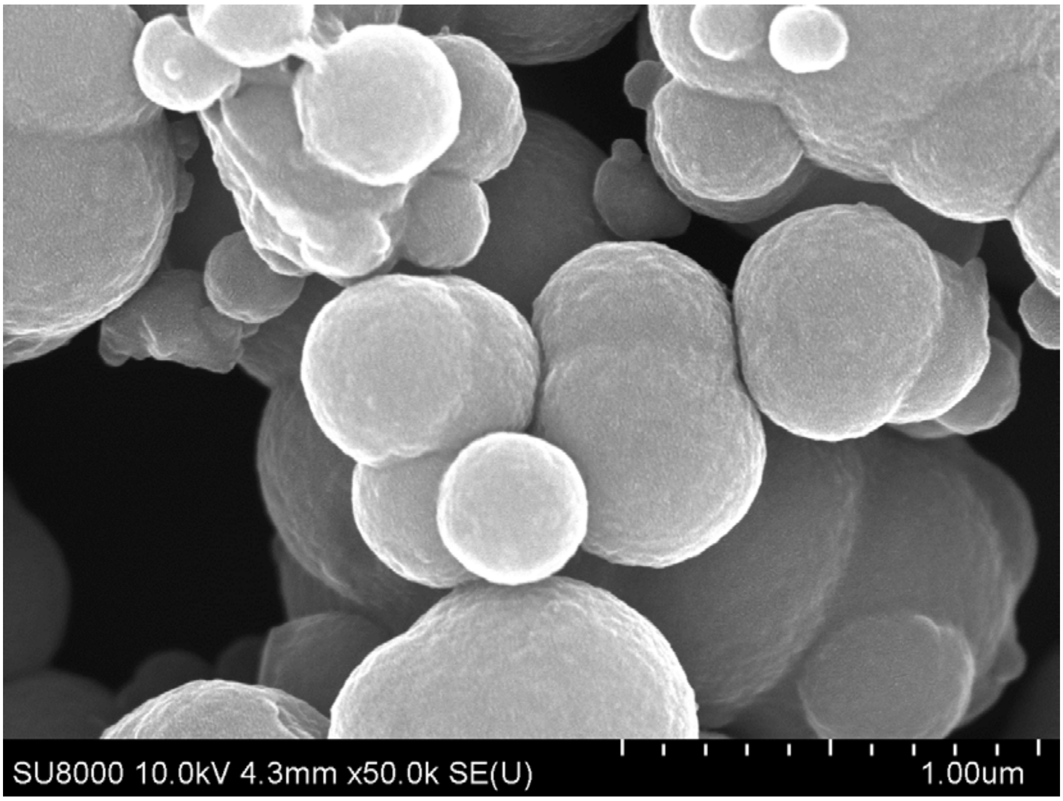

Supplementary Figure 55. FE-SEM image of $[Ni(SPh)_2]_n$ (**1a''**) at x50000 magnification.

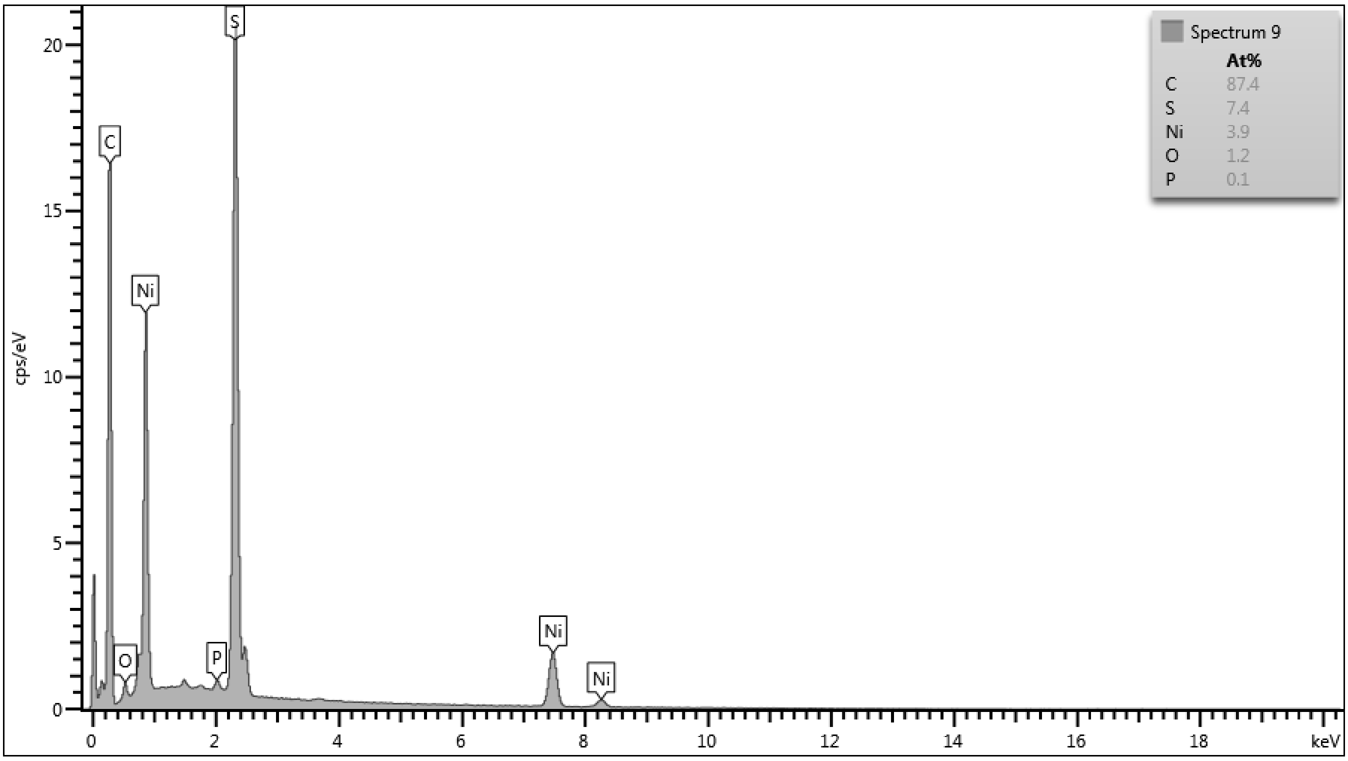

Supplementary Figure 56. EDX spectrum of $[Ni(SPh)_2]_n$ (**1a''**).

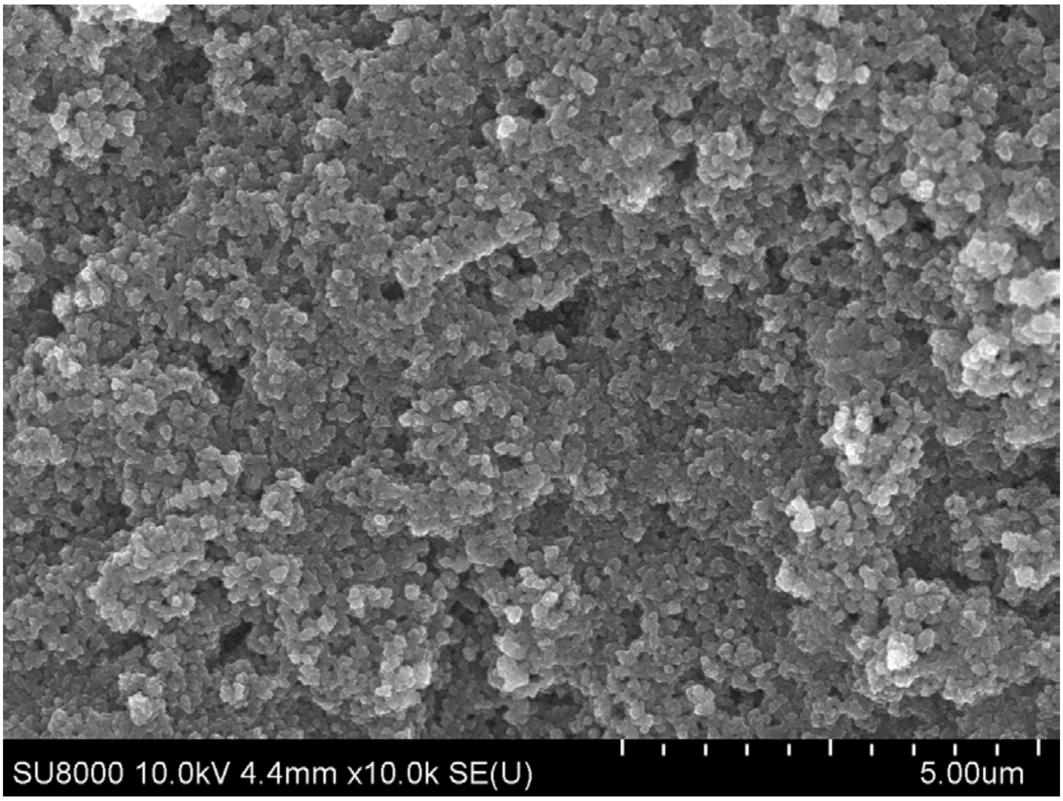

Supplementary Figure 57. FE-SEM image of [Ni(S*p*-BrC_6H_4)$_2$]$_n$ (**1b'**) at x10000 magnification.

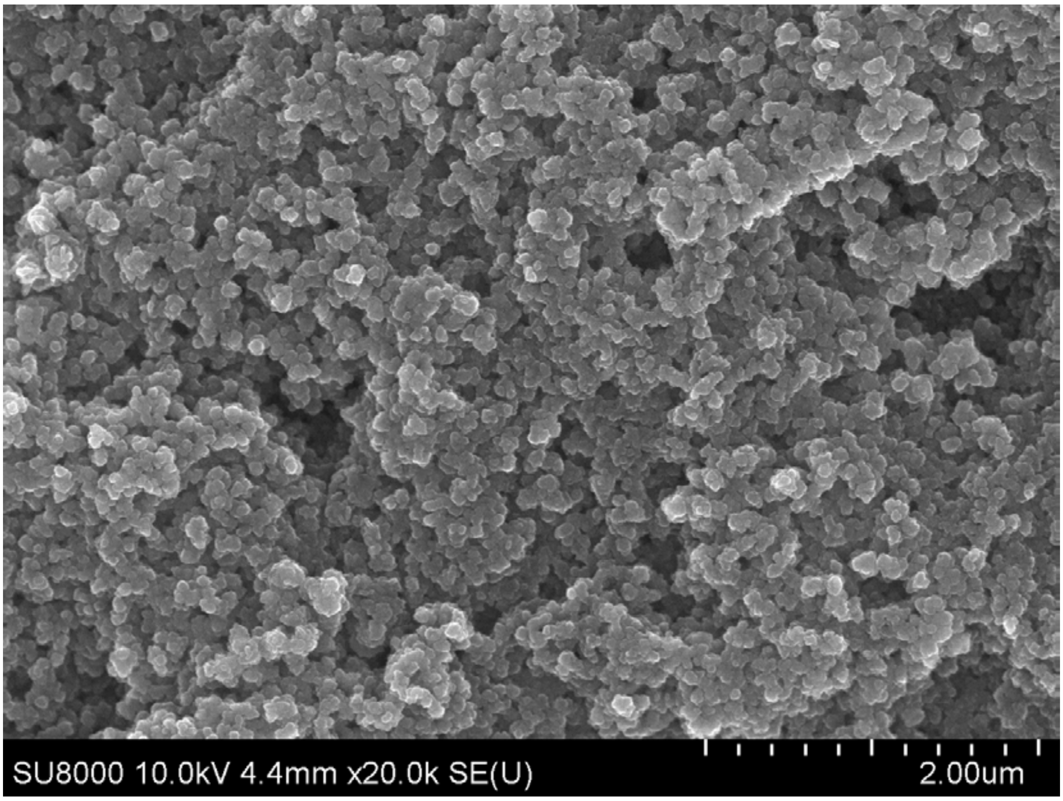

Supplementary Figure 58. FE-SEM image of [Ni(S*p*-BrC_6H_4)$_2$]$_n$ (**1b'**) at x20000 magnification.

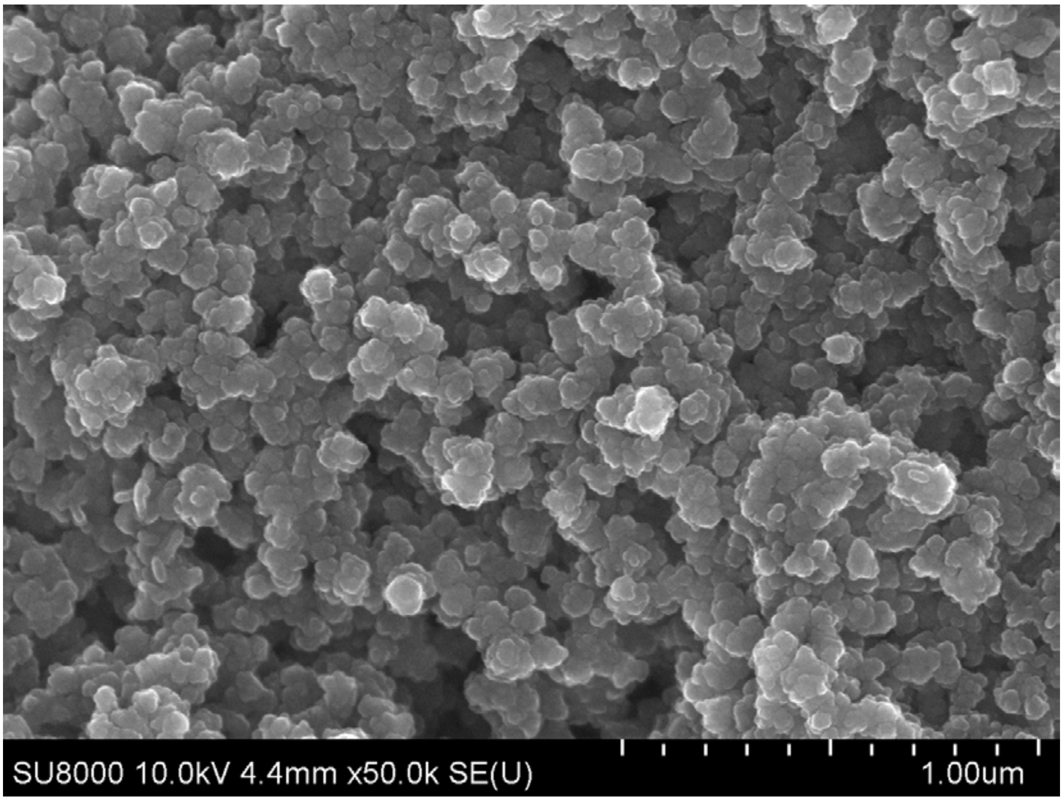

Supplementary Figure 59. FE-SEM image of $[Ni(Sp\text{-}BrC_6H_4)_2]_n$ (**1b'**) at x50000 magnification.

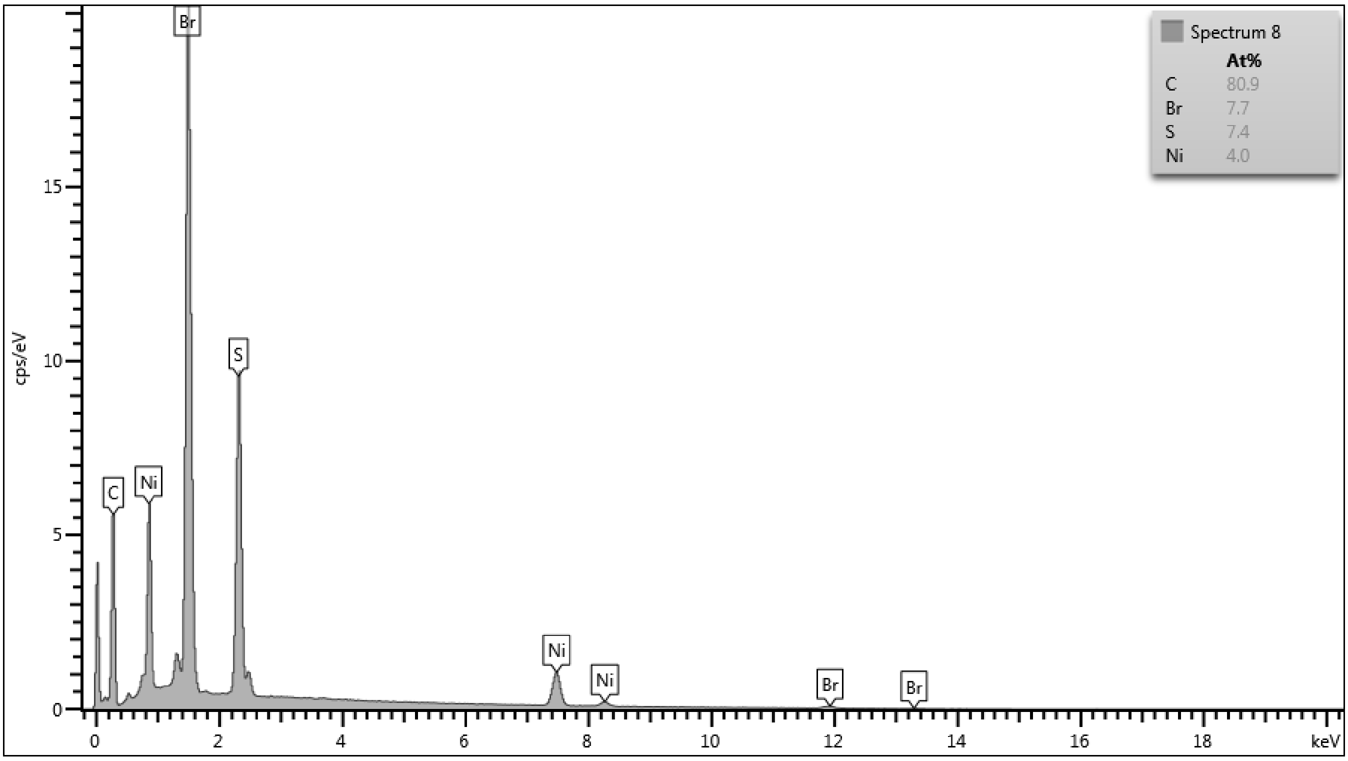

Supplementary Figure 60. EDX spectrum of $[Ni(Sp\text{-}BrC_6H_4)_2]_n$ (**1b'**).

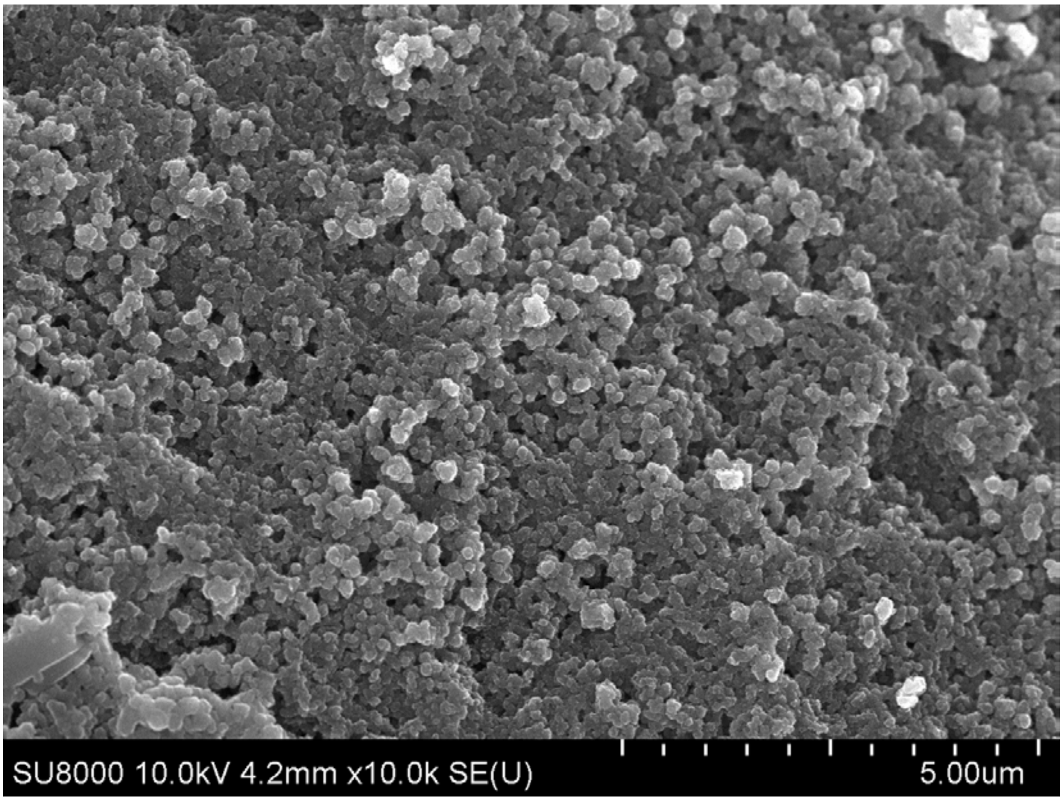

Supplementary Figure 61. FE-SEM image of [Ni(S*p*-ClC$_6$H$_4$)$_2$]$_n$ (**1g'**) at x10000 magnification.

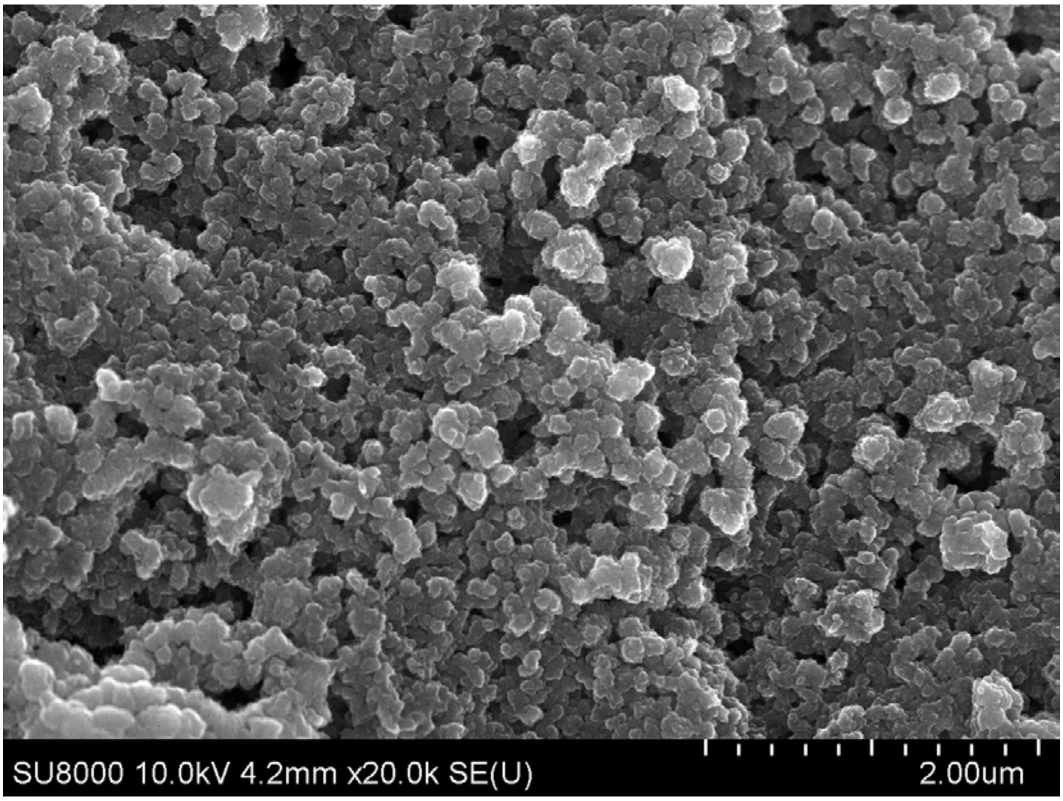

Supplementary Figure 62. FE-SEM image of [Ni(S*p*-ClC$_6$H$_4$)$_2$]$_n$ (**1g'**) at x20000 magnification.

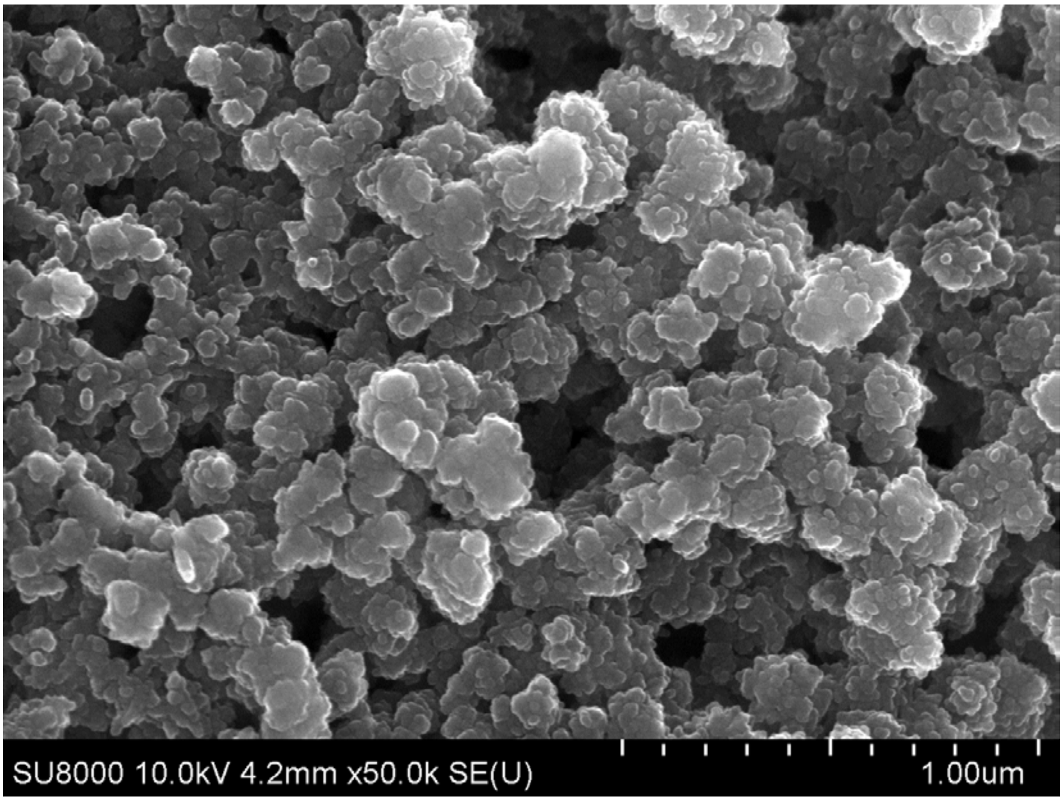

Supplementary Figure 63. FE-SEM image of $[Ni(S\textit{p}\text{-}ClC_6H_4)_2]_n$ (**1g'**) at x50000 magnification.

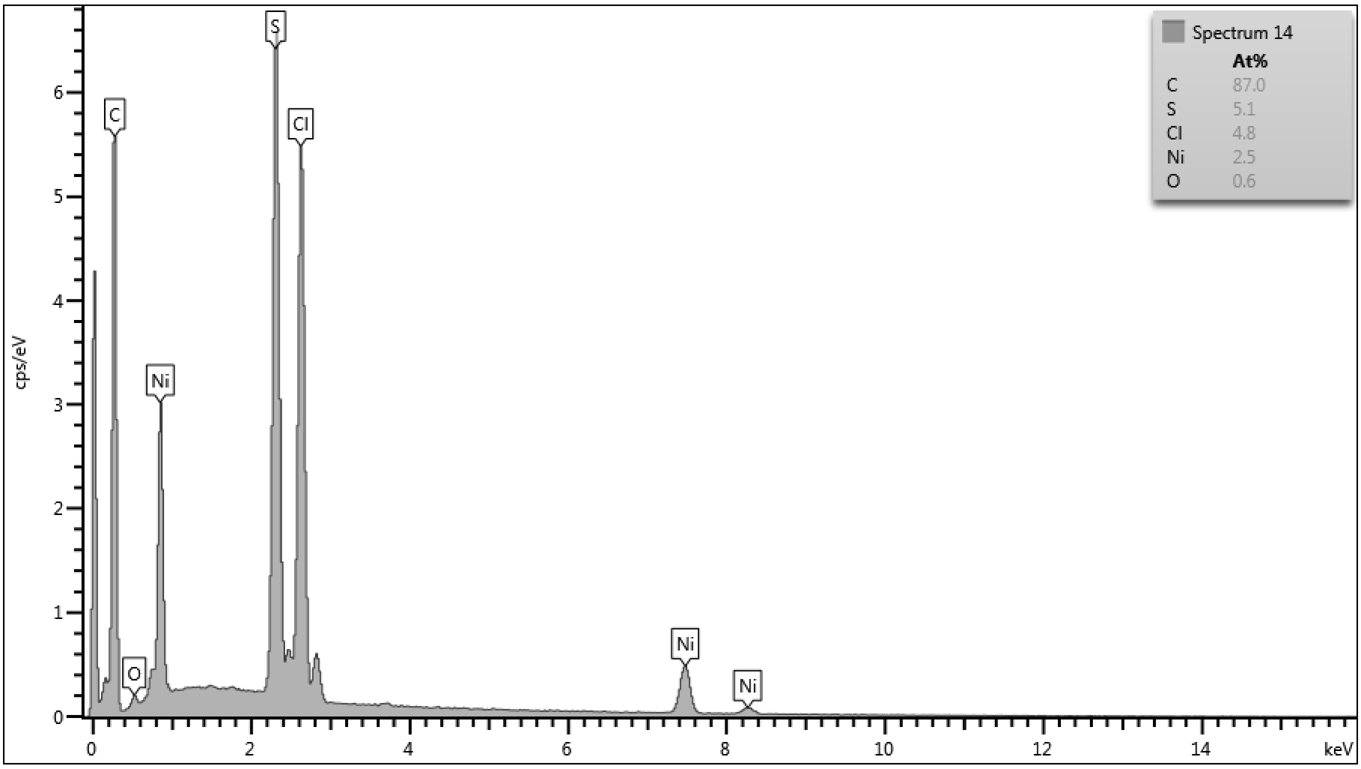

Supplementary Figure 64. EDX spectrum of $[Ni(S\textit{p}\text{-}ClC_6H_4)_2]_n$ (**1g'**).

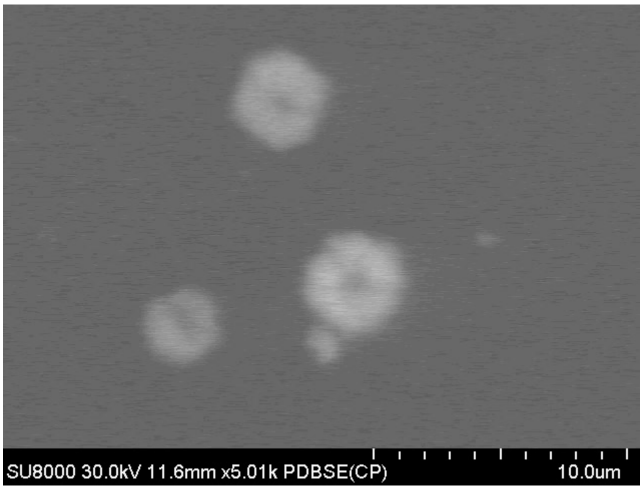

t = 0s

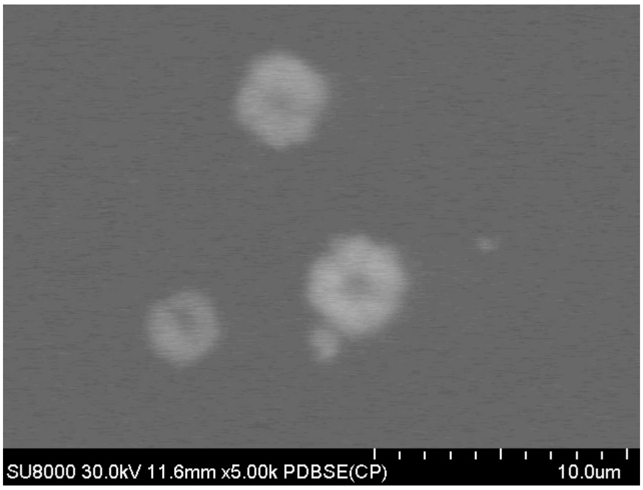

t = 35s

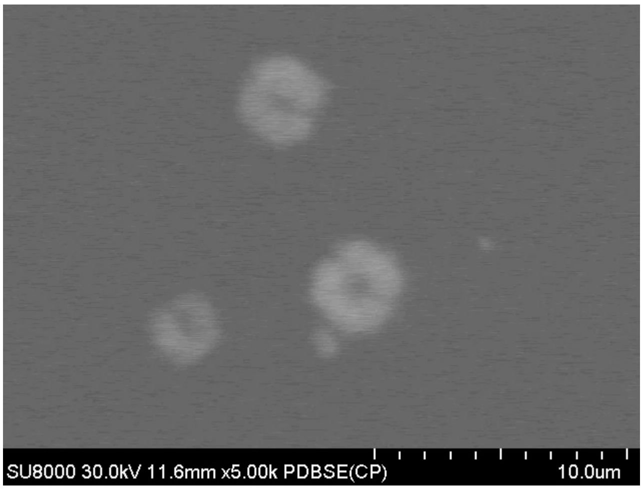

t = 70s

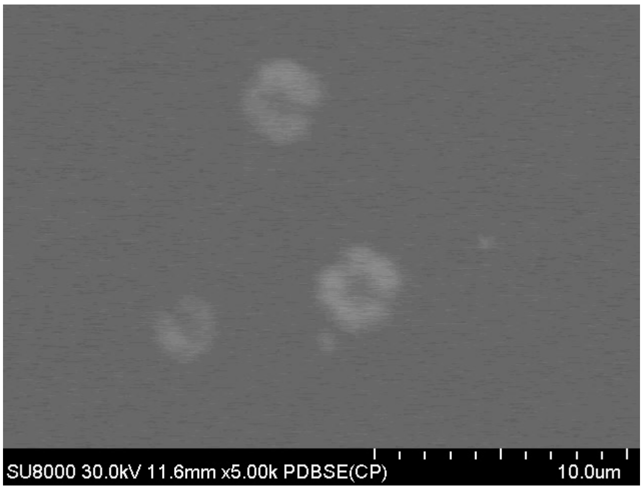

t = 105s

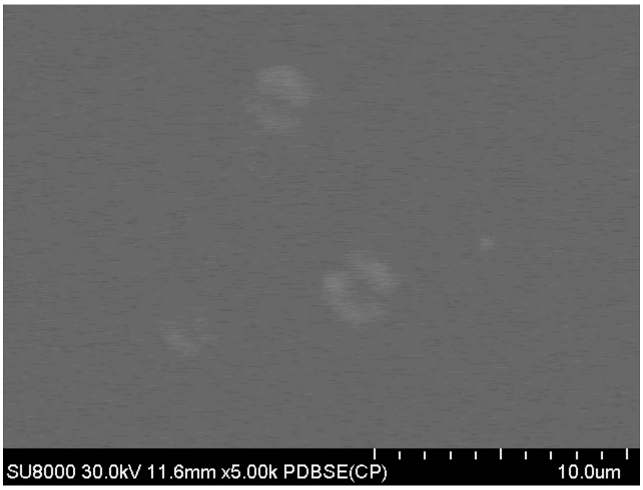

t = 140s

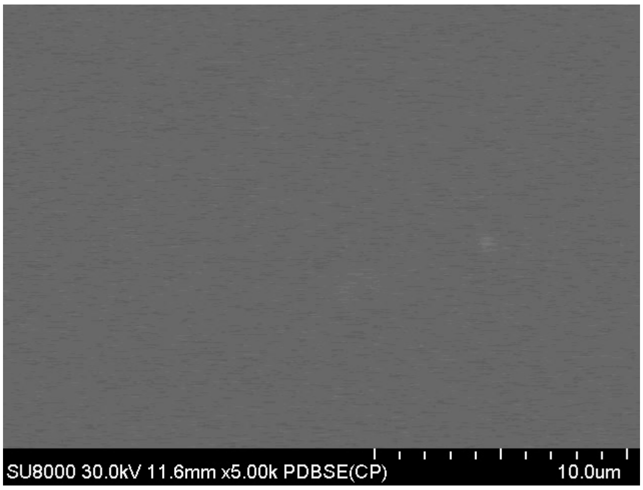

t = 175s

Supplementary Figure 65. Snapshots taken from SEM video of thiolate **1b** particles dissolution in $Pd(OAc)_2$/dppe/EtOH system.

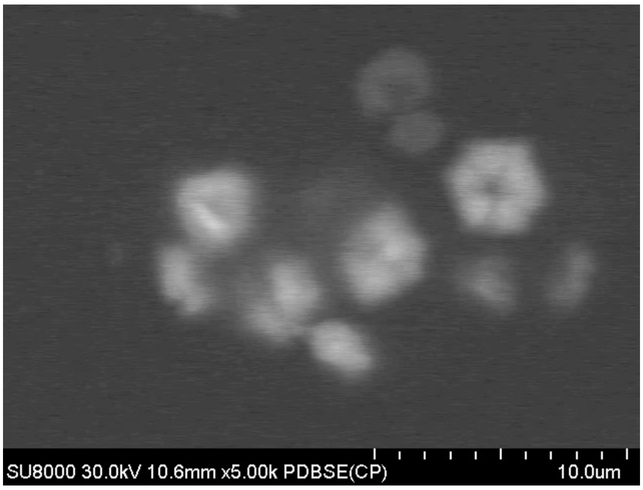

t = 0s

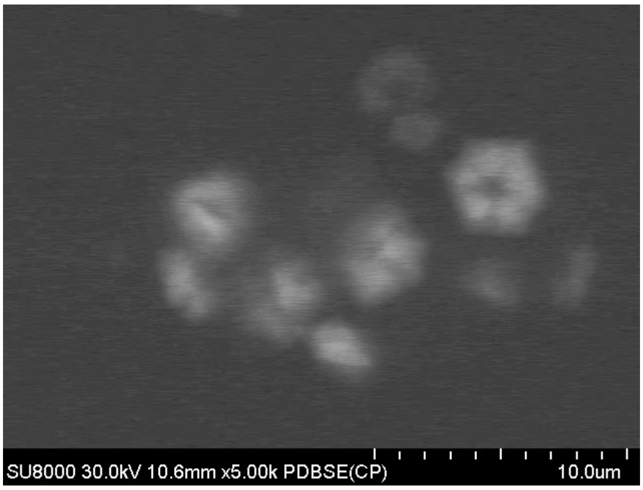

t = 5s

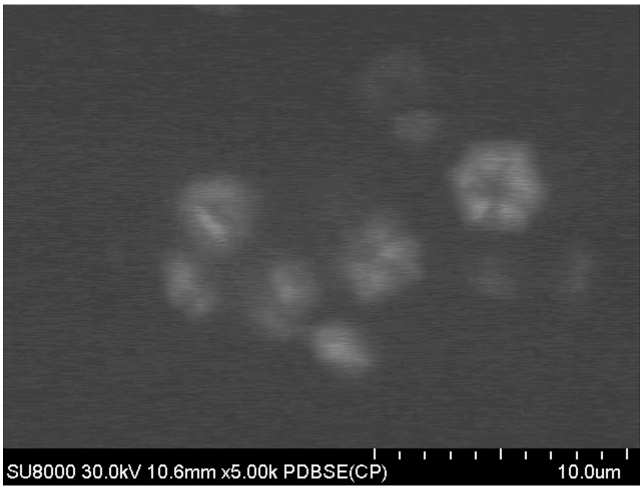

t = 10s

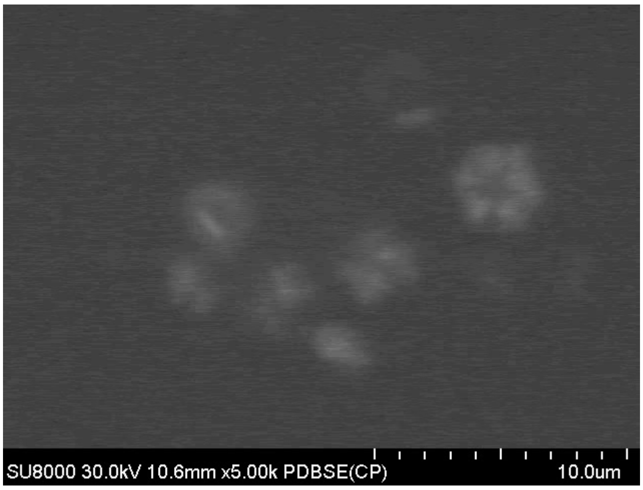

t = 15s

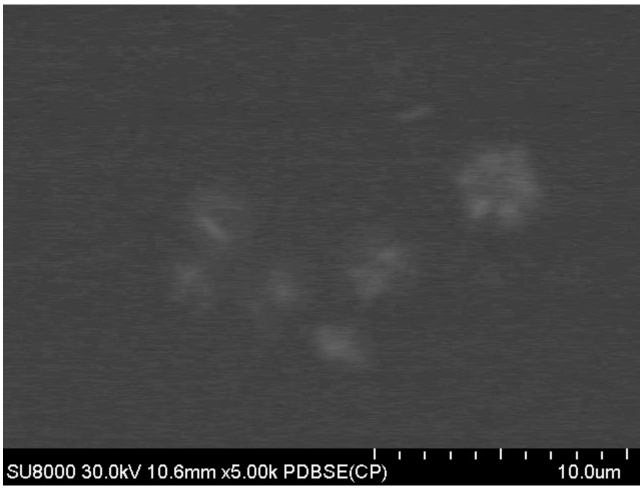

t = 20s

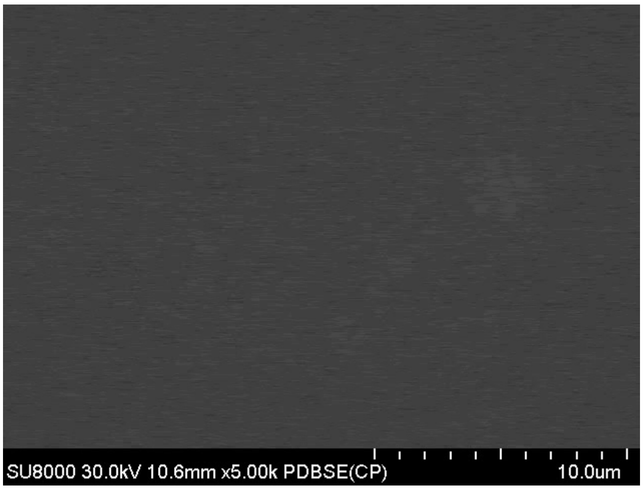

t = 35s

Supplementary Figure 66. Snapshots taken from SEM video of thiolate **1b** particles dissolution in $Pd(OAc)_2$/dppe/EtOH system in the presence of iodobenzene.

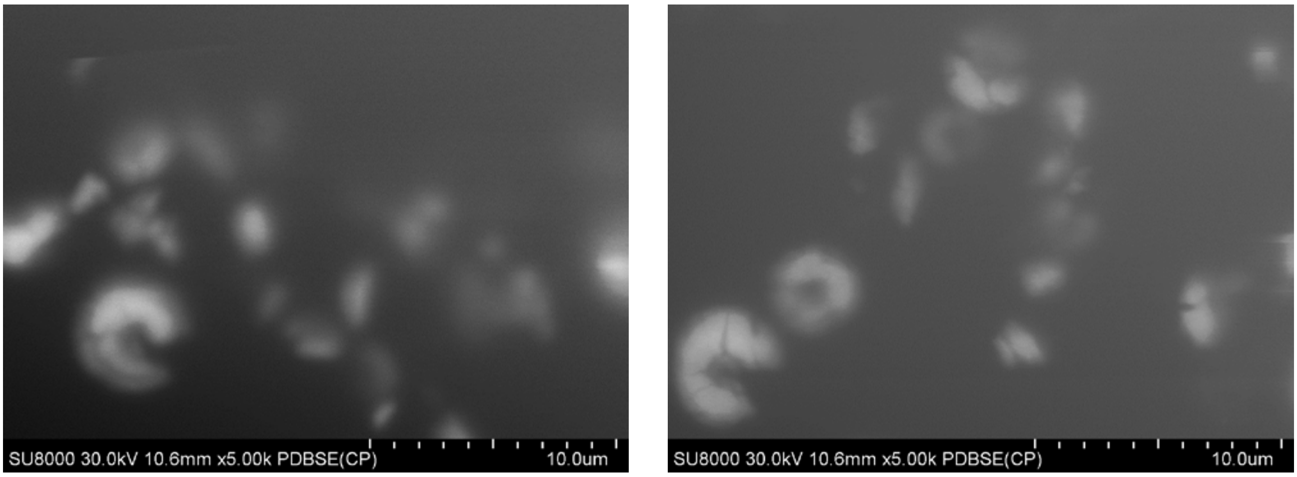

Supplementary Figure 67. Two representative FE-SEM images of the reaction mixture for the reaction between **1b** and iodobenzene after 45 minutes from the beginning of the reaction at x5000 magnification.

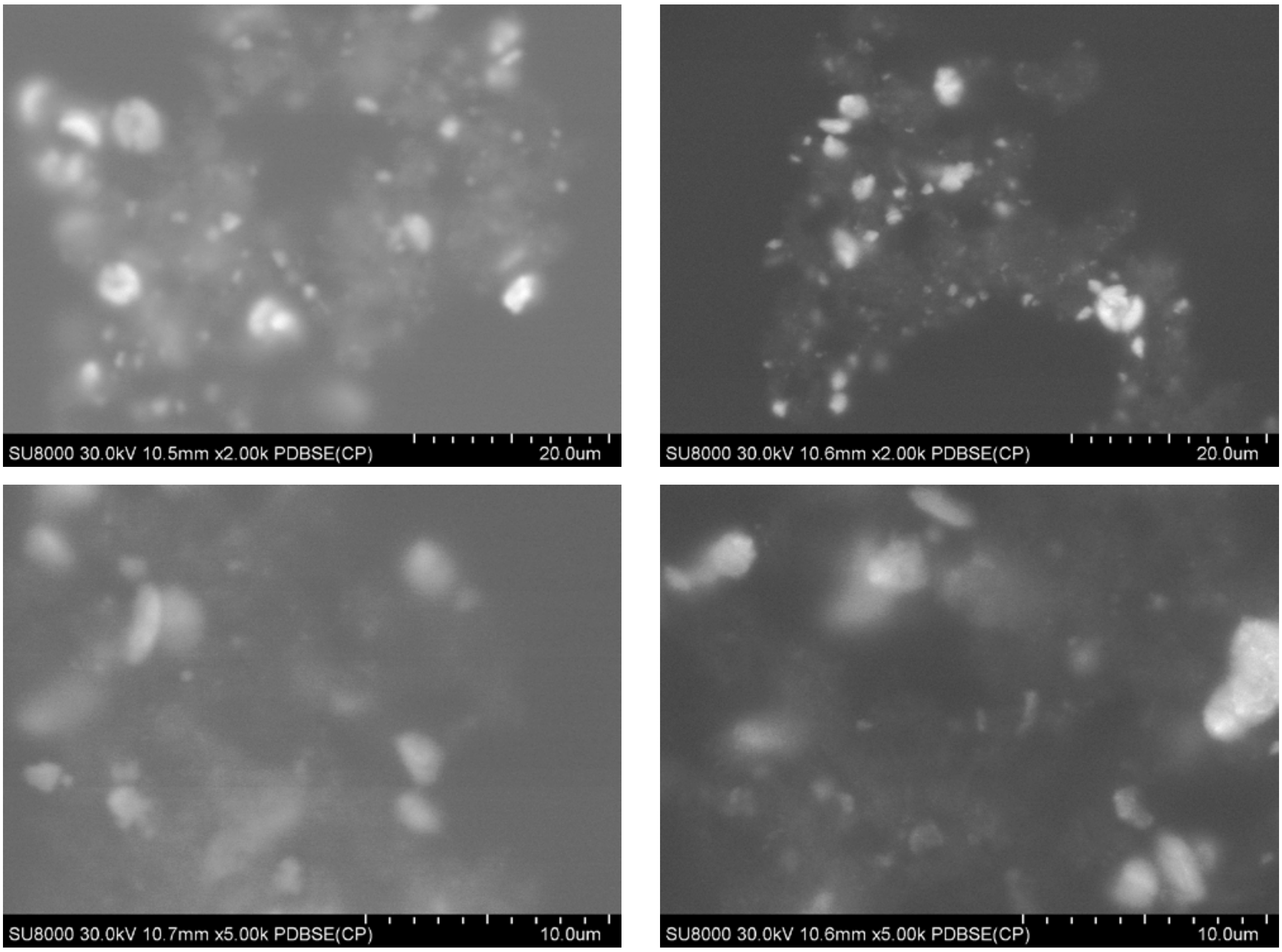

Supplementary Figure 68. Four representative FE-SEM images of the reaction mixture for the reaction between **1b** and iodobenzene after 2 hours from the beginning of the reaction at x2000 and x5000 magnifications.

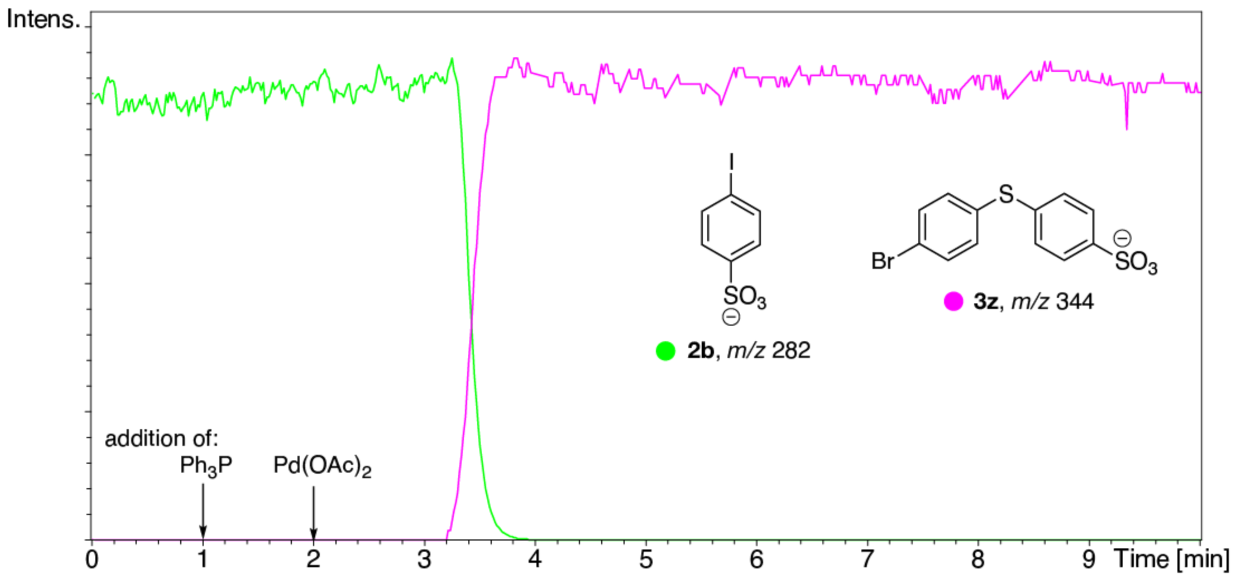

Supplementary Figure 69. The progress of the reaction between 4-iodobenzenesulpfonic acid (**2b**) and nickel thiolate **1b** observed in ESI MS on-line monitoring in negative ion mode.

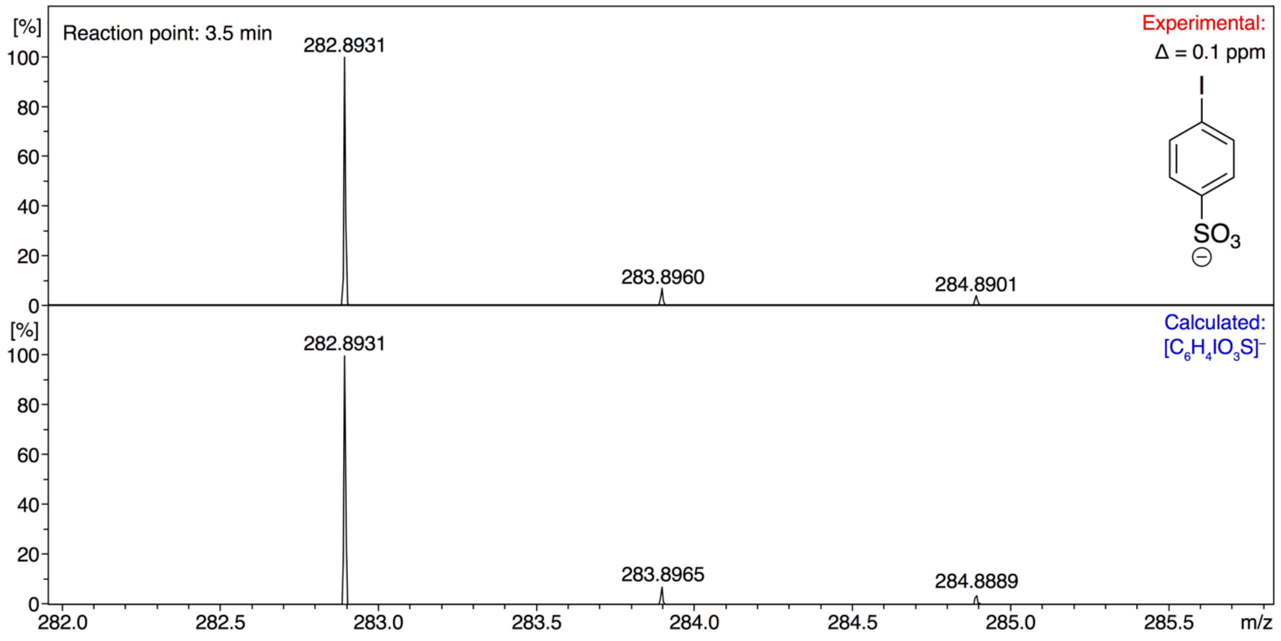

Supplementary Figure 70. Experimental ESI-(–)MS spectrum at 3.5 minutes of the monitored reaction between 4-iodobenzenesulpfonic acid (**2b**) and nickel thiolate **1b**, expanded to the reactant (**2b**) region. In the manuscript ion is denoted as *m/z* 282.

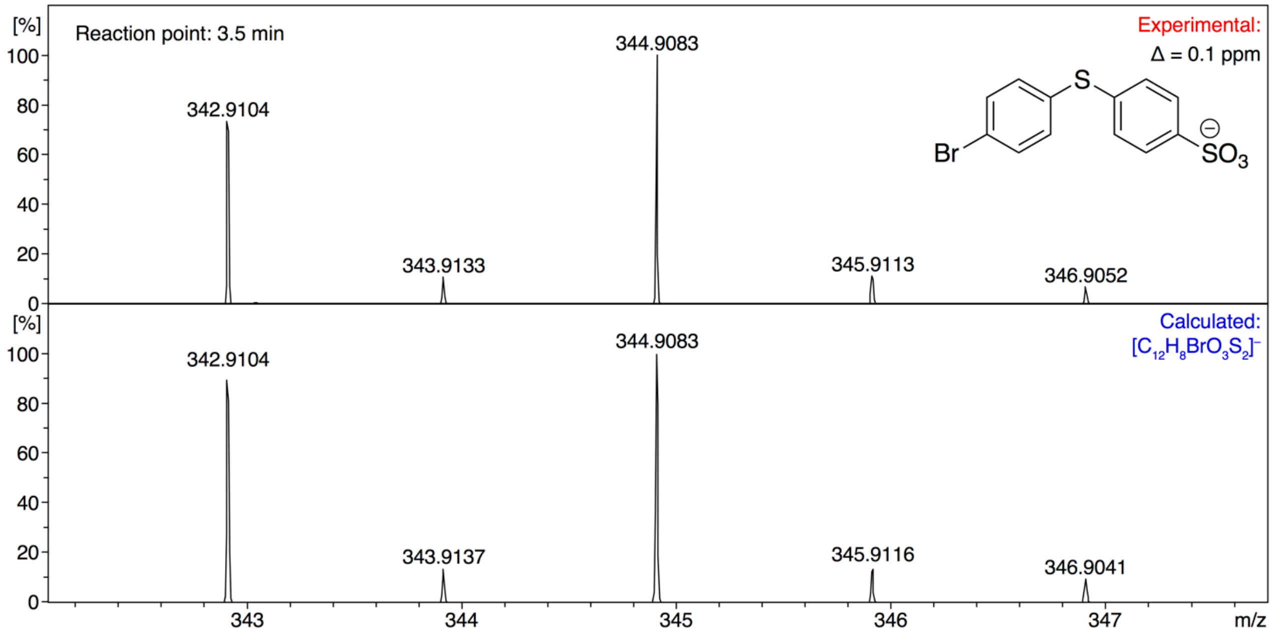

Supplementary Figure 71. Experimental ESI-(–)MS spectrum at 3.5 minutes of the monitored reaction between 4-iodobenzenesulpfonic acid (**2b**) and nickel thiolate **1b**, expanded to the product (**3z**) region. In the manuscript ion is denoted as *m/z* 344.

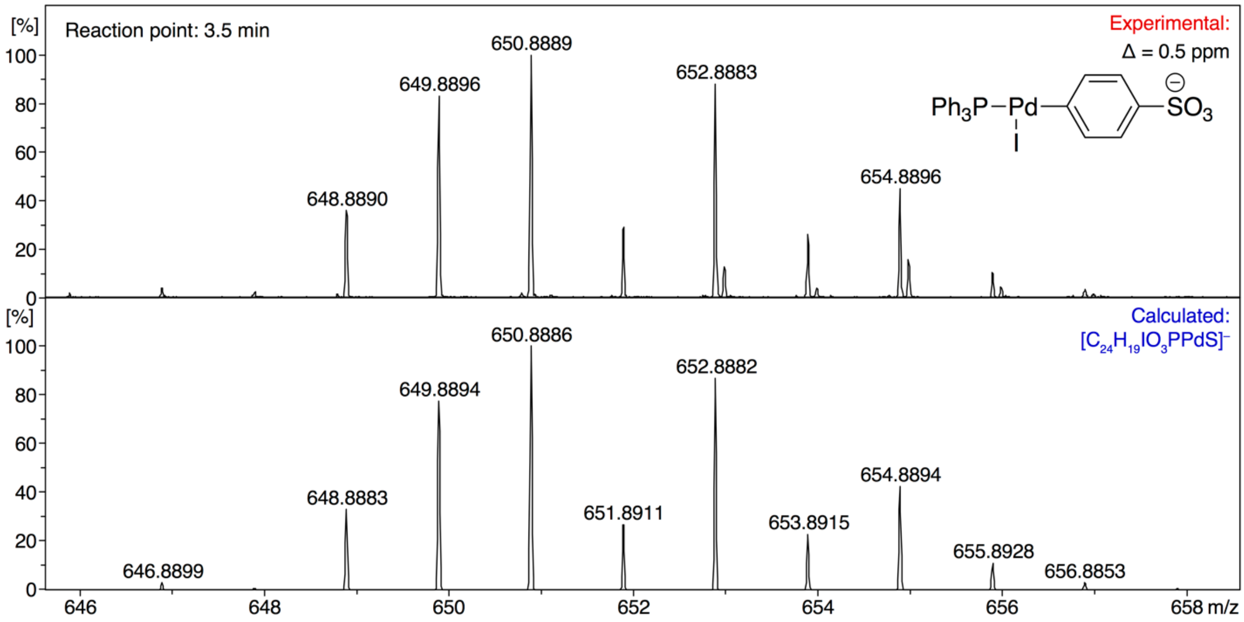

Supplementary Figure 72. Experimental ESI-(–)MS spectrum at 3.5 minutes of the monitored reaction between 4-iodobenzenesulpfonic acid (**2b**) and nickel thiolate **1b**, expanded to the intermediate **I** region. In the manuscript ion is denoted as *m/z* 650.

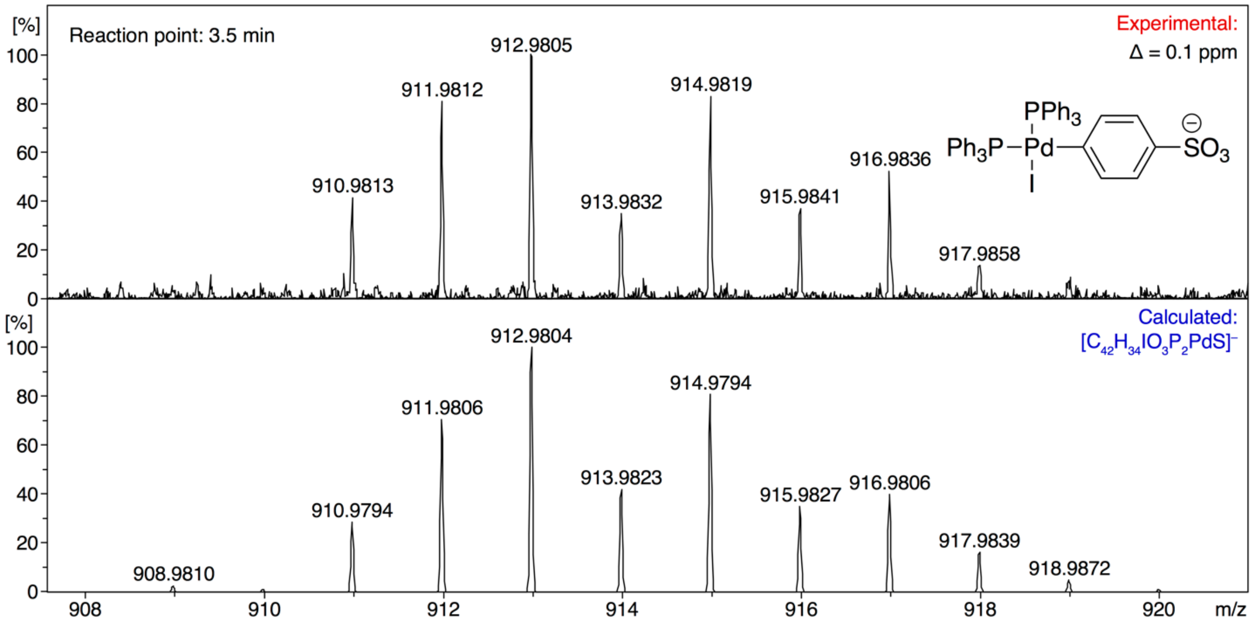

Supplementary Figure 73. Experimental ESI-(–)MS spectrum at 3.5 minutes of the monitored reaction between 4-iodobenzenesulpfonic acid (**2b**) and nickel thiolate **1b**, expanded to the intermediate **II** region. In the manuscript ion is denoted as *m/z* 912. In spite of low intensity, isotopic pattern was reproduced with reliable match and good accuracy was observed in exact and accurate masses (Δ = 0.1 ppm).

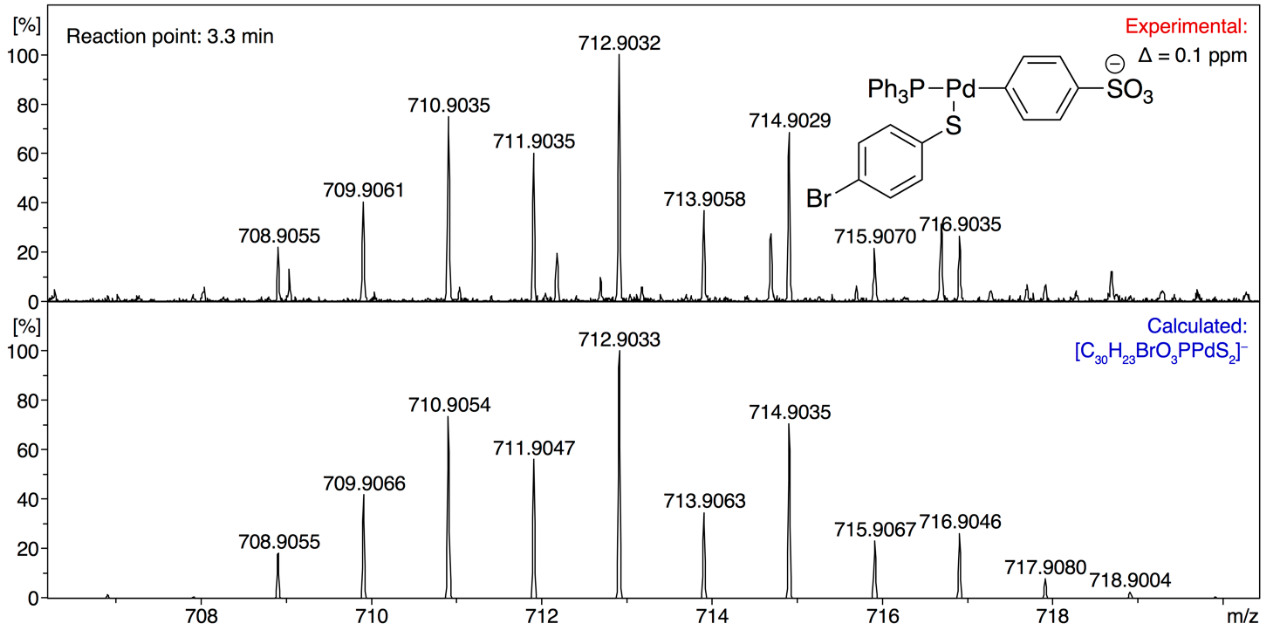

Supplementary Figure 74. Experimental ESI-(–)MS spectrum at 3.3 minutes of the monitored reaction between 4-iodobenzenesulpfonic acid (**2b**) and nickel thiolate **1b**, expanded to the intermediate **IV** region. In the manuscript ion is denoted as *m/z* 712. In spite of low intensity and signal interferences, isotopic pattern was reproduced with reliable match and good accuracy was observed in exact and accurate masses (Δ = 0.1 ppm).

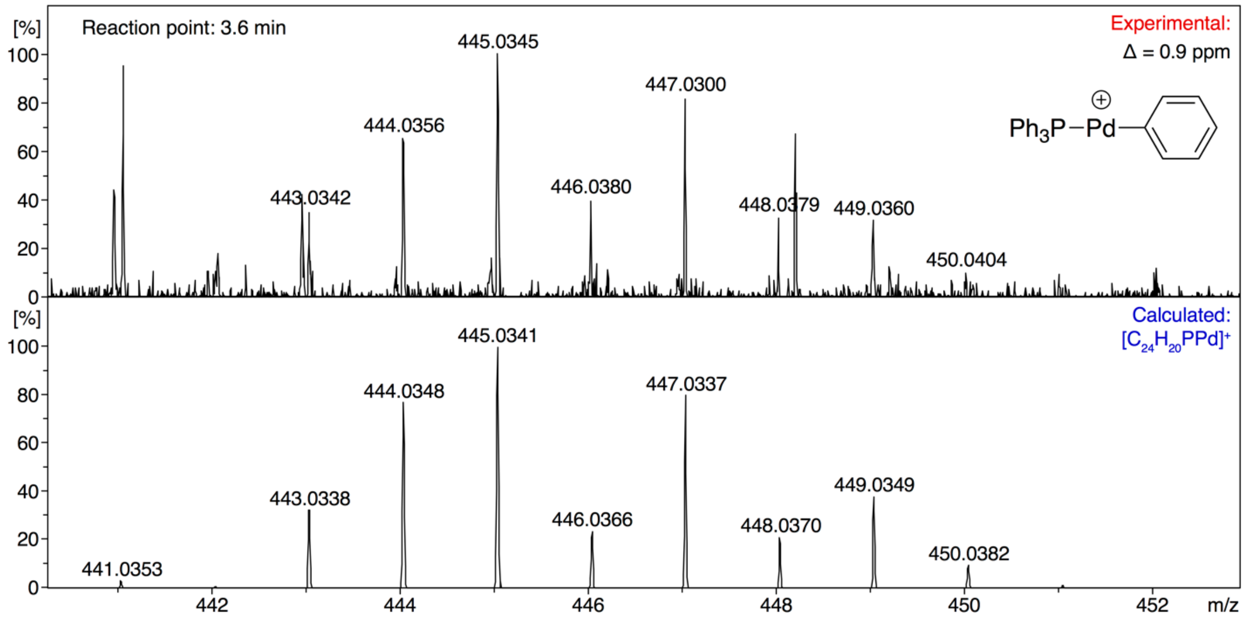

Supplementary Figure 75. Experimental ESI-(+)MS spectrum at 3.6 minutes of the monitored reaction between iodobenzene (**2a**) and nickel thiolate **1i**, expanded to the intermediate **I'** region. In the manuscript ion is denoted as *m/z* 445. Low signal-to-noise ratio was observed for these signals, which suggests only plausible identification of the complex. In spite of low intensity and signal interferences, isotopic pattern was reproduced with reliable match and good accuracy was observed in exact and accurate masses (Δ = 0.9 ppm).

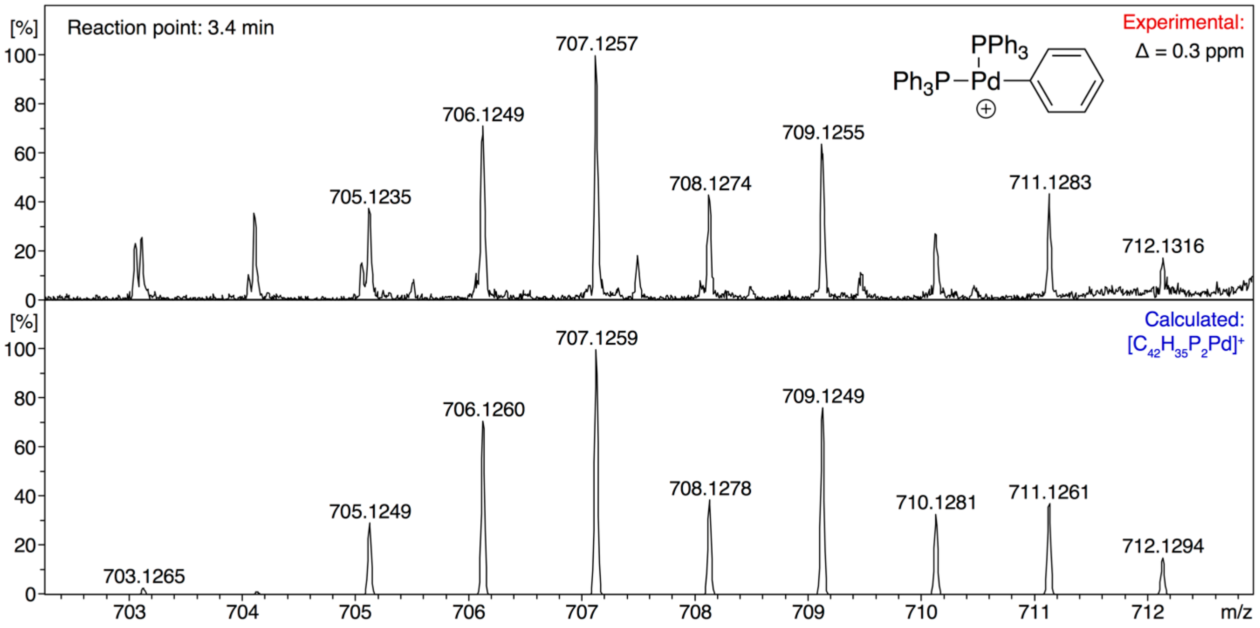

Supplementary Figure 76. Experimental ESI-(+)MS spectrum at 3.4 minutes of the monitored reaction between iodobenzene (**2a**) and nickel thiolate **1i**, expanded to the intermediate **II'** region. In the manuscript ion is denoted as *m/z* 707. In spite of low intensity and signal interferences, isotopic pattern was reproduced with reliable match and good accuracy was observed in exact and accurate masses (Δ = 0.3 ppm).

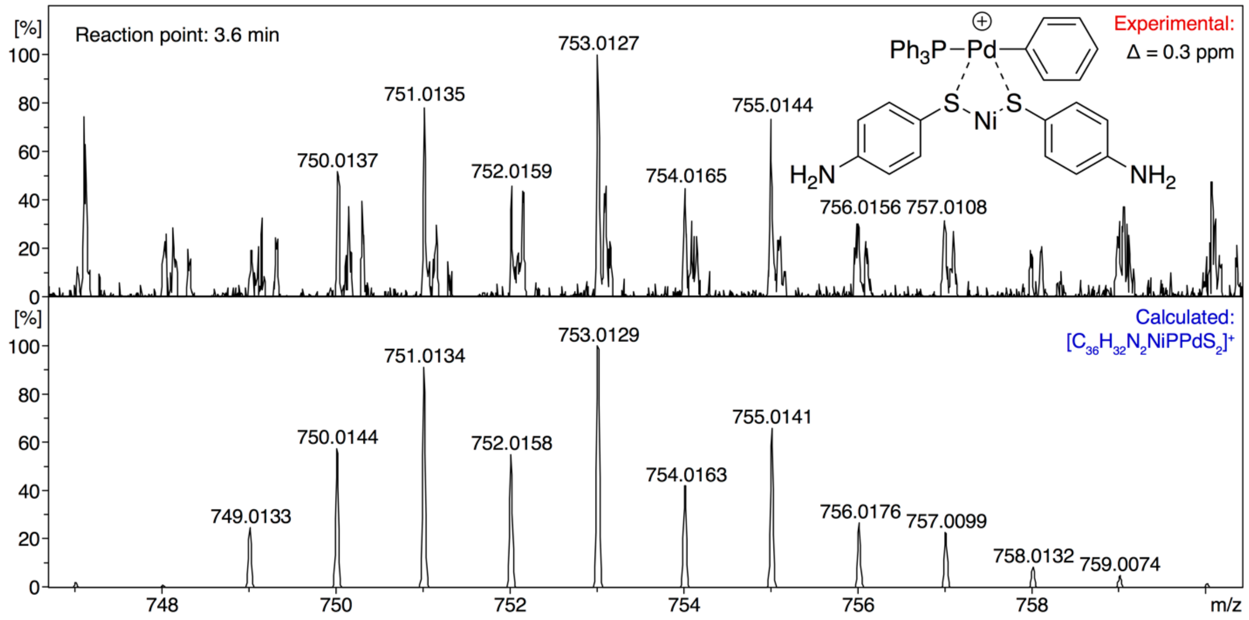

Supplementary Figure 77. Experimental ESI-(+)MS spectrum at 3.6 minutes of the monitored reaction between iodobenzene (**2a**) and nickel thiolate **1i**, expanded to the intermediate **III'** region. In the manuscript ion is denoted as *m/z* 753. Low signal-to-noise ratio was observed for these signals, which suggests only plausible identification of the complex. In spite of low intensity and signal interferences, isotopic pattern was reproduced with reliable match and good accuracy was observed in exact and accurate masses (Δ = 0.3 ppm).

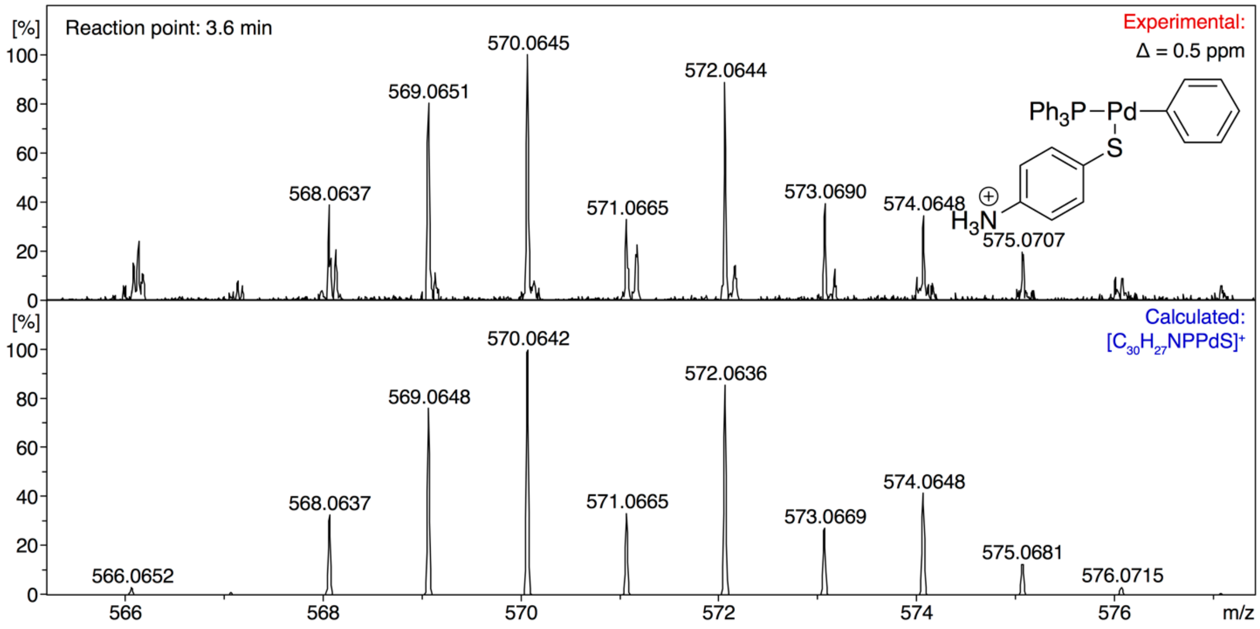

Supplementary Figure 78. Experimental ESI-(+)MS spectrum at 3.6 minutes of the monitored reaction between iodobenzene (**2a**) and nickel thiolate **1i**, expanded to the intermediate **IV'** region. In the manuscript ion is denoted as *m/z* 570. Low signal-to-noise ratio was observed for these signals, which suggests only plausible identification of the complex. In spite of low intensity and signal interferences, isotopic pattern was reproduced with reliable match and good accuracy was observed in exact and accurate masses (Δ = 0.5 ppm).

Supplementary Tables

Supplementary Table 1. C-S cross-coupling reaction between various aryl halides and nickel thiolates.

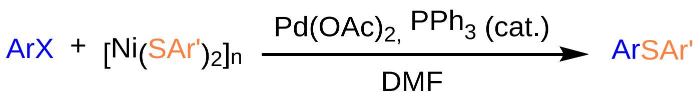

Entry	X	Reaction conditions	Product	Yield of ArSAr′, %
1	Br	120°C, 24h	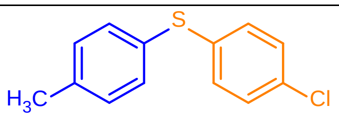	58
2	Br	120°C, 24h	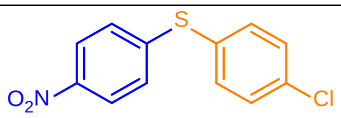	99
3	Br	120°C, 24h	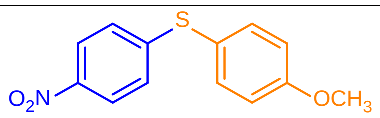	93
4	Br	120°C, 24h	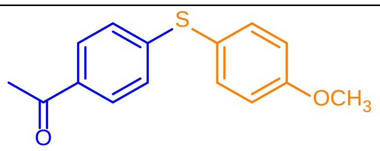	80
5	Cl	120°C, 24h	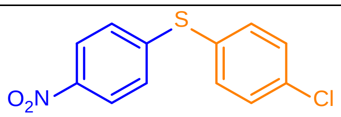	46
6	Cl	120°C, 72h	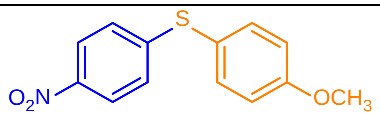	67

Supplementary Discussion

Relative stability of the nickel thiolates [Ni(SC_6H_5)$_2$] (**1a**) and [Ni(S*o*-$NH_2C_6H_4$)$_2$] (**1e**) was studied with DFT calculations. The values of ΔE and ΔG energies were calculated for the reaction of SPh group exchange to S(*o*-NH_2)C_6H_4 group in these thiolates (Supplementary Figure 79). Indeed, the obtained values of ΔE = -35.7 kcal/mol and ΔG = -34.3 kcal/mol indicated much stronger binding of the thiol group in the case of the Ni(S*o*-$NH_2C_6H_4$)$_2$ complex as compared to the Ni(SPh)$_2$ complex. A quite large energy change suggests exceptional stability of the Ni(S*o*-$NH_2C_6H_4$)$_2$ species.

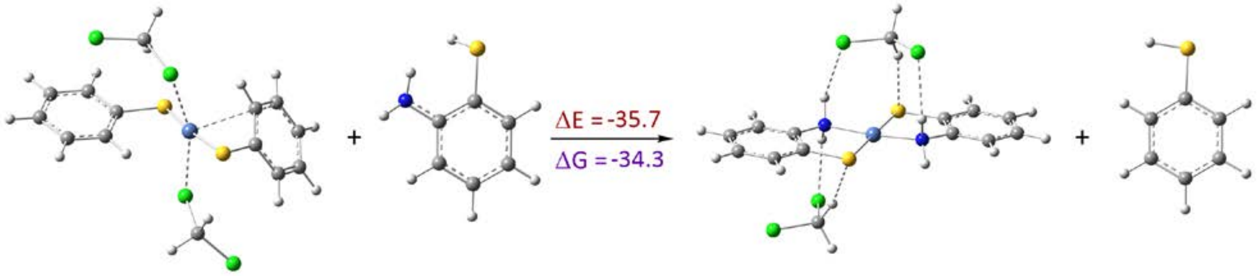

Supplementary Figure 79. DFT calculations for the reaction of thiolate group exchange in [Ni(SR)$_2$] complex leading to [Ni(S*o*-$NH_2C_6H_4$)$_2$] complex and calculated energy change (in kcal/mol) at UPBE1PBE/def2SVP GD3BJ level (two solvent molecules were also included).

Supplementary References

1. Swapna, K., Murthy, S.N., Jyothi, M.T., Nageswar, Y.V.D. Nano-$CuFe_2O_4$ as a magnetically separable and reusable catalyst for the synthesis of diaryl/aryl alkyl sulfides via cross-coupling process under ligand-free conditions. *Org. Biomol. Chem.* **9**, 5989-5996 (2011).

2. Wu, X.-m., Yan, G.-b. Copper nanopowder catalyzed cross-coupling of diaryl disulfides with aryl iodides in PEG-400. *Synlett* 537-542 (2015).

3. Wagner, A.M., Sanford, M.S. Transition-metal-free acid-mediated synthesis of aryl sulfides from thiols and thioethers. *J. Org. Chem.* **79**, 2263-2267 (2014).

4. Reddy, V.P., Swapna, K., Kumar, A.V., Rao, K.R. Indium-catalyzed C-S cross-coupling of aryl halides with thiols. *J. Org. Chem.* **74**, 3189-3191 (2009).

5. Lin, W., Sapountzis, I., Knochel, P. Preparation of functionalized aryl magnesium reagents by the addition of magnesium aryl thiolates and amides to arynes. *Angew. Chem. Int. Ed.* **44**, 4258-4261 (2005).

6. Zhang, X., Lu, G.-p., Cai, C. Facile aromatic nucleophilic substitution (S_NAr) reactions in ionic liquids: an electrophile–nucleophile dual activation by [Omim]Br for the reaction. *Green Chem.* **18**, 5580-5585 (2016).

7. Duan, Z., Ranjit, S., Liu, X. One-pot synthesis of amine-substituted aryl sulfides and benzo[b]thiophene derivatives. *Org. Lett.* **12**, 2430-2433 (2010).

8. Chen, X., She, J., Shang, Z., Wu, J., Zhang, P. A catalytic method for room-temperature Michael additions using 12-tungstophosphoric acid as a reusable catalyst in water *Synthesis* 3931-3936 (2008).

9. Panova, Yu.S., Kashin, A.S., Vorobev, M.G., Degtyareva, E.S., Ananikov, V.P. Nature of the copper-oxide-mediated C-S cross-coupling reaction: leaching of catalytically active species from the metal oxide surface. *ACS Catal.* **6**, 3637-3643 (2016).

10. Hong, B., Lee, J., Lee, A. Visible-light-promoted synthesis of diaryl sulfides under air. *Tetrahedron Lett.* **58**, 2809-2812 (2017).

11. Bu, M.-j., Lu, G.-p., Cai, C. Ascorbic acid promoted metal-free synthesis of aryl sulfides with anilines nitrosated in situ by tert-butyl nitrite. *Synlett* **26**, 1841-1846 (2015).

12. Sindhu, K.S., Thankachan, A.P., Thomas, A.M., Anilkumar, G. An efficient iron-catalyzed S-arylation of aryl and alkylthiols with aryl halides in the presence of water under aerobic conditions. *Tetrahedron Lett.* **56**, 4923-4926 (2015).

13. Ananikov, V.P., Kashin, A.S., Hazipov, O.V., Beletskaya, I.P., Starikova, Z.A. Highly selective catalytic synthesis of (E,E)-1,4-diiodobuta-1,3-diene via atom-efficient addition of acetylene and iodine: a versatile (E,E)-1,3-diene building block in cross-coupling reactions. *Synlett* 2021-2024 (2011).
