# Supplementary material for: Exploring the performance of nanostructured reagents with organic-group-defined morphology in cross-coupling reaction
Source: Nat Commun. 2018 Jul 26;9:2936. doi: 10.1038/s41467-018-05350-x (PMC6062554; doi:10.1038/s41467-018-05350-x)
Supplement: Supplementary file 2 — Descriptions of Additional Supplementary Files [file 41467_2018_5350_MOESM2_ESM.pdf]

Descriptions of Additional Supplementary Files

File Name: Supplementary Movie 1
Description: Liquid phase electron microscopy video of dissolution of $[Ni(S\textit{p}\text{-}BrC_6H_4)_2]_n$ (**1b**) particles in $Pd(OAc)_2$/dppe/EtOH system.

File Name: Supplementary Movie 2
Description: Liquid phase electron microscopy video of dissolution of $[Ni(S\textit{p}\text{-}BrC_6H_4)_2]_n$ (**1b**) particles in $Pd(OAc)_2$/dppe/EtOH system in the presence of iodobenzene.
